# CRI-SPA: a high-throughput method for systematic genetic editing of yeast libraries

**DOI:** 10.1093/nar/gkad656

**Published:** 2023-08-12

**Authors:** Paul Cachera, Helén Olsson, Hilde Coumou, Mads L Jensen, Benjamín J Sánchez, Tomas Strucko, Marcel van den Broek, Jean-Marc Daran, Michael K Jensen, Nikolaus Sonnenschein, Michael Lisby, Uffe H Mortensen

**Affiliations:** The Novo Nordisk Foundation Center for Biosustainability, Technical University of Denmark, Denmark; The Novo Nordisk Foundation Center for Biosustainability, Technical University of Denmark, Denmark; Department of Biotechnology and Biomedicine, Technical University of Denmark, Denmark; Department of Biotechnology and Biomedicine, Technical University of Denmark, Denmark; Department of Biotechnology and Biomedicine, Technical University of Denmark, Denmark; Department of Biotechnology and Biomedicine, Technical University of Denmark, Denmark; Department of Biotechnology, Delft University of Technology, Delft, The Netherlands; Department of Biotechnology, Delft University of Technology, Delft, The Netherlands; The Novo Nordisk Foundation Center for Biosustainability, Technical University of Denmark, Denmark; Department of Biotechnology and Biomedicine, Technical University of Denmark, Denmark; Department of Biology, University of Copenhagen, Copenhagen, Denmark; Department of Biotechnology and Biomedicine, Technical University of Denmark, Denmark

## Abstract

Biological functions are orchestrated by intricate networks of interacting genetic elements. Predicting the interaction landscape remains a challenge for systems biology and new research tools allowing simple and rapid mapping of sequence to function are desirable. Here, we describe CRI-SPA, a method allowing the transfer of chromosomal genetic features from a CRI-SPA Donor strain to arrayed strains in large libraries of *Saccharomyces cerevisiae*. CRI-SPA is based on mating, CRISPR-Cas9-induced gene conversion, and Selective Ploidy Ablation. CRI-SPA can be massively parallelized with automation and can be executed within a week. We demonstrate the power of CRI-SPA by transferring four genes that enable betaxanthin production into each strain of the yeast knockout collection (≈4800 strains). Using this setup, we show that CRI-SPA is highly efficient and reproducible, and even allows marker-free transfer of genetic features. Moreover, we validate a set of CRI-SPA hits by showing that their phenotypes correlate strongly with the phenotypes of the corresponding mutant strains recreated by reverse genetic engineering. Hence, our results provide a genome-wide overview of the genetic requirements for betaxanthin production. We envision that the simplicity, speed, and reliability offered by CRI-SPA will make it a versatile tool to forward systems-level understanding of biological processes.

## Graphical Abstract

**Graphical Abstract:**
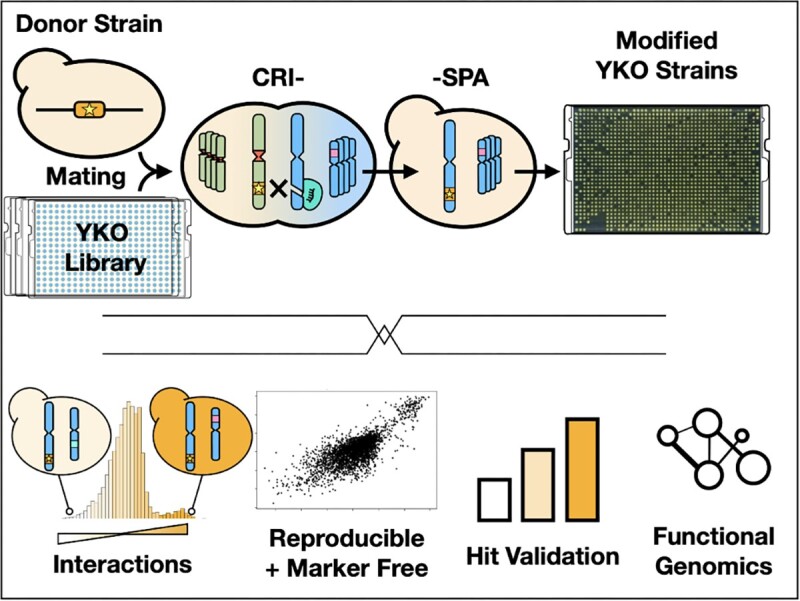

## INTRODUCTION

Baker's yeast *Saccharomyces cerevisiae* is an important biological model organism and a key production host in industrial biotechnology. *S. cerevisiae* was the first eukaryote to be fully sequenced (1,2), and its simple lifecycle as a single cell organism with a highly developed genetic toolbox has positioned this yeast as a frontrunner in the field of systems biology. However, the unpredictability of phenotypic effects resulting from combinations of different genetic traits still challenges our fundamental understanding of yeast and its engineering. The introduction of CRISPR for yeast engineering (3) promises to relieve this bottleneck by accelerating the systematic construction and testing of genetic variants. For example, in numerous genome-wide engineering projects, CRISPR-based strategies have been developed to create and screen libraries of strains (4–11). These screens generally follow a one-pot workflow where the library is pooled, edited and challenged before being resolved by FACS and next generation sequencing (12,13). However, library pooling is prone to bias as it selects for fast growth, and traits of interest might be lost if they are accompanied by a reduction in growth fitness. Similarly, when the best candidates are recovered from a one-pot screen, the top pool might be saturated by a few strongest variants, which limits the full network of interactions to be uncovered (14). A more useful output would be achieved if the members of the library could be assessed individually to produce the full overview of the genetic effects impacting the system.

Aside from one-pot approaches, screening can be achieved in a systematic manner by introducing a genetic modification in all strains of an existing yeast library (15,16); and to date, Synthetic Genetic Array (SGA) has been the state of the art method for such systematic analyses (17). SGA queries strains individually in a process based on arrayed mating, meiosis, sporulation and marker selection for the desired gene combination. This method has been particularly valuable for the identification of genetic fitness interactions among all double and a selected number of triple gene knock-outs (18,19). However, the reliance of SGA on meiosis is tedious, and the dependency of markers introduces experimental limitations. For example, sporulation is slow, 4–7 days, and the efficiency is difficult to control. Moreover, the high level of meiotic recombination required to ensure correct chromosome segregation in the first meiotic division (20) is per se undesirable. Firstly, as meiotic recombination mixes donor and recipient genomes, the genetic background of the donor and recipient strains need to be identical. Secondly, due to meiotic segregation, all genetic features need to be flanked by a genetic marker allowing selection of the desirable gene combination after meiosis. Such markers may influence expression of neighboring genes; and if many genes are studied, become a limitation for multiplexing. Thirdly, the high levels of meiotic Homologous Recombination (HR) may also set the stage for unwanted chromosome rearrangements if the strains suffer from repeated use of genetic elements, e.g. promoters. Lastly, flawed meiosis is known to frequently generate aneuploids (21,22), which may complicate further analyses. A faster method, independent of meiosis and marker selection, is therefore desirable.

**Figure 1.**
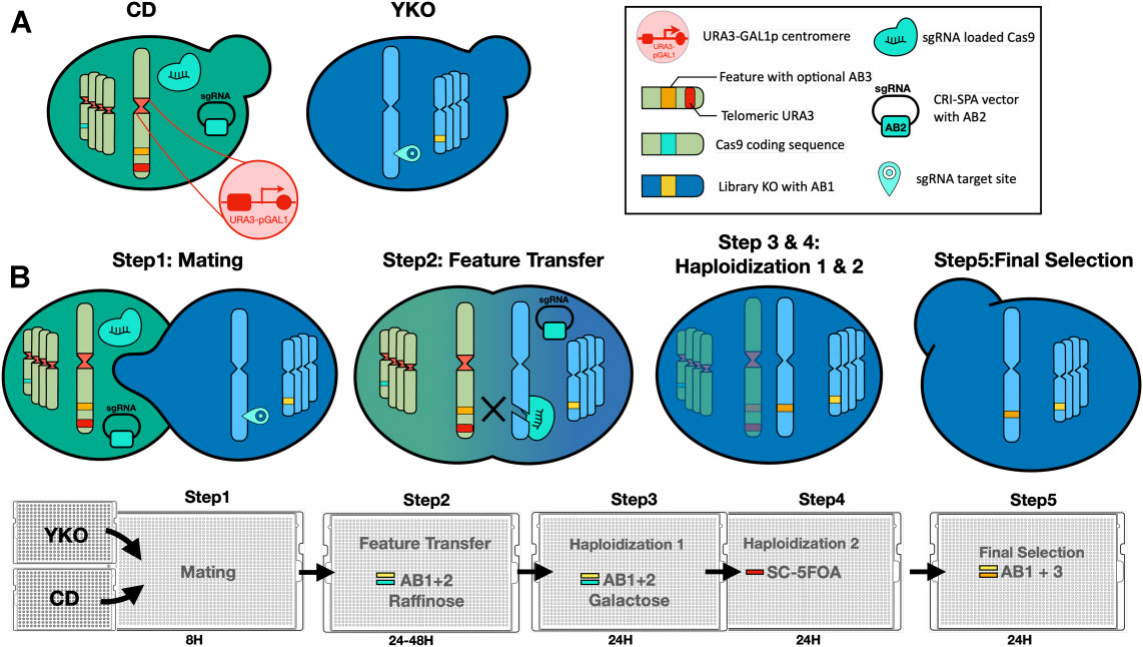
The CRI-SPA gene-transfer system. (**A**) Left: The CRI-SPA Donor (CD) strain and the recipient library strains (here YKO). Right: Graphics of the individual genetic components are shown in the legend box. The YKO strains contain a specific deletion marked by the antibiotic marker 1 (AB1) and a target site for the Cas9-sgRNA CRISPR nuclease. The CD strains contain: *Kl_URA3-GAL1p* cassettes at the centromeres of all chromosomes, a *cas9* gene, a selectable CRI-SPA plasmid maintained by antibiotic marker (AB2) and encoding a sgRNA targeting the insertion site of the genetic feature (blocked in the CD), a genetic feature of interest coupled to an antibiotic marker, AB3 (optional), and a *Kl_URA3* gene between the genetic feature of interest and the telomere. (**B**) The individual steps of the CRI-SPA procedure (see main text for details). **Step 1**, a CD strain is pinned onto all strains of the library plates and incubated for mating. **Step 2**, diploids are exhausted for glucose via growth on raffinose and selected for the marker in the library strains (AB1) and the marker on the CRI-SPA plasmid (AB2). In the diploid stage, the target site in the recipient strain is cleaved by Cas9-sgRNA. Repair of the resulting DNA DSB by gene conversion using the corresponding donor site in the CD strain as template transfers the genetic feature to the recipient locus. Note that the target site of the Cas9-sgRNA in the recipient chromosome is destroyed by insertion of the genetic feature of interest. **Step 3**, galactose induced SPA eliminates the donor chromosomes. **Step 4**, haploid cells containing only recipient chromosomes are selected on 5-FOA. **Step 5**, the modified library strains are obtained by selecting for AB1, which marks the YKO deletion, and optionally, by selecting for the genetic feature (AB3).

We have developed a new screening platform that allows a genetic trait to be queried in large arrayed yeast libraries. Our method (Figure 1), CRI-SPA, combines Clustered Regularly Interspaced Short Palindromic Repeats (CRISPR) technology (23); (3) with selective ploidy ablation, SPA (24). In CRI-SPA, a (marker-free) genetic feature of interest is efficiently transferred from a single donor strain to the strains in a library in a process involving mating, Cas9 induced gene conversion, and haploidization by SPA. CRI-SPA can be massively parallelized using a pinning robot and executed in less than a week with as little as 4 hours handling time. Since it relies on image analysis for data extraction, the main cost associated with CRI-SPA comes from media and plastic consumables. Here, we use the method to transfer four genes responsible for the synthesis of the yellow plant metabolite betaxanthin, a biosensor for the morphine precursor L-DOPA (25) into the 4800 strains of the yeast knock-out (YKO) library (26). We demonstrate that multiple-gene transfer by CRI-SPA can be performed with and without selection in a highly reproducible manner. As a result, we unveil the full betaxanthin pathway-host gene interaction landscape and most prominently demonstrate that mutations impairing mitochondrial functions consistently influence betaxanthin levels. Finally, we show that CRI-SPA scores obtained in screening conditions correlate well with betaxanthin levels of reversed engineered hits in liquid medium.

## MATERIALS AND METHODS

### Strains and media

All strains constructed in this work are listed in Supplementary Table S1. Strains W8164-2B and W8164-2C of the SPA method (27) were kindly provided by Rodney Rothstein (Department of Genetics and Development, Columbia University, USA). The YKO library was acquired from Invitrogen. YPD and synthetic complete (SC) media, and SC drop out media were prepared as described by Sherman et al. (28), but with 60 mg/l l-leucine. For *URA3* counter selection, SC plates were supplemented with 1 g/l 5-fluoroorotic acid (5-FOA) and 30 mg/l uracil. To enhance the red phenotype of *ade2* cells, SC medium with only 4 mg/l adenine was used. To prevent *ade2* cells to be outcompeted by *ADE2* cells, growth media were supplemented with 40 mg/l adenine. Galactose and raffinose solutions were sterilized by filtration and used at 2% final concentration. For growth on solid medium, 20 g/l agar was added. For selection on solid medium, plates were supplemented with 200 mg/l geneticin (G418), 100 mg/l nourseothricin (NTC) and/or 200 mg/l hygromycin (HYG) as indicated. Half the concentration of the respective antibiotics were used in liquid cultivations. All strains were validated by diagnostic colony PCR to ensure correct integration at the intended chromosomal locus. For an experimental overview of CRI-SPA (CD) Donor strains construction, see Supplementary Figure S1.

### Plasmid construction and PCR

Generally, DNA assembly was done by USER-fusion (29,30) employing uracil-specific excision reagent (USER™) enzyme from New England Biolabs. PCR fragments were generated using Phusion U Hot Start DNA Polymerase (Thermo Fisher). Details for construction of individual plasmids are provided in Supplementary Methods S1. All plasmids and primers used in this study are presented in Supplementary Table S2 and Supplementary Table S3, respectively. All plasmid maps are available for download as GenBank files.

### Construction of yeast strains

All yeast transformations were carried out using the LiAc/SS Carrier DNA / PEG method described by Gietz and Schiestl (31). When antibiotic resistance genes were used as markers, cells were allowed to recover for 2 hours in liquid YPD prior to plating on solid selective media.

Universal CRI-SPA strains, UC strains, were made by inserting a gene-targeting substrate containing *cas9* and *LEU2* into chromosomal integration site X-3 of W8164-2B (*MATα*) and W8164-2C (*MAT***a**). The gene-targeting substrate was liberated from pHO8 by digestion with NotI (Thermo Scientific). The UC+:XII-5α strain (see Supplementary Figure S1 and Supplementary Table S1) was made from the UC strain (UC1α) by inserting an additional copy *of Kl_URA3* between expression site XII-5 and the telomere. Insertion was achieved via co-transforation with sgRNA plasmid pHO25 and a linear *Kl_URA3* repair substrate liberated from pCfB390 (32) using primers HOP181 and HOP182. CRI-SPA donor (CD) strain were made from UC strains. CD-*ade2*Δ was constructed by first transforming the UC strain (UC1α) with sgRNA plasmid pCfB2311 (33) and a linear repair substrate for disruption of *ADE2* via insertion of the *hphNT1* cassette, which was generated by PCR using plasmid pMEL12 and primers HOP64 and HOP65. The resulting strain was then further modified to harbor an additional *Kl_URA3* marker between the disrupted *ADE2* gene and the telomere by transformation with sgRNA plasmid pHO22 and a linear *Kl_URA3* repair substrate amplified from pCfB390 by primers HOP175 and HOP176, yielding CD-*ade2*Δ.

CD-Btx was constructed from UC+:XII-5α by transforming with the integrative plasmid pBTX1 harboring the betaxanthin-synthesizing genes CYP76AD1 and DOD, an NTC selectable marker, negative feedback resistant *ARO4^K229L^, ARO7^G141S^* and an sgRNA expression cassette for targeting the XII-5 site. The BY-Btx strain was made by transforming the NotI excised insert of pBTX2, coding for CYP76AD1 and DOD and the negative feedback resistant *ARO4^K229L^, ARO7^G141S^*, into strain BY4741.

Introduction of CRI-SPA-hit knockouts into the BY-Btx strain was performed by replacing their Open Reading Frame (ORF) with a *kanMX* cassette (34). The *kanMX* cassette was amplified from pCfB2312 (32) and fused to targeting sequences up- and downstream of the relevant ORF. For the first round of KOs (DLD2, KGD2, MIP1, RCR2, YER084W, COX12, QCR10, SHY1, SGM1, VPS34, SSM4, UBC7, RPN4), the 400 bp up- and downstream targeting sequences were amplified using BY4741 genomic DNA as a template and by using primers with 40bp overhangs homologous to the *kanMX* cassette for amplification. The upstream, downstream, and *kanMX* amplicons were transformed into BY-Btx and fused *in vivo* by HR. For the second round of KOs (PRO2, VMA16, GET4, PRY1, STV1, YCR101C, YLR271W, GLR1, HEM25), the up and down homology sequences of the relevant ORFs were fused to the *kanMX* cassette by USER cloning. USER primers were used to generate PCR fragments of the up- and downstream sequences of the ORFs using genomic DNA from BY4741 as template. The relevant PCR fragments were USER-fused (29) to the *kanMX* cassette in *Escherichia coli* and inserted into the USER cloning site of pCCM023, a modified version plasmid pCfB2909 lacking XII5-up and XII5-down homology sequences. The Up::*kanMX*::Dw gene-targeting substrates were released from purified plasmids by digestion with NotI prior to transformation into BY-Btx. All transformant BY-Btx strains were selected on solid YPD medium containing 200 mg/l G418. All KOs were verified by PCR amplifying the upstream and downstream integration sites of the *kanMX* cassette. For both amplifications, a primer annealing outside and a primer annealing inside of the cassette were used.

### CRI-SPA high-throughput pin replication protocol

Automatic pin replication was carried out using the high throughput pinning robot ROTOR HDA from Singer Instruments (United Kingdom), along with replica pinning pads (RePads) and rectangular petri dishes (PlusPlates) from the same company.

For parallel mating of the CD Strain to the strains of the yeast deletion library, a single colony of the UDS was inoculated in 25 ml YPD supplemented with the appropriate antibiotic in order to maintain selective pressure for the gRNA plasmid. The UDS was grown overnight, washed by centrifugation, and resuspended with 25mL sterile water to remove the antibiotic. A volume of 150 μl of cell suspension was dispensed in wells of a 96-well plate serving as a source plate for the screen. From this source plate, the UDS was pinned in 1536 format on the mating plates. The arrayed strains of the deletion collection were pinned on top of the UDS. Plate shuffling was introduced at this stage to address experimental artifacts (see below). Mating was allowed to proceed from 8 h to overnight. Strains were then pin replicated onto a series of selective plates as outlined in Figure 1.

### Image acquisition and processing

The data from our screen was extracted from images acquired with a specialized imaging system (Phenobooth, Singer) keeping acquisition parameters fixed (lighting power, camera brightness, gain, exposure, hue, saturation, white balances). To extract yellow color intensity, we applied a heuristic filter taking the geometric mean of Value and Saturation of image pixels in the HSV color space (Supplementary Figure S2A). This assumed that the colony's Hue was fixed (i.e. a colony can only harbour variants of yellow) which was our observation on YPD media.

We repurposed the mask functions of an image analysis package (35), to assign pixels to colonies on a plate image. The number of pixels assigned to a colony was used as a measure of colony size. Pixels belonging to a colony were filtered and the average filtered colony pixel value was used as the yellow intensity score.

### Randomisation of positional artifacts, outlier removal, data normalization

We observed that colony size and yellowness score were subject to positional effects known to affect colony size in high density array screens (36). We adopted a colony position randomization strategy to randomize plate, agar quality and neighbor effects. This randomization was done when pinning the YKO library from its storage 384 format to the screen's 1536 quadruplicate format. Each one of the four 384 arrays from a given library plate was pinned on a different screening plate (Supplementary Figure S2B). As a result, each colony quadruplicate was positioned on a different screen plate and exposed to randomized agar qualities and neighbor effects. Still, row-column colony positions were not altered by this randomization, and colonies were therefore still affected by non-random edge effects. These were corrected by setting the median of colonies in outer frames equal to that of center colonies (37). After edge correction, the screen's data was centered by subtracting the screen's mean from each data point and normalized by dividing all data points by the standard deviation of the screen. After this normalization, outliers within quadruplicates were detected with Grubbs' test for outliers and deleted. Genes represented by two colonies or less were deleted from the analysis. Finally, the mean of the colony replicate was used as the final score for each gene.

### Combining the data of multiple screens

To combine the data of different screens, the edge-corrected data for each individual screen was normalized by subtracting the screen mean and by dividing colony scores by the screen standard deviation. At this point, the individual datasets were pooled and outliers removed with Grubbs’ test. After outlier removal, mean yellow intensity and size of gene-deletion strains with three or more colonies was used as the final yield and fitness, respectively, (pipeline and data can be accessed on the repository https://github.com/pc2912/CRI-SPA_repo).

### Betaxanthin fluorescence quantification

Fluorescence in SC medium was measured by a Synergy Mx plate reader (Agilent BioTek) using excitation of 465/20 nm and emission of 525/20 nm as described previously by Savitskaya *et al.* (14).

### Gene enrichment analysis and GO term graphs

Gene enrichment analysis was performed using GOATOOLS (38,39) to generate the gene groups indicated in Figure 5. The default Benjamini-Hochberg correction was used to account for the number of tests, and a P-value of 0.05 was used as a threshold to accept GO terms as enriched. For visualizing results at a systems-level, a graph was constructed where nodes representing GO terms were connected if they shared associated genes. The size of the nodes was drawn as a function of the significance of the term enrichment and the edge opacity was drawn in relation to the number of genes shared between terms. Nodes were colored according to their description or the description of their ancestors (i.e. descriptions contained key words such as ‘mitochondria’, ‘translation’, ect.). Finally, the resulting networks were built in networkX, transferred to Cytoscape (40) with py4cytoscape and minor manual adjustments were made for visibility (scripts available at https://github.com/pc2912/CRI-SPA_repo).

### Statistical tests

Statistically significant overlaps of hits identified in screen A and screen B were determined by using a test based on hypergeometric distribution: p_h(*x* ≥ *k* | *M*,*n*,*N*). *M* is the number of genes quantified in both screen A and screen B, and *n* = 192 is the number of genes defined as hits in Screen A. *N* = 192 is the number of genes defined as hits in screenB. Finally, *k* is the number of overlapping gene hits between the two screens. Statistically significant increases (or decreases) in yellowness and fluorescence between BY-Btx and BY-Ref were determined by a one-sided *t*-test. Significant differences in yellowness score on solid YPD medium and fluorescence in SC medium between BY-Btx and reversed engineered hit mutants were identified with a one-sided Welsh-tests.

## RESULTS

### Basic components of the CRI-SPA system

CRI-SPA is based on four components: (i) Universal CRI-SPA strains (UC strains), see Supplementary Table S1, which are SPA strains (24) equipped with a *cas9::LEU2* cassette, see Materials and Methods, in integration site X-3 (41). A SPA strain contains a *Kl_URA3*-*GAL1*p cassette next to all of its sixteen centromeres; and all chromosomes of a SPA strain can therefore be destabilized on media containing galactose. Hence, in diploids where a SPA strain serves as one of the parent strains, SPA chromosomes can be selectively eliminated on galactose medium followed by counterselection on 5-FOA (24). As a result, a haploid strain composed by chromosomes solely originating from the non-SPA parent strain is achieved. We have constructed UC strains of both mating types. (ii) CRI-SPA vectors, see Supplementary Table S2, which are selectable 2μ based plasmids encoding a desirable gRNA. CRI-SPA vectors enable formation of Cas9–gRNA complexes that serve two purposes. Firstly, they mediate integration of a genetic feature of interest into a specific site in a UC strain; and secondly, they enable HR mediated transfer of the genetic feature during CRI-SPA (see below). In addition, the marker gene on the CRI-SPA vector facilitates selection for diploid cells during the CRI-SPA procedure, see below. (iii) CRI-SPA Donor strains (CD), see Supplementary,Table S1, which are UC strains that contain the genetic feature of interest in a defined chromosomal locus, a CRI-SPA vector encoding a gRNA matching the wild-type sequence of the modified locus, and a *Kl_URA3* gene between the genetic feature of interest and the telomere of the modified chromosome. This additional *Kl_URA3* gene is used to counterselect undesired recombination events that may accompany Cas9 induced gene conversion, see below and Supplementary Figure S3. (iv) A CRI-SPA compatible recipient strain library. The strains in the library need to contain a genetic marker allowing selection for diploid cells when they are mated to the CD strain. Moreover, in the current version of our CRI-SPA system, the library needs to be composed of strains that are *ura3* as SPA depends on counterselection of *Kl_URA3*. In the present study, we have used the genome-wide YKO collection as the recipient library (26). The strains in this library are all *ura3Δ0* and the individual gene deletions are marked by *kanMX* (42), which can be selected by addition of G418 to the medium.

### Experimental steps of the CRI-SPA method

The CRI-SPA procedure is performed in five steps during six days (Figure 1). In step 1, The CD strain and the recipient library strains mate overnight on YPD medium to form diploid cells. In step 2, diploid cells are selected via a complementary marker setup for 48 h on solid raffinose. In the present study, this was achieved by replica-pinning the colonies from YPD to solid raffinose medium containing G418 and either nourseothricin (NTC) or hygromycin (HYG) to select for *kanMX* marked deletions delivered by the parental library strains and the *natMX*- or *hphNT1*-based CRI-SPA plasmid delivered by parental CD strains, respectively. In diploid cells, the Cas9-sgRNA CRISPR nuclease produces a DNA DSB at the recipient locus in the library strain chromosome, and this DNA DSB is repaired through HR using the corresponding modified locus of the CD strain as a repair template. Most commonly, repair is expected to proceed via synthesis-dependent strand-annealing (43) resulting in desirable transfer of the genetic feature from the donor to the recipient chromosome by gene conversion. However, we note that undesirable chimeric donor/recipient chromosomes may be produced as the result of repair by gene conversion accompanied by a crossover or if repair is mediated by break induced replication (44). In CRI-SPA, these undesirable repair outcomes are counter-selected in step 4 (also see Supplementary Figure S3). The suboptimal and non-repressing carbon source raffinose (45) was used to exhaust glucose and to provide a slow growth step thereby allowing more time for CRISPR mediated gene transfer compared to growth on glucose. In step 3, the absence of glucose repression allows for the sudden induction of the *GAL1* promoter as the colonies are replica-pinned from raffinose to solid galactose medium (45). Active *GAL1* promoters disrupt the centromeres of all CD strain chromosomes, hence, inducing CD strain chromosome loss as the cells are dividing (24). In step 4, cells that have lost all donor chromosomes are selected by transferring the strains to solid SC medium containing 5-FOA. Note, this medium also counterselects undesired recombinant strains resulting from DNA DSB repair involving crossing-over or break-induced replication at the target locus (Supplementary Figure S3). As a result, step 4 generates haploid strains that solely contain the chromosomes originating from the recipient strain as well as the genetic feature of interest. In the final step 5, recipient cells are selected by the marker of the library, in this case by the *kanMX* marker of the YKO library to eliminate CD cells (if any) that did not mate, and which survived *kanMX* and 5-FOA selection. Optionally, the genetic feature of interest may also be selected for if it is accompanied by a marker. This may eliminate unmodified recipient cells (if any) that have escaped the selection step for diploids. Conveniently, we note that CRI-SPA can be executed by hand or scaled with a pinning robot to accommodate a range of budgets and throughputs. A more detailed scheme describing the five CRI-SPA steps is provided in Supplementary Methods S2.

### CRI-SPA donor strain construction and test for cas9 induced DNA DSB formation efficiency

CD strains are constructed in three simple steps, see Supplementary Figure S1. Firstly, an additional *Kl_URA3* marker is inserted into a locus between the integration site of the genetic feature of interest and the telomere. This is achieved in a CRISPR mediated process based on a gRNA plasmid expressing the appropriate gRNA. Secondly, the resulting strain is cured of the gRNA plasmid. Finally, the CD strain is obtained by inserting the genetic feature into the desired locus in a second CRISPR mediated reaction assisted by the CRI-SPA vector.

Successful CRI-SPA mediated allele transfer depends on highly efficient DNA DSB induction at the target locus by a CRISPR nuclease directed by a specific gRNA encoded from the CRI-SPA vector. Hence, prior to a CRI-SPA experiment, it is important to demonstrate the efficiency of a desired CRISPR nuclease/gRNA complex. This can be judged by performing a TAPE (Technique to Assess Protospacer Efficiency) experiment (46), which examines lethality of a UC strain after transformation with a CRI-SPA plasmid in the absence or presence of an efficient repair template. Compared to a reference plasmid, a CRI-SPA vector suitable for a CRI-SPA experiment needs to produce transformants at numbers that are several fold higher in the presence than in the absence of a repair template, see Figure S4. Note that the TAPE test can conveniently be combined with CD strain construction by using a repair template that is designed to introduce the genetic feature of interest into the desired locus of the UC strain.

### Transfer of ade2δ::hphNT1 into a set of arrayed gene-deletion mutants

To examine the potential of CRI-SPA, we first investigated whether it is possible to transfer a HYG selectable *ade2*Δ*::hphNT1* cassette from a CD-*ade2*Δ strain to a subset of mutants from the YKO library containing G418 selectable gene-deletion cassettes. Advantageously, *ade2Δ* strains produce an easy-to-score red phenotype (47,48), which may be epistatically blocked by mutations upstream in the purine pathway, e.g. *ade3* (49). Successful interchromosomal transfer of *ade2*Δ*::hphNT1* into YKO library mutants should therefore produce mostly red colonies, but should also be able to identify epistatic genetic interactions, like *ade3*Δ, which should appear as white colonies. Hence, we set out to investigate whether CRI-SPA would be able to quickly identify mutants that are epistatic to *ade2* by screening for double mutants that form white colonies.

The CD-*ade2*Δ strain was constructed as part of a TAPE test demonstrating that the NTC selectable *ADE2* CRI-SPA vector (pHO24) is able to produce a gRNA that allows Cas9 to efficiently cleave the *ADE2* locus (Supplementary Figure S4). Specifically, we obtained >6-fold more transformants in the presence (915 transformants) than in the absence (145 transformants) of the *ade2*Δ*::hphNT1* cassette repair template. Next, the resulting CD-*ade2*Δ strain was used in a CRI-SPA experiment to screen the strains on plate 9 of the YKO library (*MATa*). This plate contains 376 gene deletions, including the negative interaction control *ade3*Δ::*kanMX*. The *ade2*Δ*::hphNT1* transfer experiment was performed as a 2 × 2 quadruplicate using 1536 format. In all growth steps, except the final one, media was supplemented with additional adenine to reduce the negative fitness effects due to the *ade2* deletion (50). On the final plate, the medium contained reduced adenine levels to speed-up development of red color, see MATERIALS AND METHODS.

**Figure 2.**
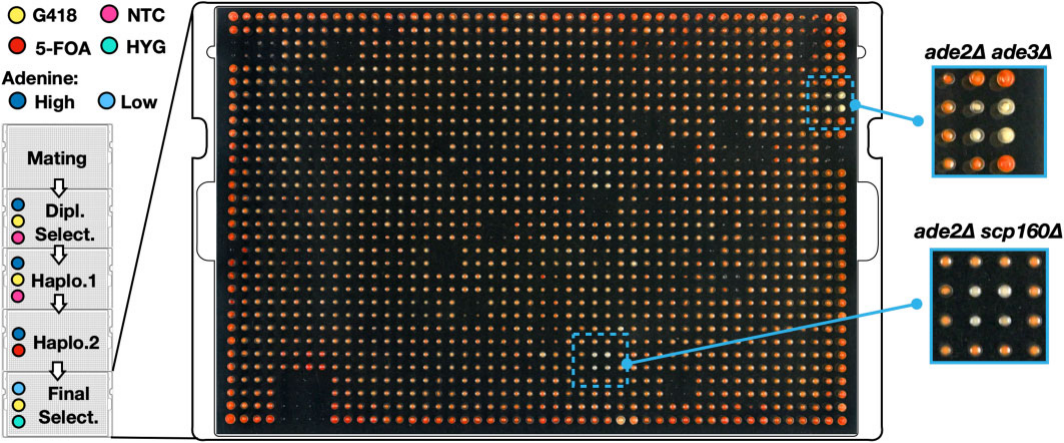
Transfer of the *ade2*Δ*::hphNT1* cassette from a CD to plate 9 of the YKO library. CRI-SPA mediated transfer of the *ade2*Δ*::hphNT1* cassette to the mutant strains of plate 9 of the YKO library was performed in quadruplicate. The final CRI-SPA plate was photographed and shown in the middle. Selection applied at the different CRI-SPA steps are indicated to the left by the color code. G418 selects for the *kanMX* marker in the gene deletion cassettes of the YKO library, NTC selects for the CRI-SPA plasmid, HYG selects for the *ADE2* disruption cassette *ade2*Δ*::hphNT1*, and 5-FOA counter-select donor strain chromosomes, which all contain *Kl_URA3* markers. The four *ade2*Δ*::hphNT1 scp160*Δ::*kanMX* and the four *ade2*Δ*::hphNT1 ade3*Δ::*kanMX* CRI-SPA colonies are framed by blue squares. Magnifications of the *ade2*Δ*::hphNT1 ade3*Δ::*kanMX* quadruplets are shown to the right as indicated.

After the CRI-SPA experiment, virtually all quadruplicates were red indicating that the *ade2*Δ*::hphNT1* cassette was efficiently transferred to the vast majority of all mutant strains of the library, see Figure 2. Only a few mutant strains did not produce any growth after CRI-SPA and such mutants may represent strains that failed to go through CRI-SPA, e.g. if they failed to mate. For a few quadruplicates, some of the colonies appeared white/pinkish. Further restreaking on solid selective media (G418 and HYG) demonstrated that they contained a mix of cells that were able to form white and red cells. Moreover, cells forming white colonies were able to grow in the absence of adenine and diagnostic PCR demonstrated that their genotype was *ADE2* and *ade2*Δ*::hphNT1*. Further, full genome sequence analysis of selected purified white strains showed that they were aneuploid or diploid strains containing two copies of the target chromosome of which only one has received a copy of the *ade2*Δ*::hphNT1* cassette, see Supplementary Figure S5. Formation of such cells may reflect that many strains in the gene deletion library suffer from aneuploidy (51). Some mutants may be particularly prone to this phenomenon and the most prominent case of a ‘false whitish’ phenotype was observed for *scp160*Δ, which is known to display ploidy instability (47,52–53). Alternatively, they may be derived from diploid cells resulting from endoreduplication of the recipient genome, which may happen during SPA (27), see Supplementary Figure S6. Although formation of *ADE2*/*ade2*Δ*::hphNT1* cells potentially could complicate the CRI-SPA analysis as they formed white cells, in particular as they display a fitness advantage over *ade2*Δ*::hphNT1* cells, this was generally not the case. Indeed, the *ade2*Δ*::hphNT1 ade3*Δ::*kanMX* quadruplet was easily identified as the only example of a quadruplet where all four colonies were purely white, see Figure 2. Hence, we believe that formation of this cell type is quite rare and they may likely only impact experiments if they display a significant fitness advantage. Altogether, we conclude that that the CRI-SPA method was able to efficiently transfer the *ade2*Δ*::hphNT1* cassette from a CD to a recipient library and that it was able to robustly identify the negative *ade2*Δ*::hphNT1 ade3*Δ::*kanMX* interaction.

### Establishment of an efficient CRI-SPA based betaxanthin screening platform

**Figure 3.**
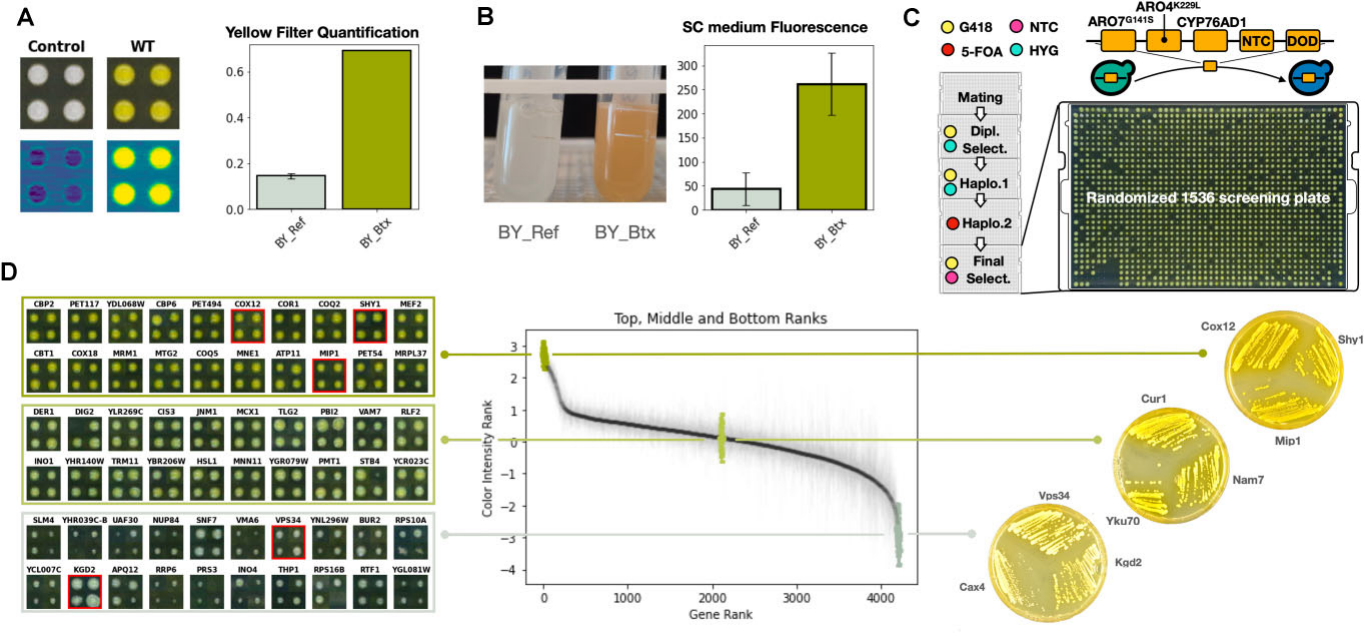
Betaxanthin production in YKO strains. (**A**) Left: images of BY-Ref and BY-Btx colonies in visible light (top) and corresponding filtered images (bottom). Right: quantification of yellowness of BY-Ref and BY-Btx colonies by image analysis, see main text and Supplementary Figure S2 for details. (**B**) Left: image of BY-Ref and the BY-Btx strains in liquid SC medium. Right: quantification of fluorescence produced by BY-Ref and the BY-Btx strains in liquid SC medium. (**C**) Final plate of a CRI-SPA Btx-cassette transfer experiment. At the top, a scheme illustrating the Btx-cassette and how it is transferred from the CD strain to a library recipient strain. To the left, diagrams with color codes indicating selection applied at the different CRI-SPA steps. G418 selects for the *kanMX* marker in the gene-deletion cassettes of the YKO library, HYG selects for the CRI-SPA plasmid, NTC selects for the Btx-cassette, and 5-FOA counter-select donor strain chromosomes, which all contain *Kl_URA3* markers. (**D**) In the middle, yellow intensity ranking of all YKO mutant strains after CRI-SPA mediated Btx-cassette transfer. To the left, mosaic images of the four individual replicates obtained with selected YKO mutants displaying higher, similar, or lower yellowness as compared to BY-Btx. Mutants in red frames were purified and restreaked on solid YPD medium, see images to the right.

We next sought to demonstrate the usefulness of the CRI-SPA technology in a metabolic engineering context. We attempted to map out the genetic requirements, at single gene resolution, for the production of betaxanthin, a plant metabolite derived from the shikimate pathway. Betaxanthin is yellow and fluorescent; and has previously been used as a biosensor and a proxy for production of the morphine precursor L-DOPA in yeast (25). To test whether betaxanthin production could be meaningfully evaluated in a CRI-SPA screen using the YKO library, we firstly introduced a five-gene betaxanthin-cassette (Btx-cassette) into BY4741, producing strain BY-Btx. The Btx-cassette harbors *CYP76AD1* and *DOD* (25), which are required for production of betaxanthin, the dominant mutant genes *ARO4^K229L^, ARO7^G141S^*, which alleviate the negative feedback regulation of the shikimate pathway (25); (54), as well as the selectable *natMX* marker. As expected for a strain producing betaxanthin, BY-Btx, unlike the reference strain BY-Ref (BY4741), produced yellow colonies (Figure 3A); and liquid cultures were yellow and fluorescent (Figure 3B). Importantly, betaxanthin production did not appear to influence fitness, as BY-Btx displayed essentially the same growth rate as BY-Ref Supplementary Figure S7.

We next tested whether significant differences in betaxanthin production in the YKO library background could be scored in a simple colony-based assay suiting the format and throughput of a CRI-SPA experiment. In our hands, the colony color phenotype was the easiest to score with a color filter, extracting color intensity from RGB pixel values, see ‘Image acquisition and processing’ in Materials and Methods and Supplementary Figure. S2A). Using this method, we demonstrated that the yellowness-score of colonies obtained with the betaxanthin producing strain was around 5-fold higher (*P* ≤ 10e–10) than the score obtained with colonies of the reference strain, see Figure 3A. We also examined production of betaxanthin in liquid SC-medium by measuring betaxanthin fluorescence in a plate-reader assay using a method previously described by Savitskaya *et al.* (14). In this setup, the BY-Btx strain was 6-fold (*P* ≤ 0.007) more fluorescent than the reference strain, see Figure 3B, demonstrating that the method could be used as a complementary assay for characterizing specific Btx-hit strains identified by yellow color development on solid medium. In summary, we have established a readily scalable image analysis method able to quantify betaxanthin production based on yellow color intensity; and we note that yellow color and fluorescence are widely accepted betaxanthin detection methods (14,25,55).

To set the stage for a genome-scale betaxanthin CRI-SPA experiment, we firstly constructed a betaxanthin CD (CD-Btx) that harbors the NTC selectable five-gene Btx-cassette in the XII-5a expression site, a *hphNT1* selectable CRI-SPA plasmid (pHO29), which encodes a gRNA that efficiently targets empty XII-5a sites (56), and a *Kl_URA3* marker positioned between XII-5a and the telomere. Secondly, given the considerable size, 9.3kb, of the Btx-cassette we performed a pilot experiment to assess how efficient it is transferred from the CD-Btx strain to the mutant strains of plate 9 of the YKO library, which we also used in the *ade2::hphNT1* transfer experiment described above. Lastly, as the Btx-cassette, unlike *ade2*Δ*::hphNT1*, does not confer a significant fitness defect in BY-Btx the YKO reference strain, we decided to explore the possibility of transferring the Btx-cassette without selecting for its *natMX* marker. Hence, we performed the pilot CRI-SPA Btx-cassette transfer experiment in quadruplicate in the absence of nourseothricin. Out of the 376 strains on the library plate, 373 strains (>99%) produced colonies after CRI-SPA, and 367 (>97%) produced all four colonies, see Supplementary Figure S8. Encouragingly, the vast majority of colonies were yellow indicating highly efficient transfer of the Btx-cassette despite that we did not apply nourseothricin for its selection during any of the steps in the CRI-SPA procedure.

CRI-SPA colonies are not clones, and strain-to-strain betaxanthin production comparisons can therefore be compromised if some colonies are contaminated with significant amounts of other cell types producing levels of betaxanthin that are different as compared to the levels of the modified recipient mutant cells. Since we did not select for the Btx-cassette in the final CRI-SPA step, we reasoned that the largest source of error would be contributed by white unmodified recipient cells, which were not eliminated during CRI-SPA steps 2–3, and which would reduce the yellowness score of the mutant colony. To explore the extent of this potential problem, we picked cells from each of the four colonies obtained for each strain on the final CRI-SPA screen plate (YPD-G418) and replica spotted approximately 1000 (two of the colonies) and 10 000 cells (the other two colonies) on YPD + G418 (control) and YPD + G418 + NTC plates, see Supplementary Figure S8. Contaminating unmodified YKO cells should form white colonies on YPD + G418, but not on YPD + G418 + NTC medium. For 360 of the strains (96%), sufficient viable cells were transferred (> 100 colonies produced in three of the four trials) to allow for further analysis. Next, we qualitatively compared the number of colonies formed on YPD + G418 and YPD + G418 + NTC plates for all trials. This analysis identified eight strains (∼2% of the 360 strains), which in one or more trials contained >1% unmodified YKO library cells in the colonies of the final CRI-SPA screening plate. Most of these colonies contained little if any modified YKO cells and these trials therefore represent rare flawed CRI-SPA transfer trials. We note that for six of these eight strains ≥ two of the four trials were not contaminated with unmodified YKO cells. Accordingly, we conclude that transfer of unmodified YKO cells through the CRI-SPA procedure is a minor problem.

We also reasoned that yellow CD-Btx and diploid cells, which survived CRI-SPA steps 4–5, would compromise strain-to-strfain betaxanthin production comparisons if they were present in large amounts in the CRI-SPA colonies. We therefore examined the final CRI-SPA plates for colonies that contained CD-Btx and were diploid. In parallel with the experiment presented above, cells were therefore spotted in the same way, but on solid SC-Ura medium, which allows CD-Btx and diploid cells to grow, and on solid SC-Ura + G418 medium, which allows diploid, but not CD-Btx, cells to grow. These analyses demonstrated that basically no CD-Btx cells survive the CRI-SPA screen. Some diploid cells survive, but they constitute less than 0.1% of the cells in the colonies. For a few strains, one of the four trials contained ∼1% diploid cells. Hence, we conclude that carryover of CD-Btx and diploid cells to the final CRI-SPA plate constitutes a minor problem in this CRI-SPA experiment. Lastly, we note that the *scp160*Δ strain, which produced a false phenotype in the *ade2*Δ*::hphNT1* cassette transfer experiment failed to produce more than a few surviving cells after CRI-SPA in this experiment.

### Introduction of the betaxanthin pathway into a genome-wide library of gene-deletion strains

With the CRI-SPA based betaxanthin-pathway transfer system and a betaxanthin quantification method in place, we then used CD-Btx to transfer the Btx-cassette to the 4787 unique deletion strains of the YKO library using CRI-SPA in quadruplicate amounting to a total volume of 19148 transfer events. To address positional effects known to affect colony size in high density array screens (36,37), we randomized the YKO library arrays (in 384 format) when scaling up to 1536 quadruplicate format. Specifically, each of the four 384 arrays from a given library plate were pinned on a different screening plate (see Supplementary Figure S2B and Materials and Methods for full description of the image analysis, colony randomization and data processing pipeline).

In the first set of experiments, we employed NTC selection in the final step of CRI-SPA as a safety measure to ensure that all cells contain the Btx-cassette. Encouragingly, the vast majority of the colonies appeared yellow after CRI-SPA indicating that the CRI-SPA mediated transfer of the betaxanthin pathway into the library was successful (Figure 3C). We extracted the yellowness score of all colonies and after removing remaining positional artifacts and outliers (see MATERIALS AND METHODS including accompanying python scripts available on our CRI-SPA repository), 4224 strains (≈ 88% of the library) produced ≥ three viable CRI-SPA colonies and were henceforth included in our analysis.

We finally ranked the mean yellowness for all genes (Figure 3D) and found that the screen produced a strong yellowness signal to noise ratio (the *Z*-scores of the 16 top and bottom hits was 40.83, –6.93; respectively.). This analysis demonstrated that our screen can produce strains with a large range of betaxanthin synthesis abilities.

Since CRI-SPA colonies are not monoclonal, we isolated three candidates representing three different color levels and restreaked them on solid YPD medium with appropriate selection. As expected, the individual streaks of cells formed colonies that were uniformly yellow indicating little, if any, presence of contaminating cell types. Importantly, the color differences between the sets were easily distinguished on plate images, validating the position of the mutant strains in the color ranking scheme (Figure 3D).

### Screen to screen reproducibility and marker-free CRI-SPA

**Figure 4.**
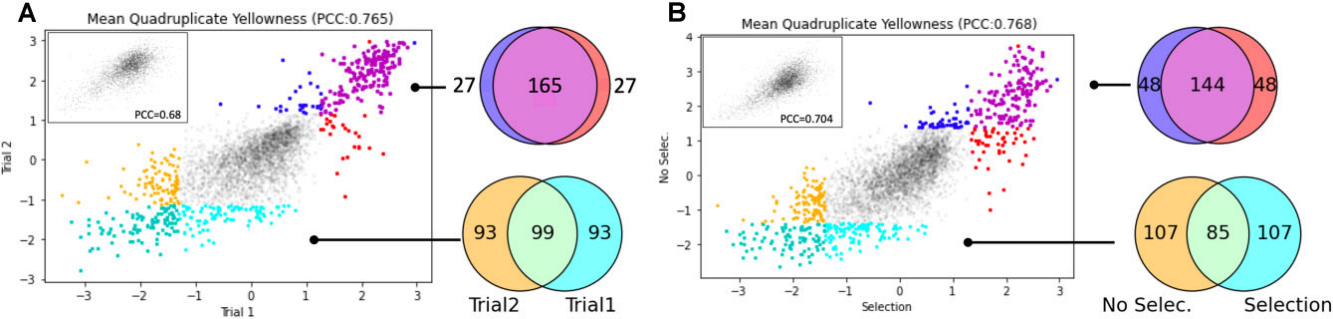
Reproducibility of betaxanthin CRI-SPA experiments in the presence and absence of pathway transfer selection. (**A**) The mean yellow color intensities obtained for each mutant in repeat 1 and repeat 2, which both were performed in the presence of *natMX* to select for the Btx-cassette genes. (**B**) The mean yellow color intensities obtained for each mutant in trial 1 and trial 3. Trial 3 was performed in the absence of *natMX*. The top and bottom 192 genes and their overlap across screens are colored and displayed in flanking Venn diagrams. Insets show the correlation for colony fitness across screens.

Confident that the screen readout was accurate for a handful of strains, we repeated the entire CRI-SPA experiment to validate its reproducibility. After CRI-SPA, the yellowness of the individual mutants obtained in this experiment, trial 2, were then plotted against the yellowness obtained for the mutants in the first experiment, trial 1. The clear linear correlation between the two trials (PCC = 0.765) demonstrates the reproducibility of CRI-SPA (Figure 4A). Moreover, we found that, from all successfully labeled genes in the two screens, 86% (*P* = 1.09e–220) and 52% (*P* = 9.97e–83) of the 192-top and 192-bottom hits were common in the two trials, respectively. Hence, and especially for the top-hits, reproducibility was high.

In a third experiment, trial 3, we explored the possibility of transferring the five-gene Btx-cassette from CD-Btx to the full YKO library without selecting for the *natMX* marker, which is present in the Btx-cassette. In this screen, the CRI-SPA plasmid's restriction of the target site maintained the sole selection for genetic transfer. With this set-up, 95.1% of mutants produced at least three colonies after CRI-SPA mediated transfer of the Btx-cassette. Importantly, the yellowness score in this trial had a clear linear correlation with trial 1 (PCC = 0.768, Figure 4B). Moreover, when the 192 most yellow and 192 least yellow mutants obtained in the two trials were compared, the top and bottom groups had 75% (*P* ≤ 10e–180) and 44% (*P* ≤ 10e–69) overlap across screens, respectively (Figure 4B). Hence, the above analyses demonstrate that CRI-SPA is reproducible and that the combined effect of a mutation and a genetic feature can be reproduced even without selecting for this feature.

### Systems perspective of betaxanthin production

**Figure 5:**
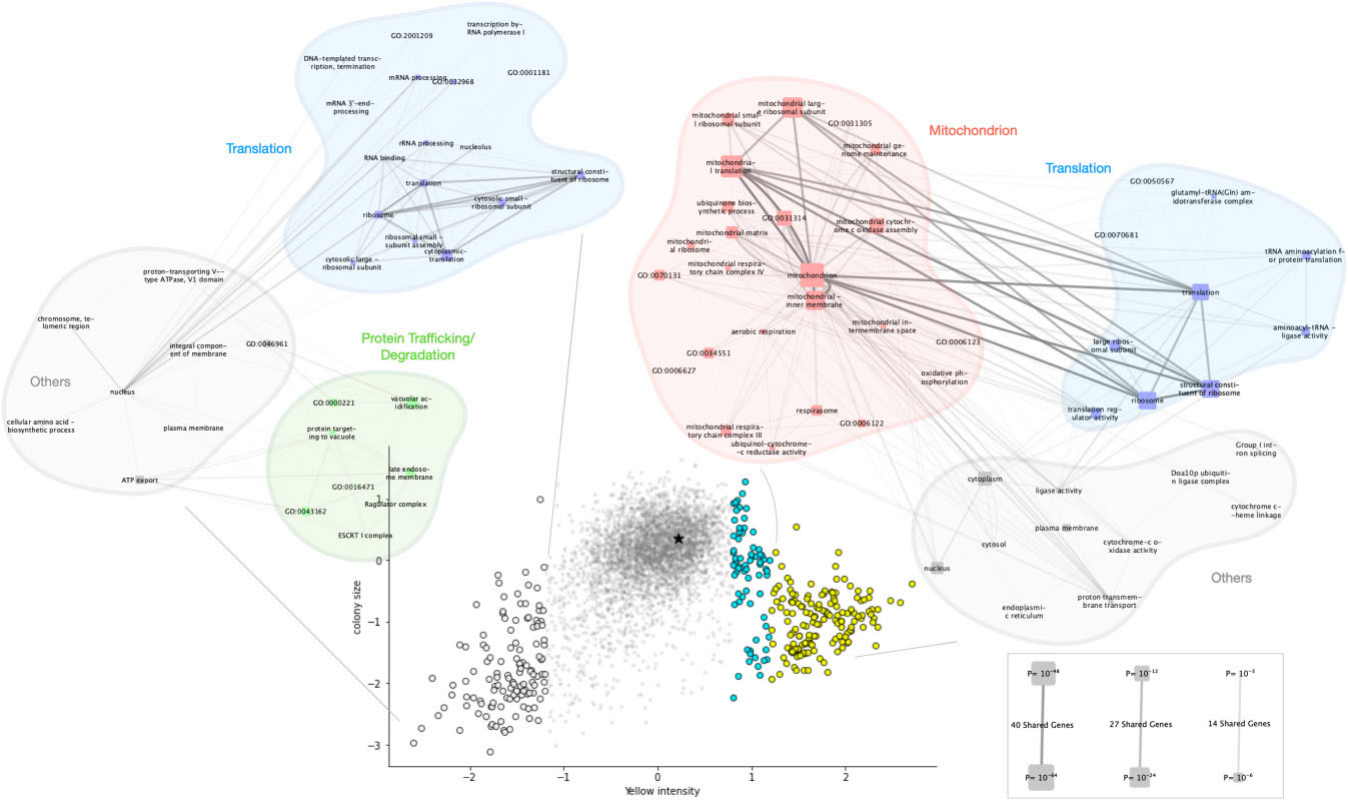
Betaxanthin yield and fitness relationship reveals mechanistic patterns in betaxanthin production. Colony size (i.e. fitness) vs Yellow Color intensity (i.e. Betaxanthin Yield). The results of Gene Ontology Enrichment Analyses on several subgroups of genes are shown as graphs for the bottom hits (white, 1.2 std below the screen mean) and top hits (yellow, 1.2 std above the screen mean). As indicated by the bottom right scale, node size indicates the significance of GO terms enrichment, edge transparency indicates the number of shared genes between GO terms. The cyan group did not show any term enrichment. The position of the BY-Btx strain is marked with a black asterisk.

In contrast to one-pot screens, CRI-SPA produces a read-out for each individual strain within a library. We investigated whether this systematic gene labeling could reveal new system level patterns in betaxanthin production. For this analysis, we combined the data of four screens (see supplementary part), obtaining a CRI-SPA score for 4761 genes out of the 4788 present in our library with a median of 15 replicates (16 is maximum) per gene. We first plotted yellowness of all strains against colony size using the latter feature as a proxy for fitness, see Figure 5. Plotted in this way, the vast majority of cells map in the close vicinity of the WT, indicating that the mutations in these strains do not impact betaxanthin production (Figure 5). We next grouped the remaining mutant strains according to their yellowness score: the ‘yellow’ group was defined as mutants with the highest level of betaxanthin production (1.2 standard deviation greater than the screen mean, yellow points in Figure 5). The ‘white’ group was defined as mutants with the lowest level of betaxanthin production (1.2 standard deviation below the screen mean, white points in Figure 5). We observed that most members of the yellow group displayed a strong fitness penalty. We therefore defined a third group of mutants, the ‘cyan group’, see Figure 5. This group contains mostly highly yellow strains with little or no fitness penalty. Next, we examined whether specific cellular functions could be linked to betaxanthin levels by running gene ontology enrichment analyses (38) within the three groups. The cyan group did not produce any enrichment. This may not be surprising as the hits in this group were the least reproducible (Supplementary Figure S9). The white group, despite its broad fitness range, was enriched for terms describing the degradation machinery, translation, and vacuolar mechanisms. Most strikingly, the yellow group displayed a dramatic enrichment for genes associated with mitochondrial functions, particularly the respiratory chain, which might explain the fitness penalty observed in this group. Hence, this analysis demonstrates that CRI-SPA is able to identify a novel network of genes, which impact functions of a specific organelle in the cell, and which, when deleted, lead to reduced fitness and high betaxanthin levels.

### Validation of CRI-SPA hits in liquid culture

**Figure 6.**
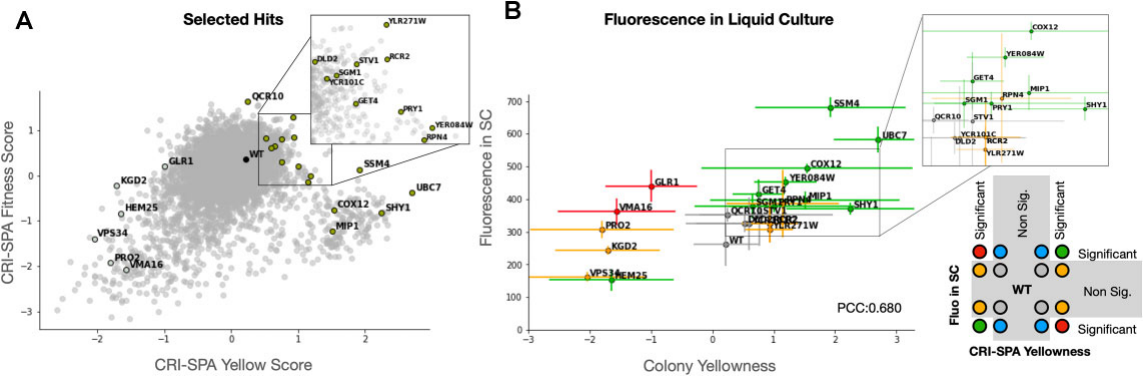
CRI-SPA hits identified on solid medium produce enhanced amounts of betaxanthin in liquid medium. (**A**) selected Btx-hit strains yellow and fitness scores within the screen. (**B**) Correlation between CRI-SPA yellowness score and fluorescence of reversed engineered hits after 7 days cultivation in liquid SC medium. Error bars show standard deviations for 16 and 3 replicates for CRI-SPA and liquid Fluorescence, respectively. Markers coloring scheme, below inset, is defined by the agreement between CRI-SPA score and fluorescence in liquid medium, and significance difference from WT reported by a one-sided Welch's test.

Currently, CRI-SPA screens for phenotypic changes on solid medium and it is possible that a phenotypic hit may potentially vanish in different growth conditions. To explore that possibility, we decided to test how selected Btx-hit strains fare in liquid SC-medium. Moreover, to further validate that strains picked up in a CRI-SPA screen truly reflect the impact of the specific mutation provided by the YKO library strain, we re-introduced the mutations in the BY-Btx reference strain by conventional one-step gene-targeting (57) using *kanMX* to select for the deletion. Hence, we reintroduced 16 top and 6 bottom KO hits in the reference strain BY-Btx, covering a variety of cellular functions and strain fitnesses (Figure 6A, Supplementary Table S4). To avoid penalizing slow growing strains, and strains with a prolonged lag-phase, we grew the reference and the 22 mutant BY-Btx strains in liquid SC medium with 2% glucose for 7 days. This setup is possible as betaxanthin does not appear to be catabolized during fermentation as its levels remained stable throughout the stationary phase of a BY-Btx pilot experiment (Supplementary Figure S7).

We then examined all strains for betaxanthin production using a fluorescence readout at the fermentation end-point. The results showed that betaxanthin levels strongly correlated with the yellowness score obtained for the same mutants on the final CRI-SPA selection plate (PCC = 0.680, see Figure 6B). For the positive hits, of the 13 scoring significantly higher than the WT for yellowness in the CRI-SPA screen, nine were significantly more fluorescent than the reference BY-Btx strain, indicating that they also produced significantly increased levels of betaxanthin in liquid SC medium. Indeed, the two most yellow mutant strains identified in the CRI-SPA screen, *ubc7*Δ and *ssm4*Δ/*doa10*Δ, were 2.6-fold and 2.2-fold more fluorescent than BY-Btx (*ssm4*Δ/*doa10*Δ: *P* ≤ 6 × 10e–4, *ubc7*Δ: *P* ≤ 2 × 10e–3), respectively. Another set of positive CRI-SPA Betaxanthin hits were composed by *mip1*Δ, shy*1*Δ, *YER084W*Δ, and *cox12*Δ. The corresponding genes all encode mitochondrial proteins showing that dysfunctional mitochondria may also stimulate betaxanthin production in liquid SC medium. For the negative hits, the *hem25*Δ hit was the only strain displaying significantly reduced fluorescence compared to BY-Btx (0.59-fold, *P* ≤ 0.05). Taken together, we show that CRI-SPA is able to identify gene hits, which perform in growth conditions routinely used for metabolic engineering strain development.

## DISCUSSION

We have developed a highly efficient, low cost and rapid method, CRI-SPA, for parallelized transfer of a genetic feature from a haploid CD strain to any haploid yeast library strain, which is *ura3*. In this report, we have focused on gene transfer into the YKO library, but we note that other libraries like the GFP library (57) and SWAT libraries (15,16) could also be used in CRI-SPA experiments. At the individual level, CRI-SPA mediated gene transfer is highly efficient and can, unlike SGA, be performed without selecting for the genetic feature of interest. At the global level, the efficiency of transfer is also very high and only 62 genes (1.3%) of the library consistently failed to produce any colonies through three screen repeats. As expected, these genes are associated with mating, cell division, and DNA repair, which represent functions that are essential for CRI-SPA (Supplementary Table S7). To this end, we stress that CRI-SPA mediated gene transfer is based on interchromosomal gene conversion. Hence, the donor sequence persists in the diploid state where gene transfer takes place allowing for a long tunable window of opportunity for transfer. This contrasts the scenario in classical gene targeting where the genetic feature is introduced via a linear DNA donor fragment or a self-cleaving plasmid system delivered by a SPA based method (16,58–59). In those cases, the time window where gene transfer can take place is limited to the lifespan of the donor fragment.

We note that CRI-SPA colonies are not clones and that the colonies may contain other cell types than modified recipient cells. Hence CRI-SPA colonies may potentially contain escapers, unmodified recipient-, CD- and diploid cells on the final selection plate. In the betaxanthin experiment when applying selection for the Btx-cassette, these cell types were present in amounts lower than 1% and did not influence the readout significantly. In the corresponding experiment where we did not apply selection for the Btx-cassette, we observed a few cases where large amounts of unmodified recipient strains survived step 2 where diploids are selected. These cases were typically flawed CRI-SPA transfer trials, and we envision that they may form if unusually high amounts of recipient cells are pinned from the starting library plate to the plate where mating takes place. If at the same time, mating is poor, diploid cells may not outcompete unmodified recipient strain on the plate selecting for diploids despite their advantage of selection. We stress that all four trials are rarely tainted by contaminating cells, and if two rounds of CRI-SPA are performed, each with quadruplicate trials, we believe that a recipient library can be reliably covered for ≥99% of the strains. The exceptions will be if the genetic feature of interest introduces a strong fitness disadvantage as this will likely increase the number of undesired other cell types in the CRI-SPA colonies. In these cases, the selection scheme may need further optimization. In the pilot *ade2*Δ:*:hphNT1* transfer experiments, where introduction of this mutation comes with a fitness penalty, we added additional adenine to the medium to reduce this effect. Nevertheless, a few colonies were still contaminated by white cells to artificially impact the colony color phenotype. Some of these cells turned out to be diploid recipient YKO cells where only one of the homologous chromosomes had received the *ade2*Δ::*hphNT1* cassette. Although it may be difficult to avoid occasional formation of diploid recipient cells due to endoreduplication, the effect of these cells on the colony phenotype will be minimized if both chromosomes receive the genetic feature of interest. This can be achieved by optimizing the gRNA to ensure that all target chromosomes are cut in all cells. Consequently, we recommend verifying Cas9-sgRNA cutting efficiency with TAPE, and we have previously observed fold-differences in transformant numbers in the presence and absence of a repair template that exceed 400 (46). Another way to ensure that both sister chromatids of the recipient strain will receive the modification is to prolong the window of opportunity for Cas9 cleavage. This can easily be achieved by extending the duration of the raffinose step where little cell growth takes place. Another source of contaminating cells could in principle be aneuploid cells containing CD chromosomes where the *Kl_URA3* markers were mutated. The forward mutation rate of endogenous *URA3* selecting for 5-FOA resistance is around 5 × 10^−8^ (60). Assuming that the forward mutation rate of *Kl_URA3* markers are similar to the rate of *URA3*, we find the presence of such cells highly unlikely. Lastly, we note that the betaxanthin phenotype of the hit strains may be caused by other accidental genetic alterations in the recipient strain, which potentially could have been introduced during construction of the gene deletion or during the CRI-SPA procedure. In our Btx-Cassette transfer experiment, we successfully demonstrated that Btx-hit strains maintained their phenotype if they were recreated *de novo* by conventional gene targeting, showing that the phenotype of the hit strain was caused by the specific mutation of the YKO library strain (Figure 6).

In principle, CRI-SPA can be used as a tool to forward the understanding of any biological process in yeast as it allows a genetic feature to be combined with large libraries of other mutations. In this report, we demonstrate in a pilot experiment that CRI-SPA can be used to identify epistatic interactions in a genetic network. Specifically, we identified the epistatic interaction between ade2 and ade3 in a set of other mutant strains (Figure 2). More thoroughly, we demonstrated how CRI-SPA can be used to develop a metabolic-engineering strategy for production of the plant metabolite betaxanthin by identifying target genes that can be manipulated to increase betaxanthin production. This was achieved by using CRI-SPA to transfer a five-genes cassette for betaxanthin production into the YKO library. Our successful transfer of the Btx-cassette to close to all of the strains of the YKO library strains allowed us to provide a systems overview of functions that influence betaxanthin production in yeast. Importantly, our CRI-SPA screen identified genes that are strongly enriched for GO-terms forming functional networks supporting a mechanistic consensus across hits. For example, our analyses showed that deletion of genes, *UBC7* and *SSM4/DOA10*, involved in ubiquitin mediated endoplasmic-reticulum-associated protein degradation, ERAD (61), benefit betaxanthin production. In this context it is important to note that the betaxanthin pathway includes a p450 enzyme, CYP76AD1, which is N-terminally linked to the ER membrane. P450s are notoriously difficult to produce in fungal production hosts and it is tempting to speculate that folding of CYP76AD1 in yeast is inefficient and therefore prone to ERAD. In this model, increased betaxanthin levels are the result of the higher CYP76AD1 activity that will materialize in the absence of functional ERAD. From a gene-network perspective, we note that Ubc7 is a ubiquitin conjugating enzyme, which is C-terminally tail-anchored to the ER membrane (62). This fact may explain why *get4*Δ is also scored as a positive hit as Get4 is part of the GET complex that integrates C-terminal-tail-anchored proteins into the ER membrane (63). In another part of the gene network connected by CYP76AD1 we find genes encoding mitochondrial proteins. For example, deletion of *HEM25* negatively impacts betaxanthin production. Hem25 is a mitochondrial transporter facilitating the import of glycine, a precursor for the biosynthesis of heme. Since heme is an essential cofactor of CYP76AD1 (14,64); it is easy to envision that reduced heme availability might impair the activity of CYP76AD1, and in turn betaxanthin production. From another point of view, we also stress that our CRI-SPA screen, in addition to well-characterized genes, was also able to pick up Btx-hit genes with no assigned function. Such gene hits would be difficult, if not impossible, to identify via traditional metabolic-model based hit-finding strategies.

Since our betaxanthin CRI-SPA screen was designed to facilitate future betaxanthin cell factory development, it was important to validate that our CRI-SPA betaxanthin hits identified on solid medium would maintain their phenotype if they were propagated in liquid medium, which is most often used in production. In favor of using CRI-SPA as a valuable starting point in the development of a metabolic engineering strategy, most of the recreated betaxanthin hits influenced betaxanthin production in a manner similar to the original effects observed for the corresponding CRI-SPA colonies. Altogether, CRI-SPA was able to unravel novel physiology impacting betaxanthin production and to identify gene targets that can be used to develop a metabolic engineering strategy for production of betaxanthin, and potentially of L-DOPA derived compounds. For the present, and for other metabolic engineering projects, we envision that by examining gene-interaction networks identified by a CRI-SPA screen, it will be possible to identify mutations that can be advantageously combined. In particular, mutations that are not part of the same functional network may be combined to provide additive, or even synergistic, effects on production of the desired product. We note that not only the positive hits can be used in a future strategy, negative hits may point to limiting functions, which may be improved by overexpressing the hit gene.

Our successful marker-free gene-transfer experiment opens promising avenues for CRI-SPA. Bypassing the need for marker selection sets the stage for the transfer of point mutations as well as the multiplex transfer of several genetic features located at different positions in the genome at once. In another application, CRI-SPA could be used to transfer a genetic trait to libraries with different genetic backgrounds or even closely related species, as long as they can mate with the CD. This is possible as CRI-SPA is independent of meiotic recombination and sporulation. Currently, a limitation of our method is that a recipient library needs to be *ura3* to be compatible with the CRI-SPA selection procedure. This could be bypassed by replacing the *Kl_URA3* markers in the CD strain by another counter-selectable marker like *amdS* (65) to produce an even more flexible version of CRI-SPA.

To conclude, we have demonstrated that CRI-SPA is a powerful high-throughput data generation tool opening up a range of new exciting applications both in fundamental genetics and in genome engineering.

## Supplementary Material

gkad656_supplemental_files

## Data Availability

The top hits, bottom hits and failing genes lists are available as Supplementary Tables 5, 6 and 7, respectively. A sample of 13 screen images, the raw datasets obtained from such images for each screen, the individual and the corrected + filtered datasets are all available on our CRI-SPA github repository (https://github.com/pc2912/CRI-SPA_repo). All scripts used in the analysis and for the drawing of the figures of this work are also available on the CRI-SPA github repository (permanent doi: https://doi.org/10.5281/zenodo.8160760).
