# Supplementary material for: CRI-SPA: a high-throughput method for systematic genetic editing of yeast libraries
Source: Nucleic Acids Res. 2023 Aug 12;51(17):e91. doi: 10.1093/nar/gkad656 (PMC10516668; doi:10.1093/nar/gkad656)
Supplement: gkad656_supplemental_files [file gkad656_supplemental_files.zip › Supp Methods S1.pdf]

Supplementary Text for :

CRI-SPA – a high-throughput method for systematic genetic editing of yeast libraries

Paul Cachera*, Helén Olsson*, Hilde Coumou*, Mads L. Jensen, Benjamín J. Sánchez, Tomas Strucko, Marcel van den Broek, Jean-Marc Daran, Michael K. Jensen, Nikolaus Sonnenschein, Michael Lisby, Uffe H. Mortensen.

Plasmid construction and PCR.

DNA assembly was done by USER-cloning (1,2) employing uracil-specific excision reagent (USER™) enzyme from New England Biolabs. DNA polymerases were purchased from Thermo Scientific and used according to the supplier's instructions. Amplification of DNA for USER cloning was carried out using Phusion U Hot Start DNA Polymerase (Thermo Fisher). Standard PCR amplifications for cloning were run using Phusion Hot Start II High-Fidelity DNA Polymerase. In the case of colony PCR, Taq DNA Polymerase was used (New England Biolabs). Purification of DNA fragments obtained by PCR or from agarose gel bands was done using the illustra GFX PCR DNA and Gel Band Purification Kit (GE Lifesciences).

Plasmid pHO8 harbors a *cas9* (*Streptococcus pyogenes*)::*LEU2* gene-targeting substrate for integration into chromosomal integration site X-3 (3). The pHO8 was made by amplifying *cas9* from plasmid pCfB2312 (4) with primers HOP89 and HOP90 (see Supplementary Table S3, which contains all primers used in this study). The fragment was then inserted into a linear vector fragment of the integrative plasmid pCfB257 (5) obtained by PCR using primers HOP91 and HOP92.

The integrative plasmid, pBTX1, for inserting betaxanthin-synthesizing genes into the XII-5 site (3) was constructed using the following parts: The gRNA cassette *SNR52p-gRNA.XII-5-SUP4t* and the NTC resistance *AgTEFp-natMX-AgTEFt* were amplified from pCfB3050 (6). The *ARO7*^{G141S}-*TEF1p-PGK1p-ARO4*^{K229L} cassette was amplified from pXI-2-ARO4*-ARO7* plasmid. The BvCYP76AD1 cassette (2663 bp), including the *TPI1* promoter region (583 bp) and bidirectional *TDH3* terminator region (580 bp) and an MjDOD cassette (1520 bp), including the *CCW12* promoter region (700 bp) were synthesized by GenScript (USA). The USER cloning cassette and the inner flanks of XII-5 were amplified from pCfB261 (5). All fragments were assembled using USER fusion. pBTX2 was built from pBTX1 excluding the NTC marker with PCR with primers PR_DIV2288 and ANT_P494 and ligated into pCfB2909 with USER assembly.

sgRNA- and CRI-SPA Vectors: For construction of the CRI-SPA vector pHO-ADE2, targeting the *ADE2* locus, the target sequence in pCfB3050 (6) was excluded by linearizing the plasmid with uracil-containing primers HOP60 and HOP61 and replaced by an insert containing the 20 bp *ADE2* targeting sequence. The insert was obtained by annealing two single stranded oligonucleotides HOP190 and HOP191. For insertion of an additional *Kl_URA3* (*Kluyveromyces lactis URA3*) marker between *ADE2* and the telomere, an sgRNA plasmid for targeting an intergenic sequence downstream of the *ADE2* locus (pHO22) was constructed as described above but instead annealing the two oligonucleotides HOP173 and HOP174. Similarly, the sgRNA plasmid for targeting the intergenic sequence downstream the XII-5 integration site (pHO25), was produced by inserting a 20 bp targeting sequence obtained by annealing the two oligonucleotides HOP183 and HOP184. pHO-XII-5, expressing an sgRNA targeting XII-5, was made by exchanging by USER cloning the *natMX* marker cassette of plasmid pCfB3050 (6) for a

hphNT1 cassette PCR amplified from pMEL12 (7) using uracil-containing primers HOP217 and HOP218.

All plasmids maps are provided as '.gbk' files in the online supplementary material.

References

1- F. Geu-Flores, H. H. Nour-Eldin, M. T. Nielsen, and B. A. Halkier. User fusion: a rapid and efficient method for simultaneous fusion and cloning of multiple pcr products. Nucleic acids research, 35(7):e55, 2007

2- H. H. Nour-Eldin, B. G. Hansen, M. H. Nørholm, J. K. Jensen, and B. A. Halkier. Advancing uracil-excision based cloning towards an ideal technique for cloning pcr fragments. Nucleic acids research, 34(18):e122–e122, 2006.

3- M. D. Mikkelsen, L. D. Buron, B. Salomonsen, C. E. Olsen, B. G. Hansen, U. H. Mortensen, and B. A. Halkier. Microbial production of indolylglucosinolate through engineering of a multi-gene pathway in a versatile yeast expression platform. Metabolic Engineering, 14(2):104–111, 2012.

4- V. Stovicek, I. Borodina, and J. Forster. CRISPR–cas system enables fast and simple genome editing of industrial *Saccharomyces cerevisiae* strains. Metabolic Engineering Communications, 2:13–22, 2015

5- N. B. Jensen, T. Strucko, K. R. Kildegaard, F. David, J. Maury, U. H. Mortensen, J. Forster, J. Nielsen, and I. Borodina. Easyclone: method for iterative chromosomal integration of multiple genes *Saccharomyces cerevisiae.* FEMS yeast research, 14(2):238–248, 2014.

6- M. M. Jessop-Fabre, T. Jakoˇcı̄unas, V. Stovicek, Z. Dai, M. K. Jensen, J. D. Keasling, and I. Borodina. Easyclone-markerfree: A vector toolkit for markerless integration of genes into *Saccharomyces cerevisiae* via CRISPR-Cas9. Biotechnology Journal, 11(8):1110–1117, 2016

7- R. Mans, H. M. van Rossum, M. Wijsman, A. Backx, N. G. Kuijpers, M. van den Broek, P. Daran-Lapujade, J. T. Pronk, A. J. van Maris, and J.M. G. Daran. Crispr/cas9: a molecular swiss army knife for simultaneous introduction of multiple genetic modifications in Saccharomyces cerevisiae. FEMS Reast Research, 15(2), 2015
