# Supplementary material for: CRI-SPA: a high-throughput method for systematic genetic editing of yeast libraries
Source: Nucleic Acids Res. 2023 Aug 12;51(17):e91. doi: 10.1093/nar/gkad656 (PMC10516668; doi:10.1093/nar/gkad656)
Supplement: gkad656_supplemental_files [file gkad656_supplemental_files.zip › Supp Methods S2.pdf]

SUPPLEMENTARY METHODS S2:

CRI-SPA – a high-throughput method for systematic genetic editing of yeast libraries

Paul Cachera*, Helén Olsson*, Hilde Coumou*, Mads L. Jensen, Benjamín J. Sánchez, Tomas Strucko, Marcel van den Broek, Jean-Marc Daran, Michael K. Jensen, Nikolaus Sonnenschein, Michael Lisby, Uffe H. Mortensen.

CRI-SPA: A Method that allows Arrayed Transfer of a Genetic Feature from a Single Donor Strain to Individual Strains of a Library

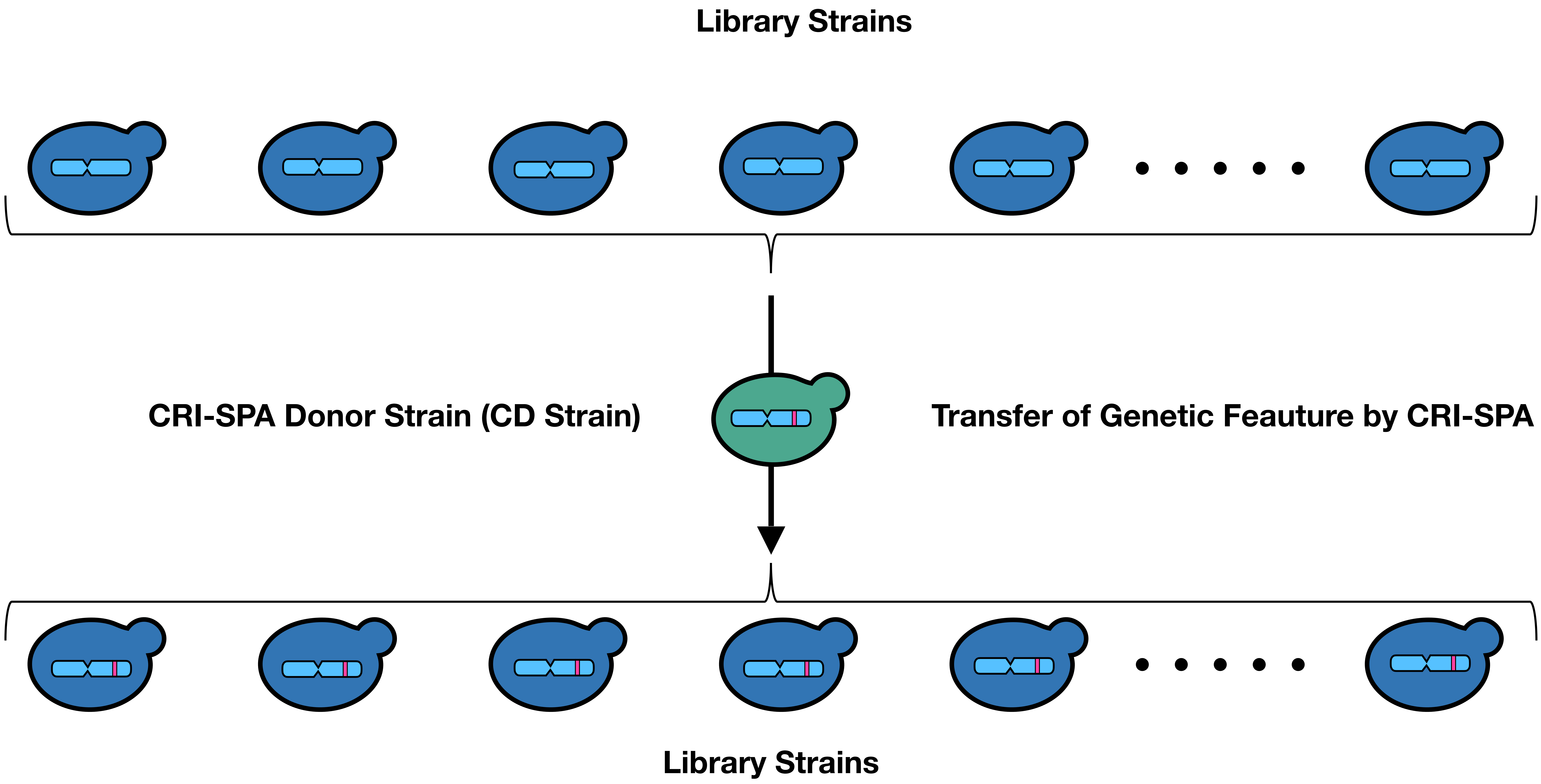

Prerequisites for a CRI-SPA Experiment

CD Strain

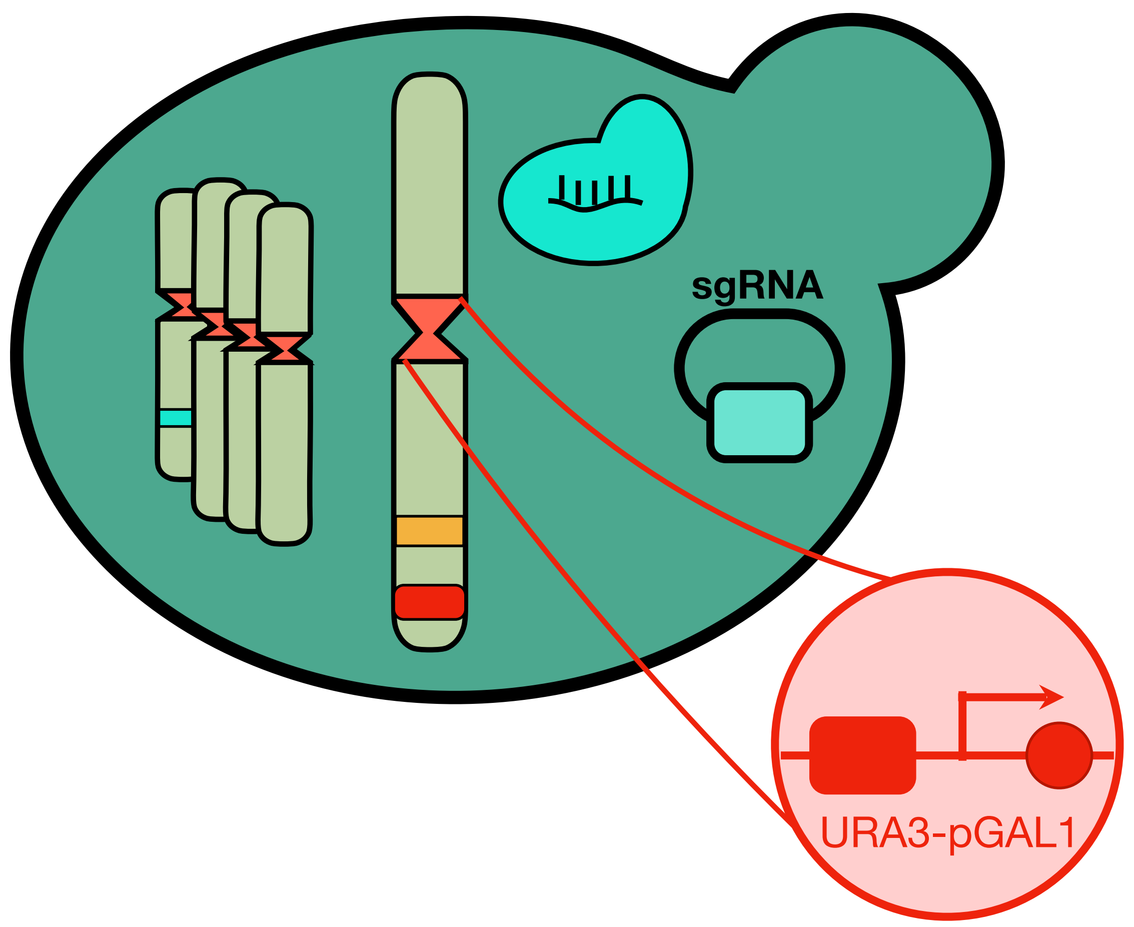

Library Strains

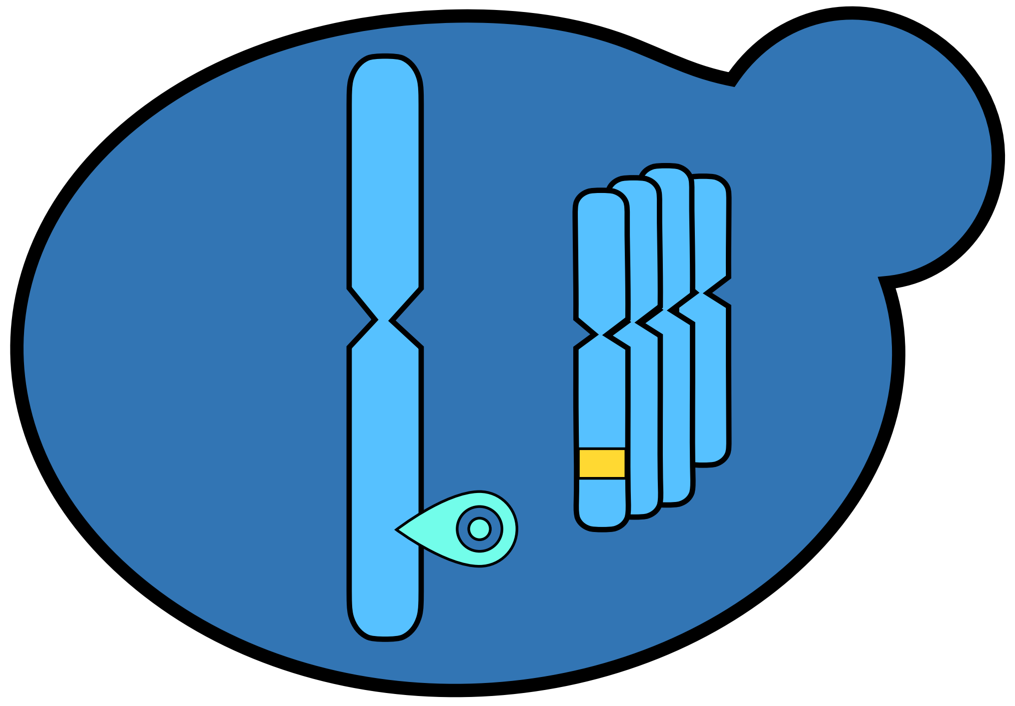

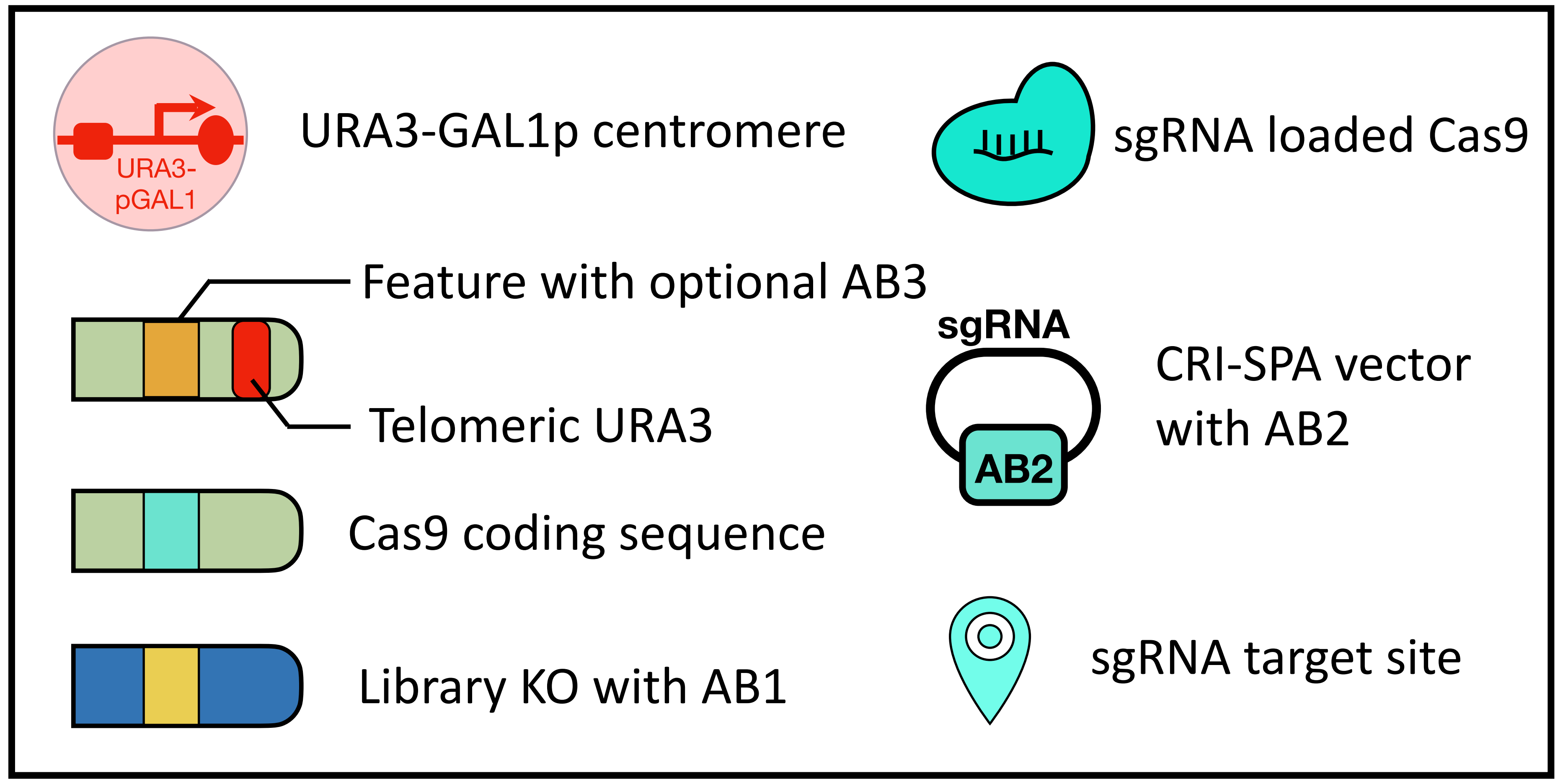

A CRI-SPA Donor Strain with a Genetic Feature of interest

The CD strain is constructed by CRISPR technology. For details, see Supplementary Figure S1.

No Cas9/gRNA action in the CD Strain. The target site of the CRISPR nuclease has been destroyed by insertion of the genetic feature of interest.

A Yeast Strain Library

Strains need to:
1)be ura3
2)be of opposite mating type of the CD strain
3)contain a selectable marker with a function absent in the CD Strain

CRI-SPA Procedure Step 1: Mating CD Strains to Library Strains

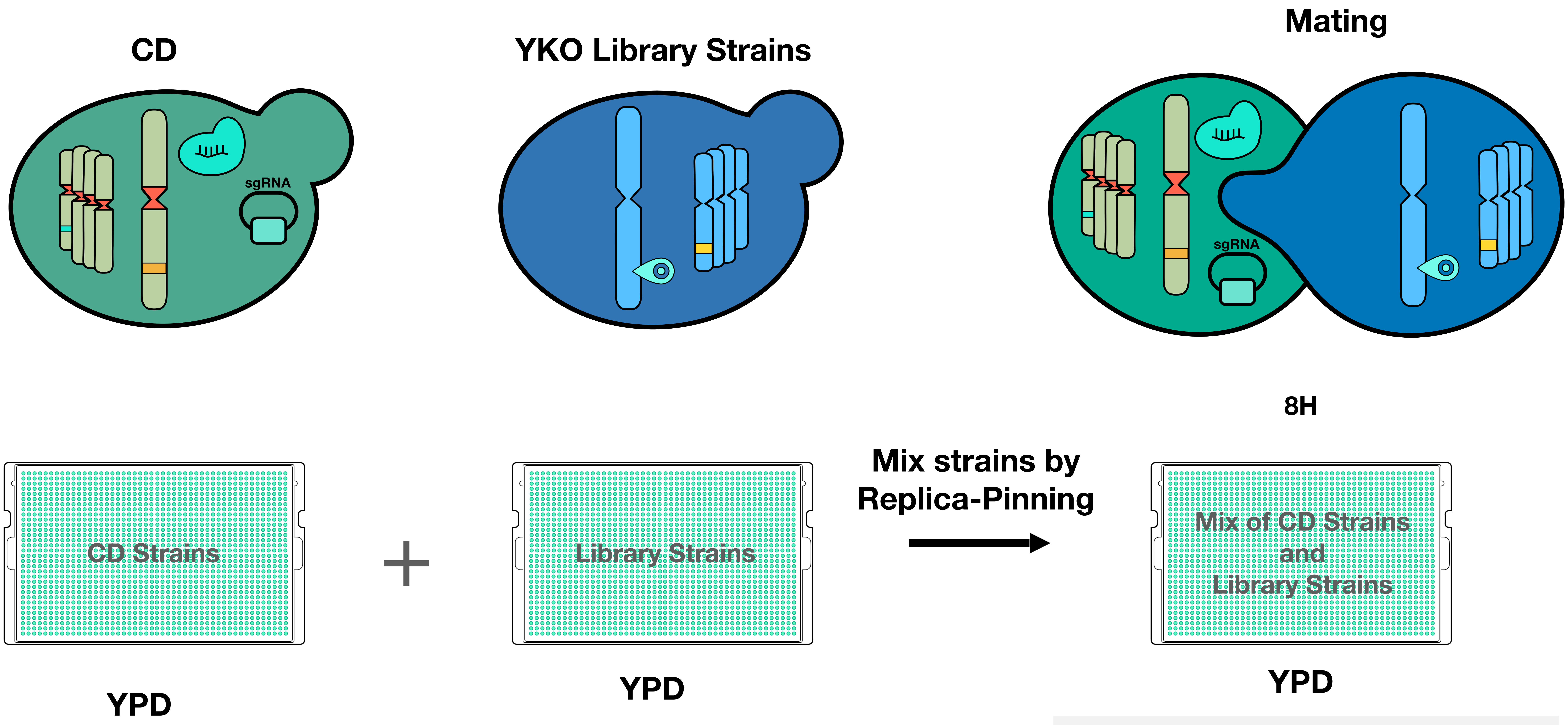

Growth of both strain types on the YPD plates sets the stage for mating on every pinned position

CRI-SPA Procedure Step 2: Feature Transfer

Mating

Transfer of Genetic Feature

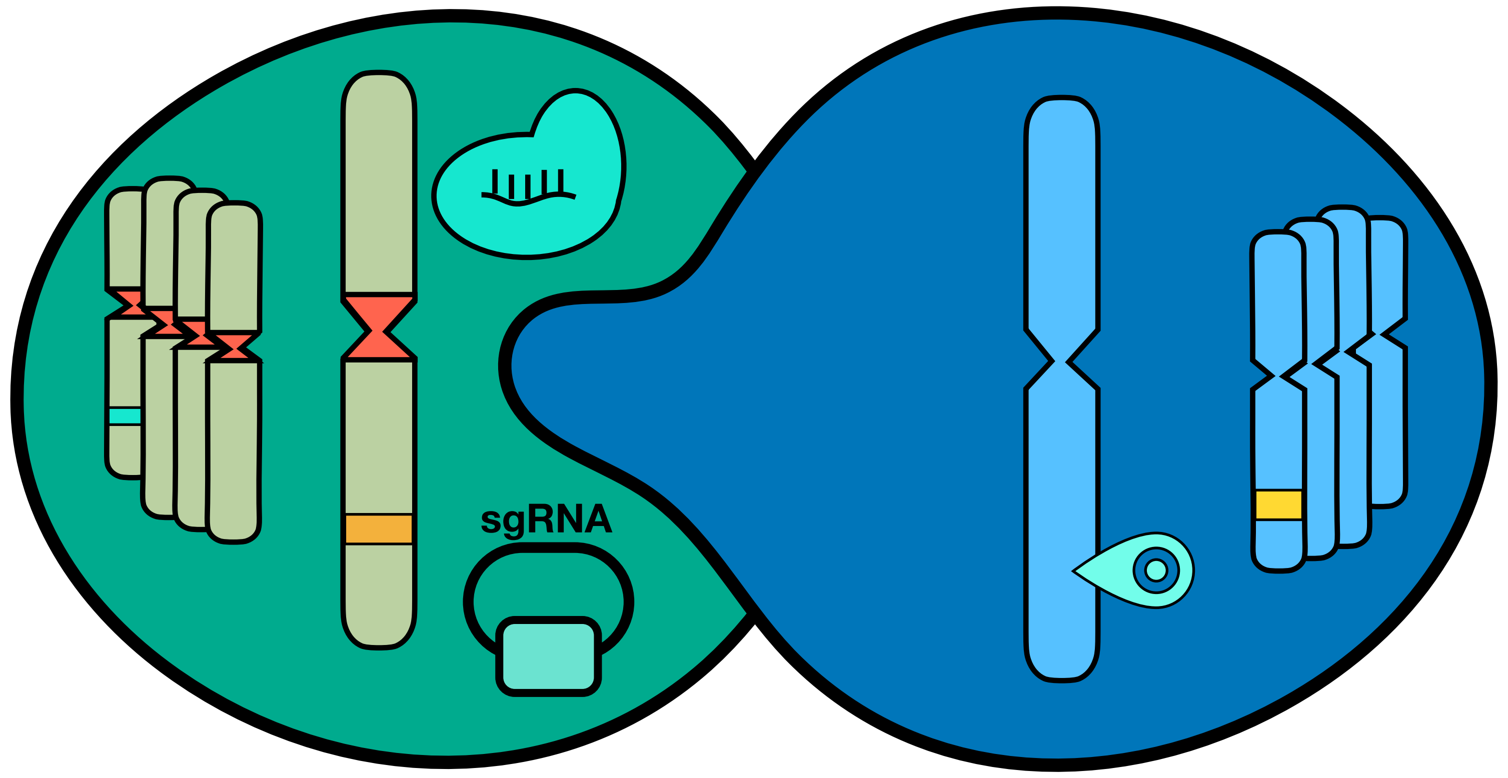

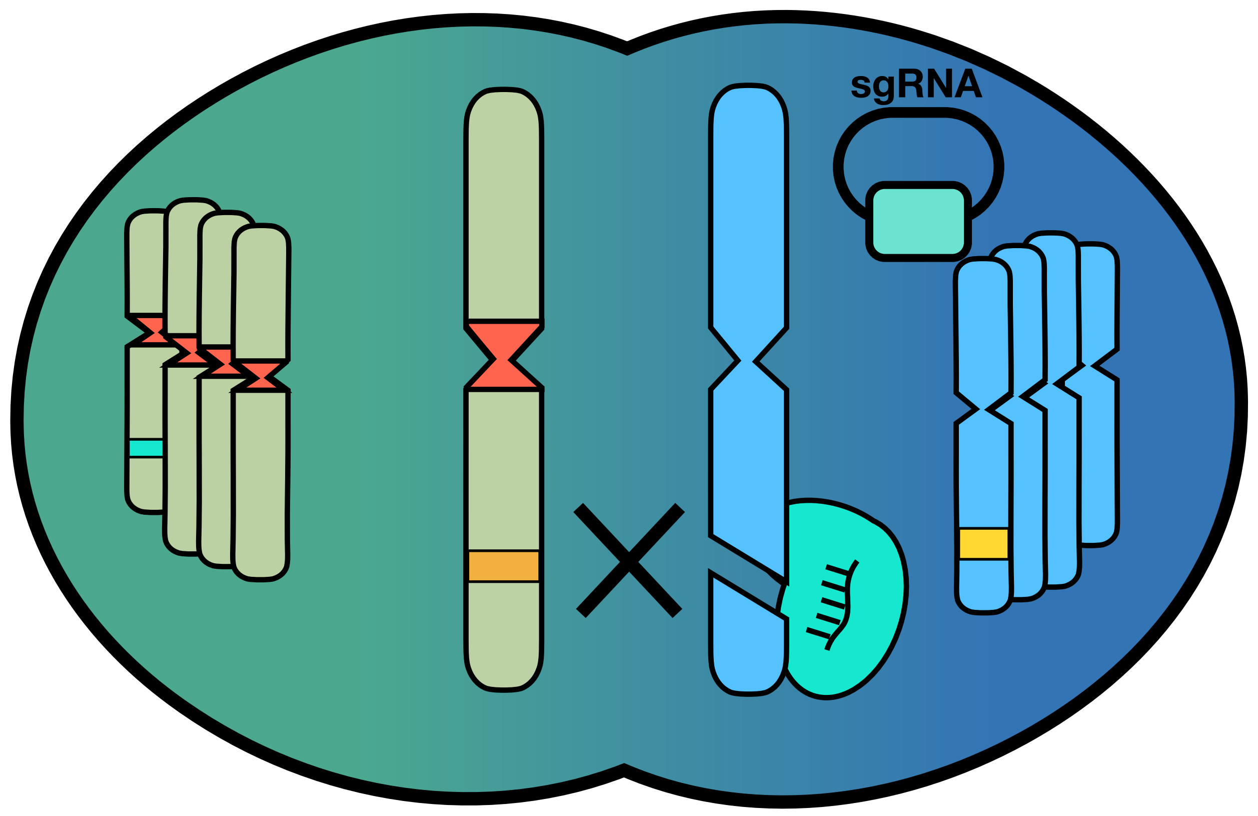

Actions in the Diploid:
1)Cas9/gRNA cuts the unmodified locus in the library chromosome
2)Repair of the resulting DNA DSB by homologous recombination using the modified chromosome in the CD strain as template
3)Transfer of genetic feature from donor to library chromosome by gene conversion

8H

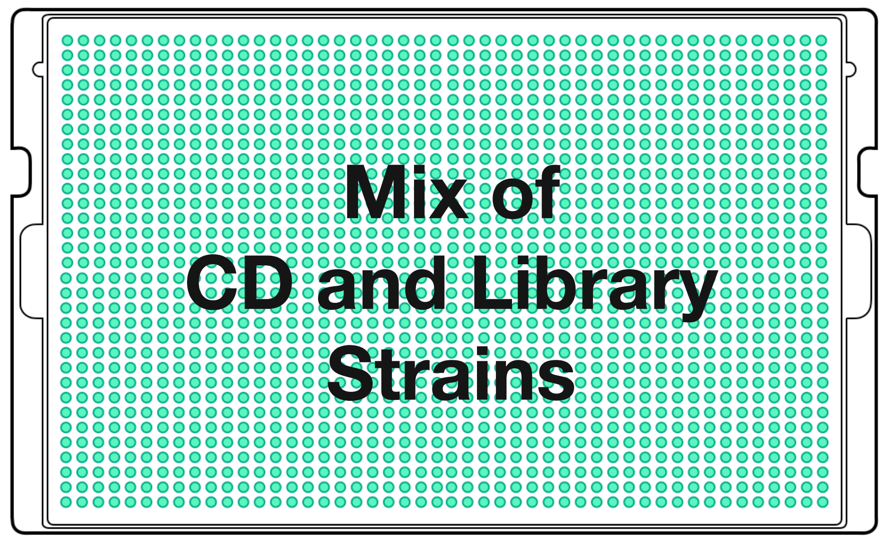

YPD (Glucose)

24-48H

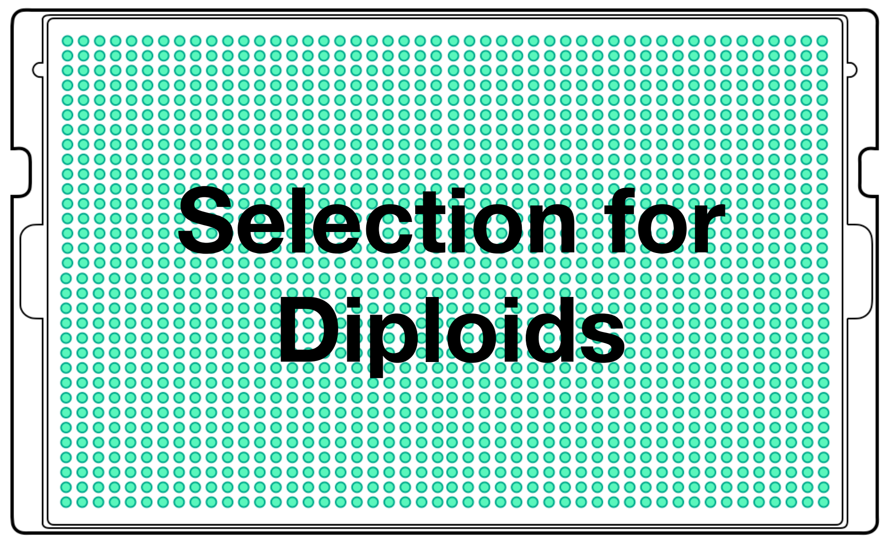

YPR (Raffinose)
AB1+2

Selection for Diploids and CRISPER vector:
1) Haploid CD cells contain the AB2 resistance gene harbored on the CRISPR vector
2) Haploid library cells contain the AB1 resistance gene
3) Diploid cells contain the A1 and A2 resistance genes

The presence of AB1 and AB2 in the medium kills un-mated CD- and library strains, respectively. Only diploid cells survive as they contain both resistance genes.

CRI-SPA Procedure Step 3 & 4: Haploidization

Diploid after Transfer of the Genetic Feature of Interest

Haploidization - Part 1

Haploidization – Part 2

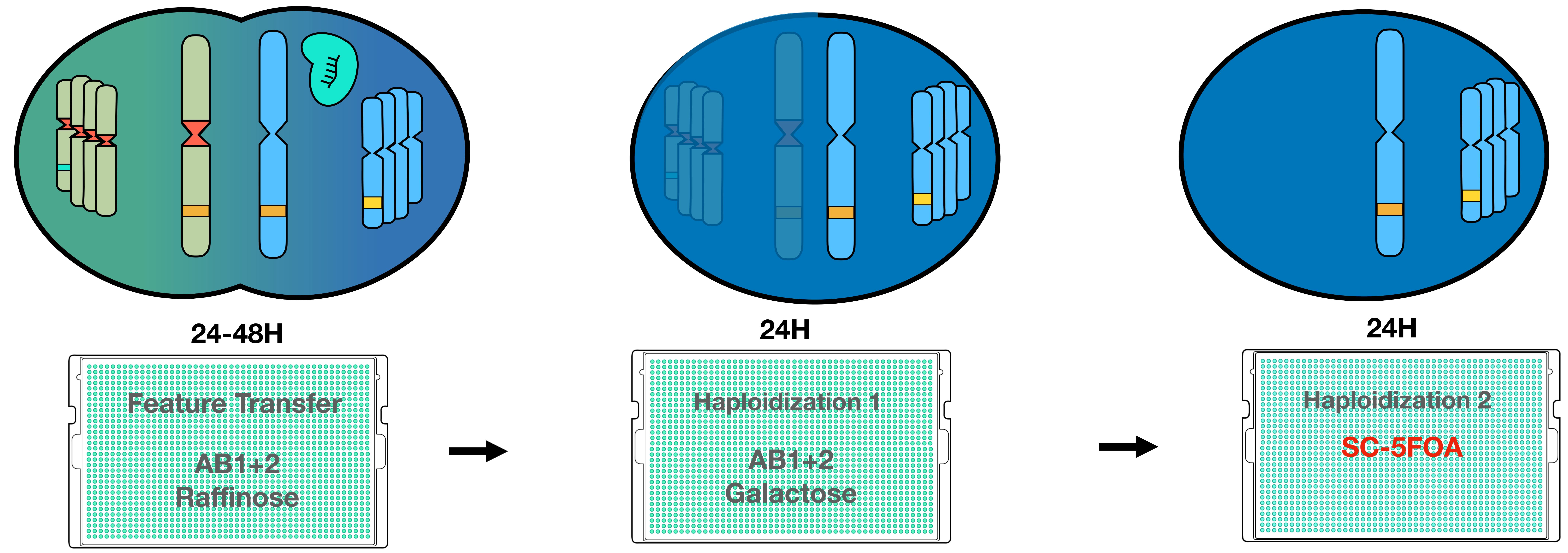

No Cas9/gRNA action in the Diploid Strain
After successful transfer of the genetic feature of interest. The target site of the CRISPR nuclease in the library cells has been destroyed by insertion of the genetic feature of interest.

Haploidization
1)Activated Gal promoters destroys centromeric functions of in all CD strain chromosomes
2)CD strain chromosomes faill attachemtn to mitotic spindle during cell diviaion
3)Chromsomes that are not attached to the mitotic spindle will be lost during cell divisions
4) Spontaneous loss of for the CRISPR vector as it is not selected for

Diploid and partially haploidized cells are eliminated on SC-FOA medium.
1)URA3 encodes Orotidine 5'-phosphate decarboxylase, which is required for converting 5-FOA into toxic metabolites
2)Cells containing one or more CD chromosomes will die as they contain at least one Kl_URA3 gene
3)Only Cells that are fully haploidized and contain solely library chromosomes survives as they do not have Kl_URA3

CRI-SPA Procedure Step 5: Final Selection

Haploidization – Part 2

Final Selection

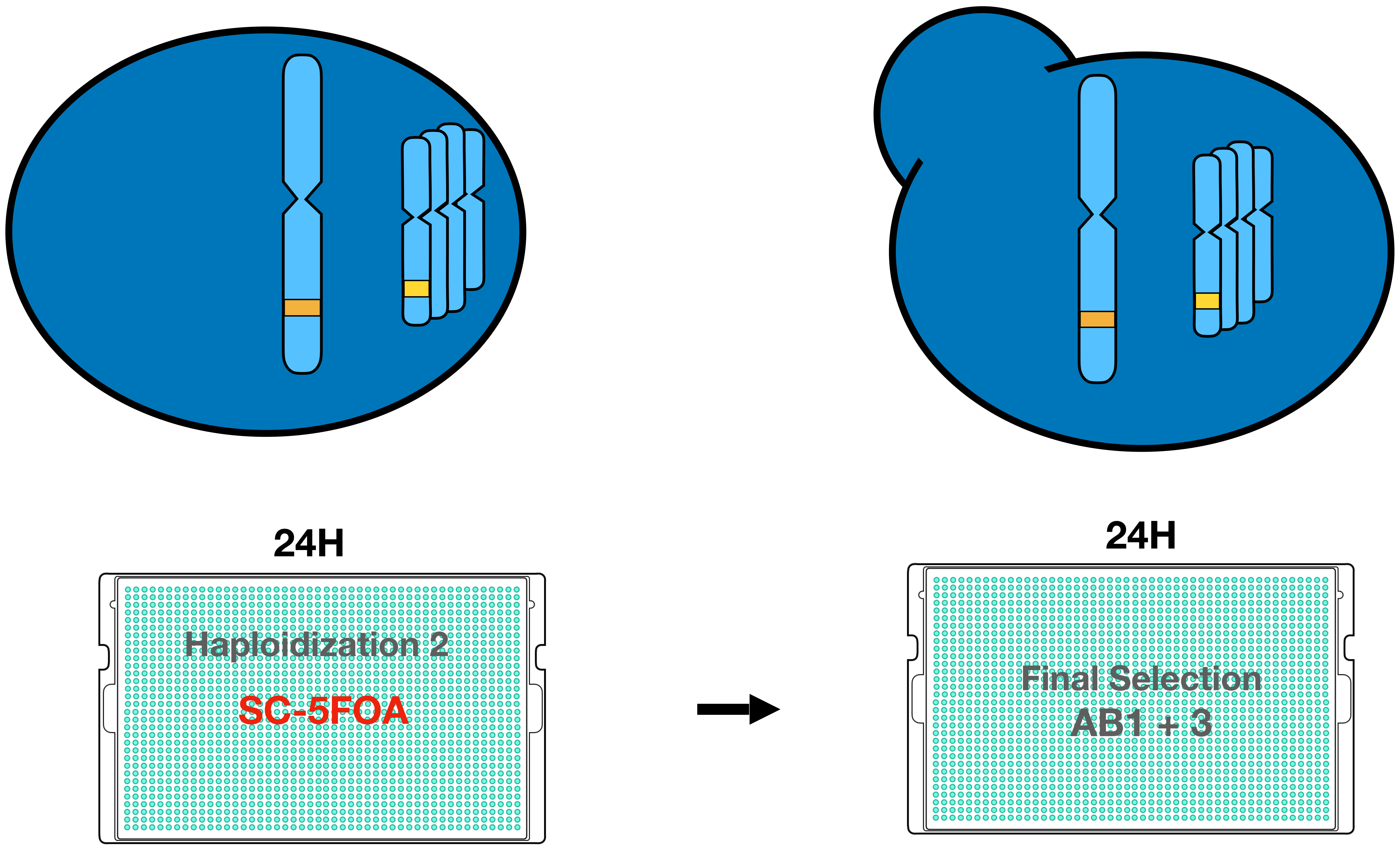

Formation of library cells containing the genetic feature of interest in the desired locus

1)Selection for AB1 eliminates any carry-over of donor strains.
2)Optional: Selection for AB3 eliminates any carry-over of unmodified library strains
3)Only modified library cells survive as they contain resistance markers AB1 and AB3
