# Supplementary material for: CRI-SPA: a high-throughput method for systematic genetic editing of yeast libraries
Source: Nucleic Acids Res. 2023 Aug 12;51(17):e91. doi: 10.1093/nar/gkad656 (PMC10516668; doi:10.1093/nar/gkad656)
Supplement: gkad656_supplemental_files [file gkad656_supplemental_files.zip › Supplementary Figure S8.pptx]

## Slide 1
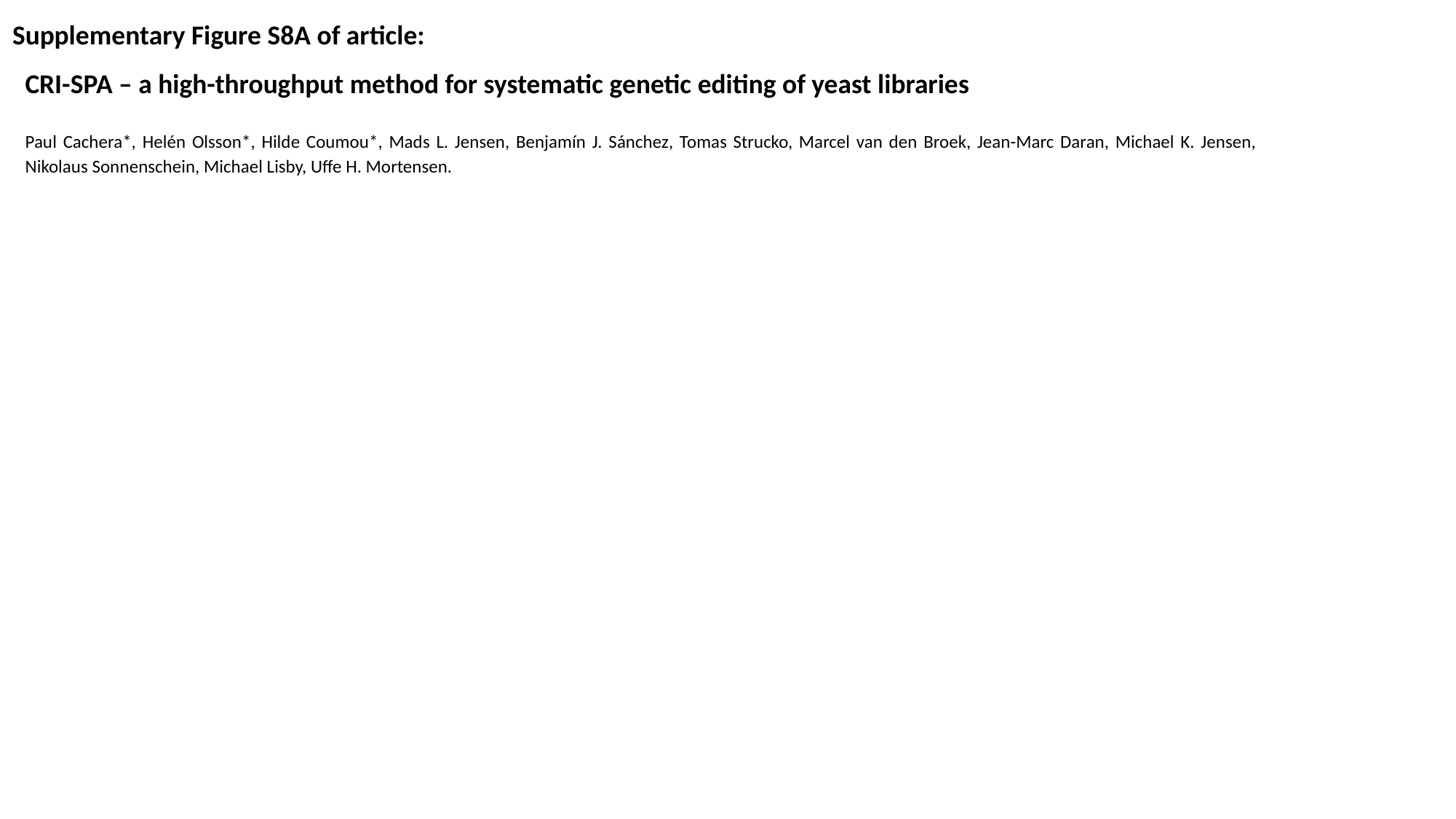

Supplementary Figure S8A of article:
CRI-SPA – a high-throughput method for systematic genetic editing of yeast libraries
Paul Cachera*, Helén Olsson*, Hilde Coumou*, Mads L. Jensen, Benjamín J. Sánchez, Tomas Strucko, Marcel van den Broek, Jean-Marc Daran, Michael K. Jensen, Nikolaus Sonnenschein, Michael Lisby, Uffe H. Mortensen.

## Slide 2
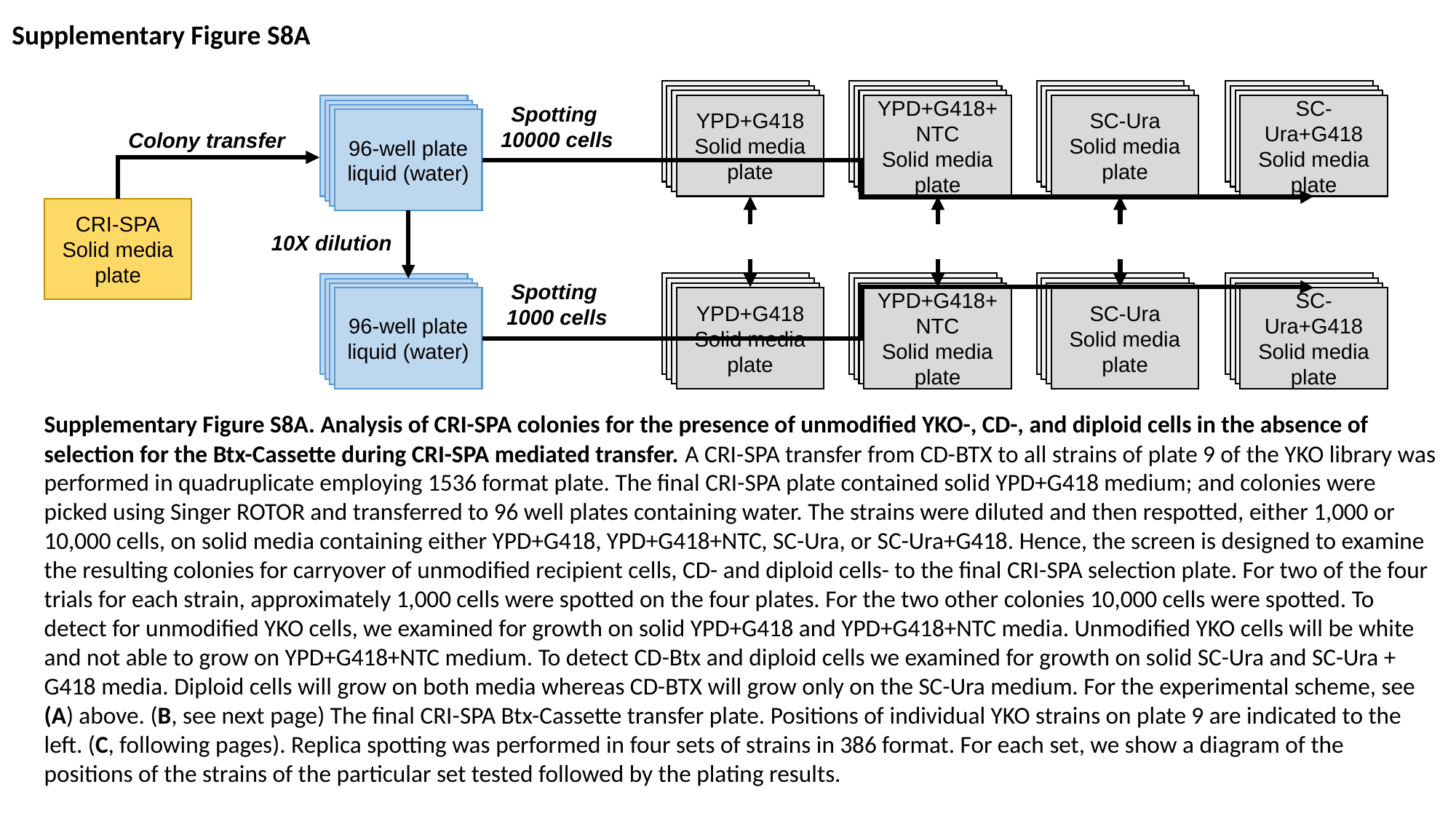

Supplementary Figure S8A
YPD+G418
Solid media plate
YPD+G418+NTC
Solid media plate
SC-Ura
Solid media plate
SC-Ura+G418 Solid media plate
Spotting
10000 cells
96-well plate liquid (water)
Colony transfer
CRI-SPA
Solid media plate
10X dilution
Spotting
1000 cells
96-well plate liquid (water)
YPD+G418
Solid media plate
YPD+G418+NTC
Solid media plate
SC-Ura
Solid media plate
SC-Ura+G418 Solid media plate
Supplementary Figure S8A. Analysis of CRI-SPA colonies for the presence of unmodified YKO-, CD-, and diploid cells in the absence of selection for the Btx-Cassette during CRI-SPA mediated transfer. A CRI-SPA transfer from CD-BTX to all strains of plate 9 of the YKO library was performed in quadruplicate employing 1536 format plate. The final CRI-SPA plate contained solid YPD+G418 medium; and colonies were picked using Singer ROTOR and transferred to 96 well plates containing water. The strains were diluted and then respotted, either 1,000 or 10,000 cells, on solid media containing either YPD+G418, YPD+G418+NTC, SC-Ura, or SC-Ura+G418. Hence, the screen is designed to examine the resulting colonies for carryover of unmodified recipient cells, CD- and diploid cells- to the final CRI-SPA selection plate. For two of the four trials for each strain, approximately 1,000 cells were spotted on the four plates. For the two other colonies 10,000 cells were spotted. To detect for unmodified YKO cells, we examined for growth on solid YPD+G418 and YPD+G418+NTC media. Unmodified YKO cells will be white and not able to grow on YPD+G418+NTC medium. To detect CD-Btx and diploid cells we examined for growth on solid SC-Ura and SC-Ura + G418 media. Diploid cells will grow on both media whereas CD-BTX will grow only on the SC-Ura medium. For the experimental scheme, see (A) above. (B, see next page) The final CRI-SPA Btx-Cassette transfer plate. Positions of individual YKO strains on plate 9 are indicated to the left. (C, following pages). Replica spotting was performed in four sets of strains in 386 format. For each set, we show a diagram of the positions of the strains of the particular set tested followed by the plating results.

## Slide 3
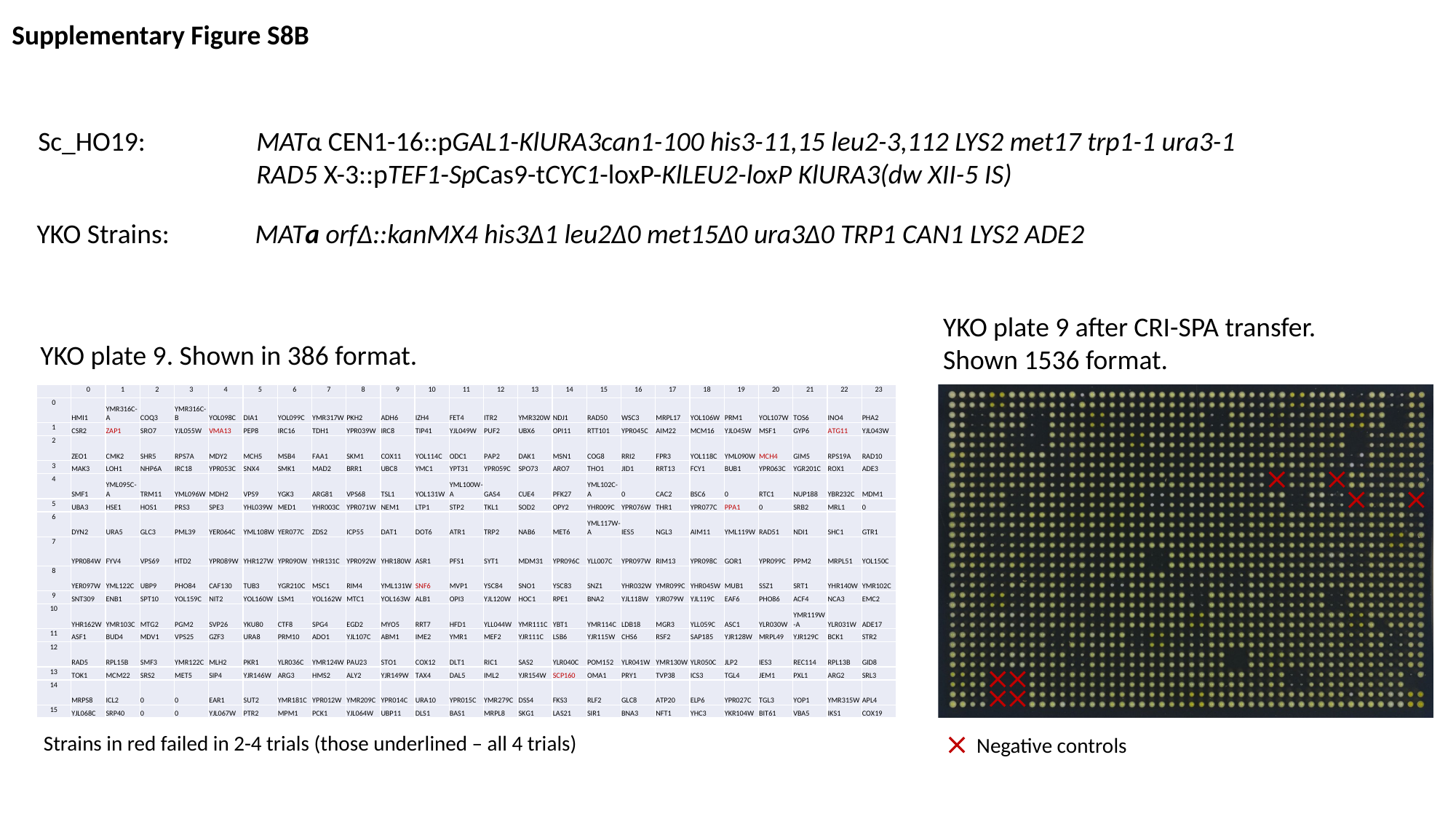

Supplementary Figure S8B
Sc_HO19: 	MATα CEN1-16::pGAL1-KlURA3can1-100 his3-11,15 leu2-3,112 LYS2 met17 trp1-1 ura3-1
		RAD5 X-3::pTEF1-SpCas9-tCYC1-loxP-KlLEU2-loxP KlURA3(dw XII-5 IS)
YKO Strains:	MATa orfΔ::kanMX4 his3Δ1 leu2Δ0 met15Δ0 ura3Δ0 TRP1 CAN1 LYS2 ADE2
YKO plate 9 after CRI-SPA transfer.
Shown 1536 format.
YKO plate 9. Shown in 386 format.
Strains in red failed in 2-4 trials (those underlined – all 4 trials)
Negative controls

## Slide 4
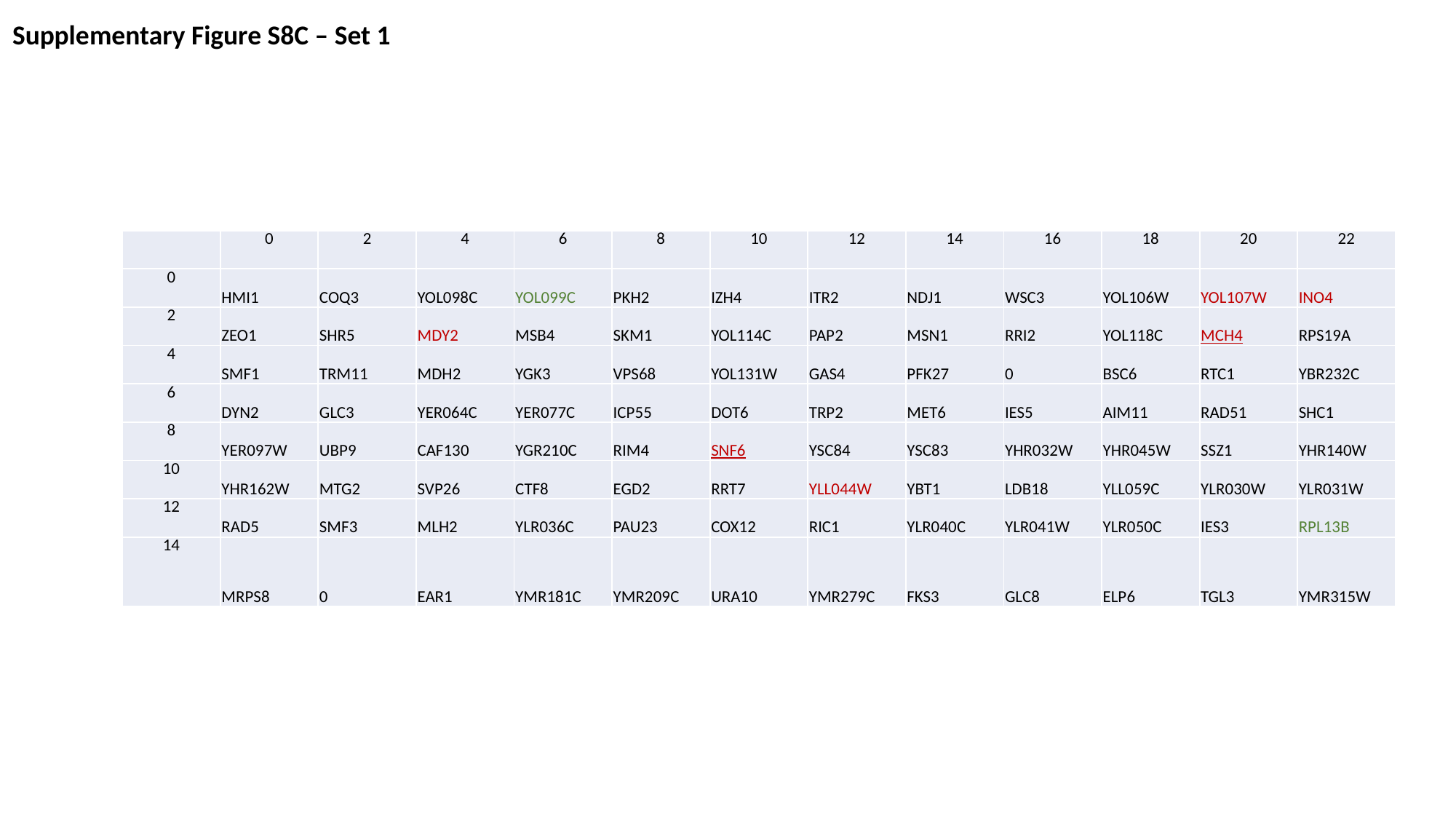

Supplementary Figure S8C – Set 1
| | 0 | 2 | 4 | 6 | 8 | 10 | 12 | 14 | 16 | 18 | 20 | 22 |
| --- | --- | --- | --- | --- | --- | --- | --- | --- | --- | --- | --- | --- |
| 0 | HMI1 | COQ3 | YOL098C | YOL099C | PKH2 | IZH4 | ITR2 | NDJ1 | WSC3 | YOL106W | YOL107W | INO4 |
| 2 | ZEO1 | SHR5 | MDY2 | MSB4 | SKM1 | YOL114C | PAP2 | MSN1 | RRI2 | YOL118C | MCH4 | RPS19A |
| 4 | SMF1 | TRM11 | MDH2 | YGK3 | VPS68 | YOL131W | GAS4 | PFK27 | 0 | BSC6 | RTC1 | YBR232C |
| 6 | DYN2 | GLC3 | YER064C | YER077C | ICP55 | DOT6 | TRP2 | MET6 | IES5 | AIM11 | RAD51 | SHC1 |
| 8 | YER097W | UBP9 | CAF130 | YGR210C | RIM4 | SNF6 | YSC84 | YSC83 | YHR032W | YHR045W | SSZ1 | YHR140W |
| 10 | YHR162W | MTG2 | SVP26 | CTF8 | EGD2 | RRT7 | YLL044W | YBT1 | LDB18 | YLL059C | YLR030W | YLR031W |
| 12 | RAD5 | SMF3 | MLH2 | YLR036C | PAU23 | COX12 | RIC1 | YLR040C | YLR041W | YLR050C | IES3 | RPL13B |
| 14 | MRPS8 | 0 | EAR1 | YMR181C | YMR209C | URA10 | YMR279C | FKS3 | GLC8 | ELP6 | TGL3 | YMR315W |

## Slide 5
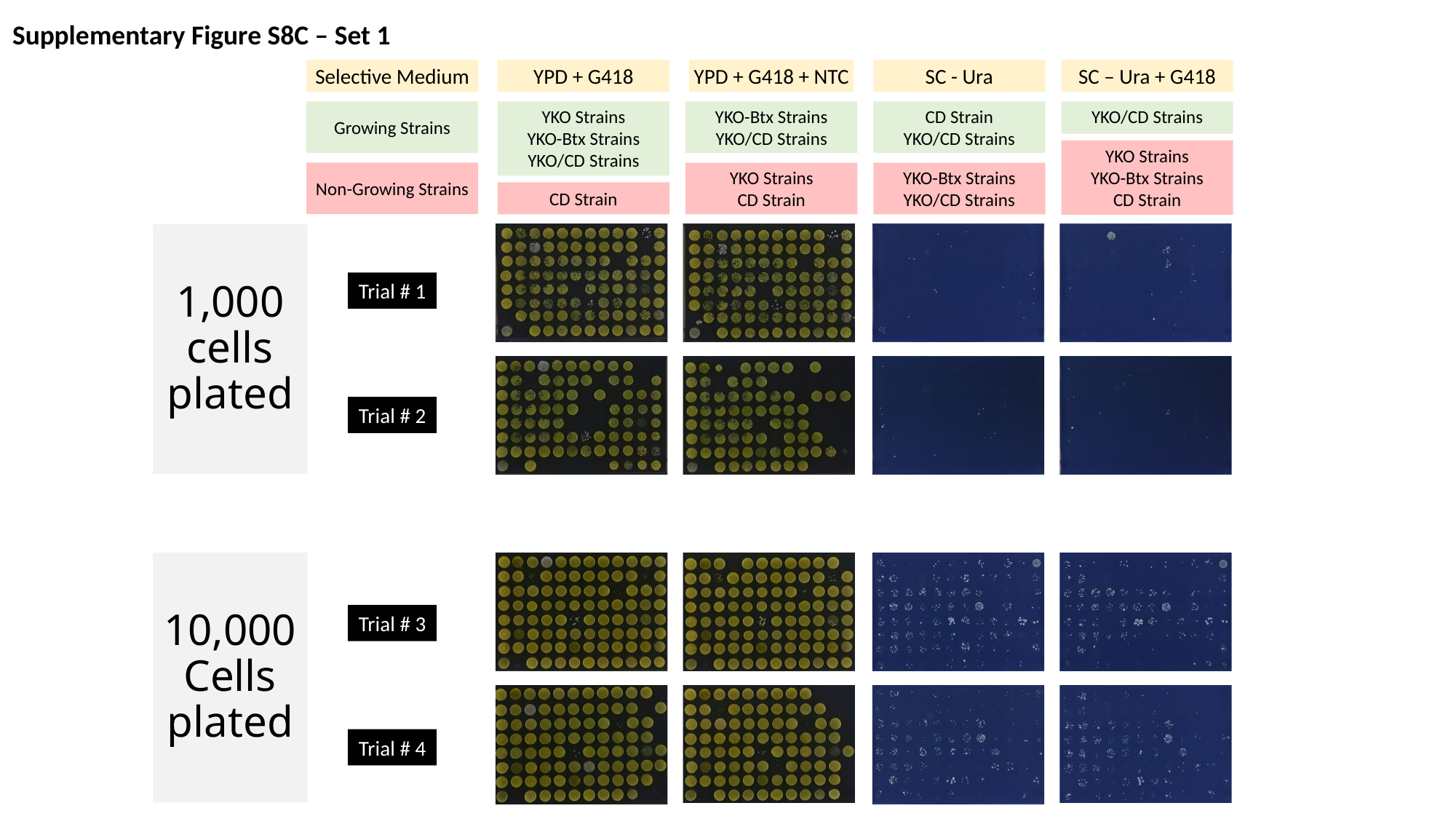

Supplementary Figure S8C – Set 1
Selective Medium
Growing Strains
Non-Growing Strains
Trial # 1
Trial # 2
Trial # 3
Trial # 4
YPD + G418
YKO Strains
YKO-Btx Strains
YKO/CD Strains
CD Strain
YPD + G418 + NTC
SC - Ura
CD Strain
YKO/CD Strains
YKO-Btx Strains
YKO/CD Strains
SC – Ura + G418
YKO/CD Strains
YKO Strains
YKO-Btx Strains
CD Strain
YKO-Btx Strains
YKO/CD Strains
YKO Strains
CD Strain
# 1,000 cells plated
10,000
Cells
plated

## Slide 6
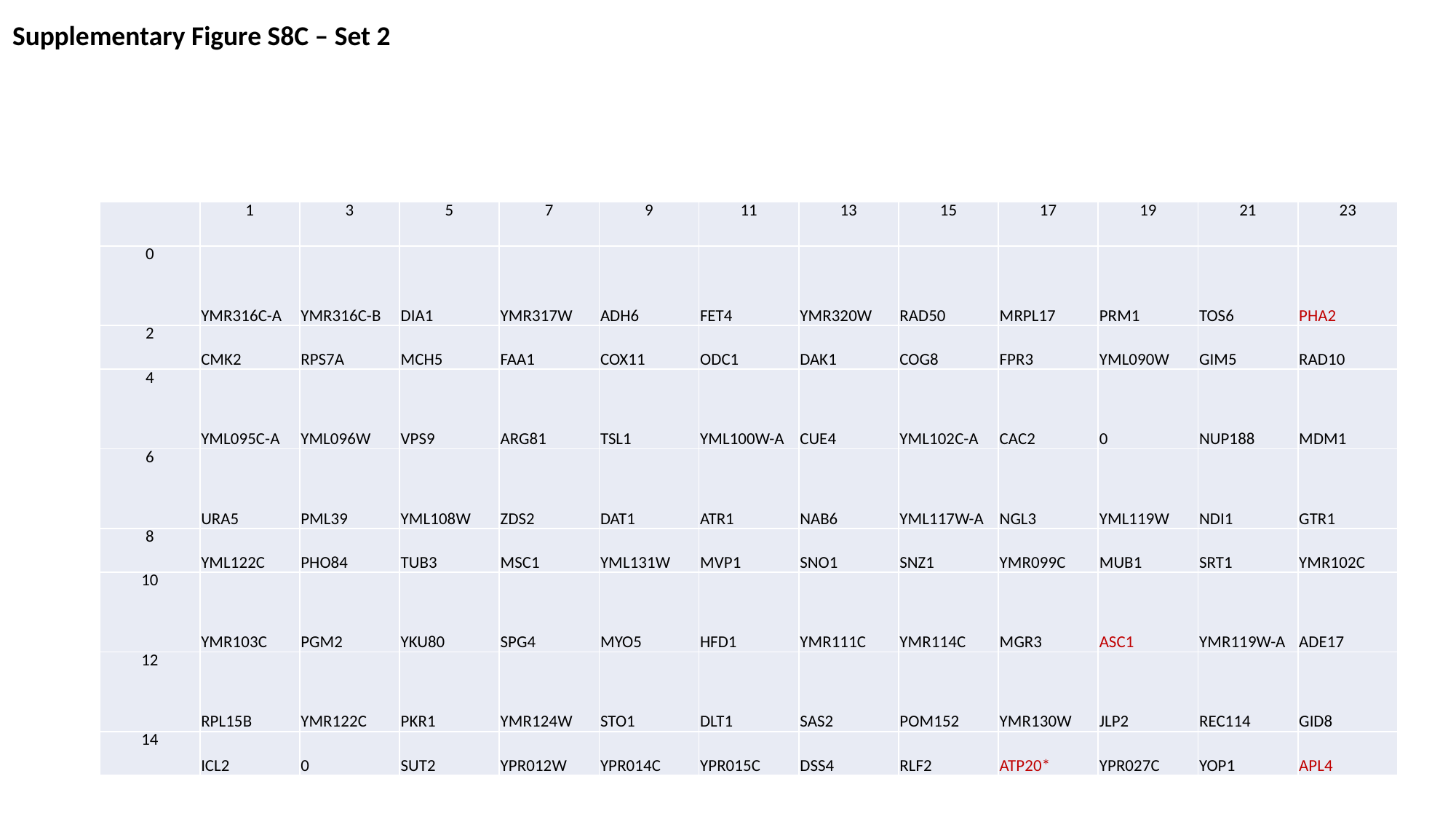

Supplementary Figure S8C – Set 2
| | 1 | 3 | 5 | 7 | 9 | 11 | 13 | 15 | 17 | 19 | 21 | 23 |
| --- | --- | --- | --- | --- | --- | --- | --- | --- | --- | --- | --- | --- |
| 0 | YMR316C-A | YMR316C-B | DIA1 | YMR317W | ADH6 | FET4 | YMR320W | RAD50 | MRPL17 | PRM1 | TOS6 | PHA2 |
| 2 | CMK2 | RPS7A | MCH5 | FAA1 | COX11 | ODC1 | DAK1 | COG8 | FPR3 | YML090W | GIM5 | RAD10 |
| 4 | YML095C-A | YML096W | VPS9 | ARG81 | TSL1 | YML100W-A | CUE4 | YML102C-A | CAC2 | 0 | NUP188 | MDM1 |
| 6 | URA5 | PML39 | YML108W | ZDS2 | DAT1 | ATR1 | NAB6 | YML117W-A | NGL3 | YML119W | NDI1 | GTR1 |
| 8 | YML122C | PHO84 | TUB3 | MSC1 | YML131W | MVP1 | SNO1 | SNZ1 | YMR099C | MUB1 | SRT1 | YMR102C |
| 10 | YMR103C | PGM2 | YKU80 | SPG4 | MYO5 | HFD1 | YMR111C | YMR114C | MGR3 | ASC1 | YMR119W-A | ADE17 |
| 12 | RPL15B | YMR122C | PKR1 | YMR124W | STO1 | DLT1 | SAS2 | POM152 | YMR130W | JLP2 | REC114 | GID8 |
| 14 | ICL2 | 0 | SUT2 | YPR012W | YPR014C | YPR015C | DSS4 | RLF2 | ATP20\* | YPR027C | YOP1 | APL4 |

## Slide 7
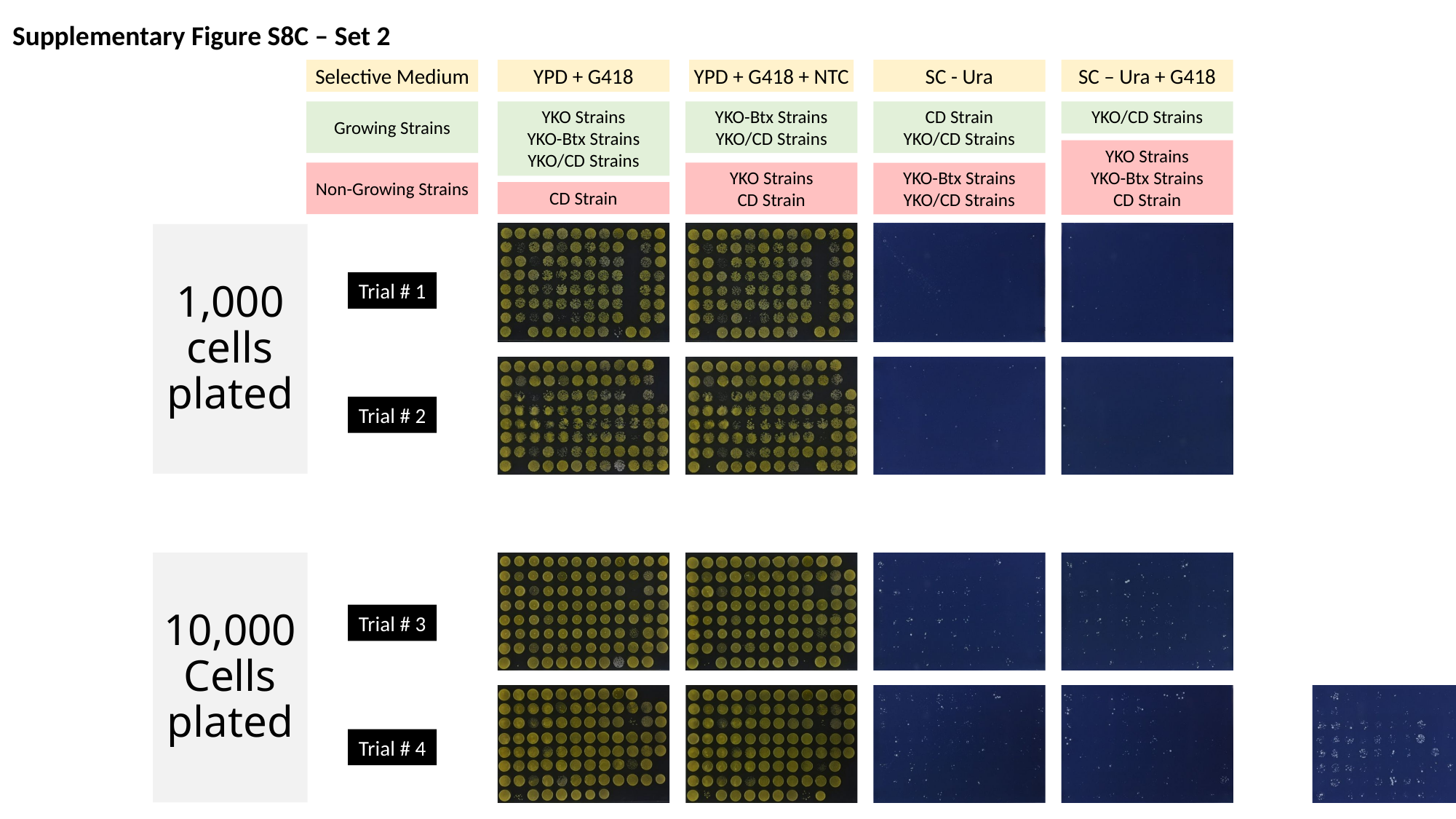

Supplementary Figure S8C – Set 2
Selective Medium
YPD + G418
YPD + G418 + NTC
YKO-Btx Strains
YKO/CD Strains
SC - Ura
SC – Ura + G418
YKO Strains
YKO-Btx Strains
YKO/CD Strains
CD Strain
CD Strain
YKO/CD Strains
YKO-Btx Strains
YKO/CD Strains
YKO/CD Strains
Growing Strains
YKO Strains
YKO-Btx Strains
CD Strain
YKO Strains
CD Strain
Non-Growing Strains
# 1,000 cells plated
Trial # 1
Trial # 2
10,000
Cells
plated
Trial # 3
Trial # 4

## Slide 8
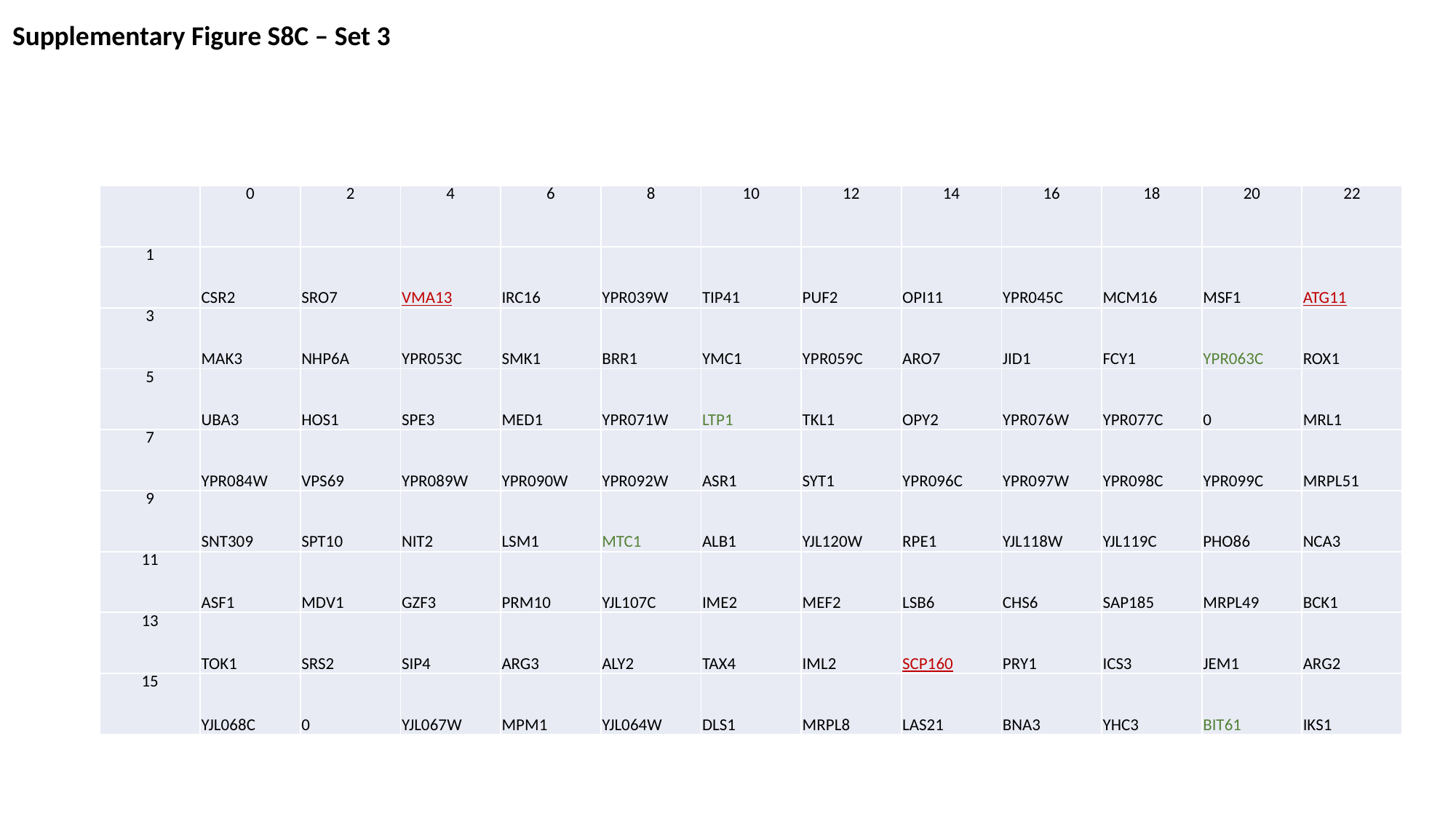

Supplementary Figure S8C – Set 3
| | 0 | 2 | 4 | 6 | 8 | 10 | 12 | 14 | 16 | 18 | 20 | 22 |
| --- | --- | --- | --- | --- | --- | --- | --- | --- | --- | --- | --- | --- |
| 1 | CSR2 | SRO7 | VMA13 | IRC16 | YPR039W | TIP41 | PUF2 | OPI11 | YPR045C | MCM16 | MSF1 | ATG11 |
| 3 | MAK3 | NHP6A | YPR053C | SMK1 | BRR1 | YMC1 | YPR059C | ARO7 | JID1 | FCY1 | YPR063C | ROX1 |
| 5 | UBA3 | HOS1 | SPE3 | MED1 | YPR071W | LTP1 | TKL1 | OPY2 | YPR076W | YPR077C | 0 | MRL1 |
| 7 | YPR084W | VPS69 | YPR089W | YPR090W | YPR092W | ASR1 | SYT1 | YPR096C | YPR097W | YPR098C | YPR099C | MRPL51 |
| 9 | SNT309 | SPT10 | NIT2 | LSM1 | MTC1 | ALB1 | YJL120W | RPE1 | YJL118W | YJL119C | PHO86 | NCA3 |
| 11 | ASF1 | MDV1 | GZF3 | PRM10 | YJL107C | IME2 | MEF2 | LSB6 | CHS6 | SAP185 | MRPL49 | BCK1 |
| 13 | TOK1 | SRS2 | SIP4 | ARG3 | ALY2 | TAX4 | IML2 | SCP160 | PRY1 | ICS3 | JEM1 | ARG2 |
| 15 | YJL068C | 0 | YJL067W | MPM1 | YJL064W | DLS1 | MRPL8 | LAS21 | BNA3 | YHC3 | BIT61 | IKS1 |

## Slide 9
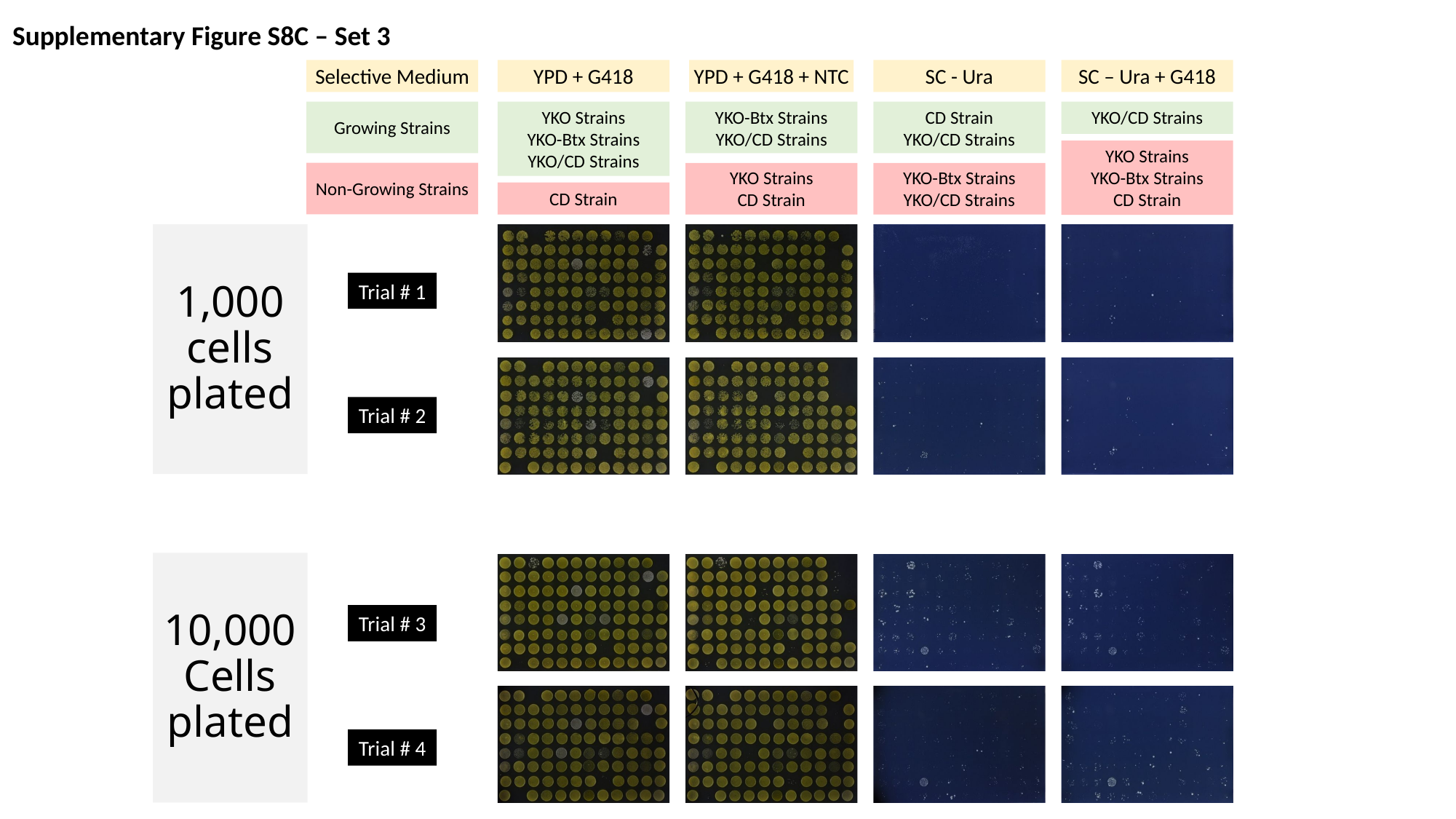

Supplementary Figure S8C – Set 3
Selective Medium
Growing Strains
Non-Growing Strains
Trial # 1
Trial # 2
Trial # 3
Trial # 4
YPD + G418
YKO Strains
YKO-Btx Strains
YKO/CD Strains
CD Strain
YPD + G418 + NTC
YKO-Btx Strains
YKO/CD Strains
YKO Strains
CD Strain
SC - Ura
CD Strain
YKO/CD Strains
YKO-Btx Strains
YKO/CD Strains
SC – Ura + G418
YKO/CD Strains
YKO Strains
YKO-Btx Strains
CD Strain
# 1,000 cells plated
10,000
Cells
plated

## Slide 10
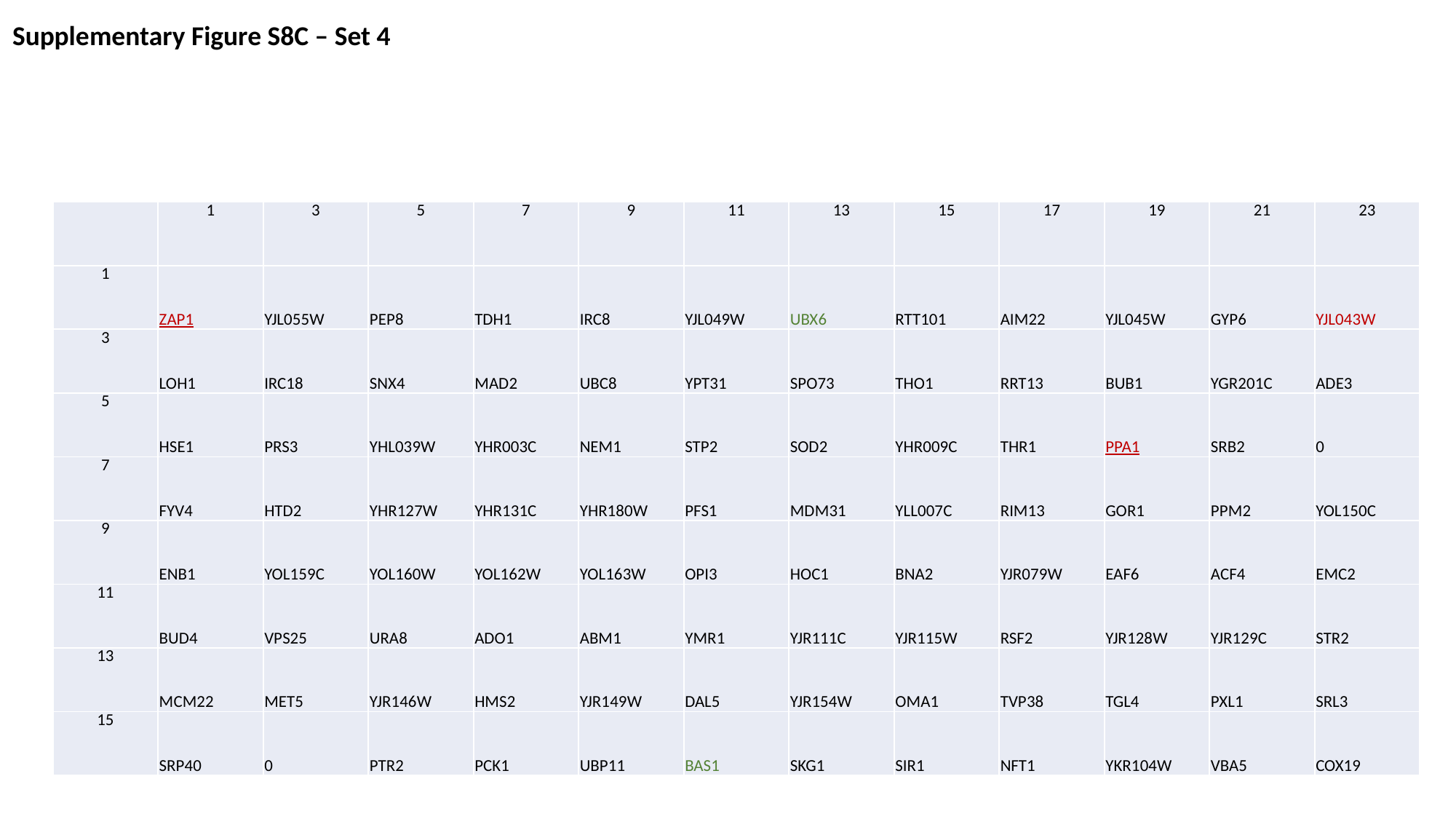

Supplementary Figure S8C – Set 4
| | 1 | 3 | 5 | 7 | 9 | 11 | 13 | 15 | 17 | 19 | 21 | 23 |
| --- | --- | --- | --- | --- | --- | --- | --- | --- | --- | --- | --- | --- |
| 1 | ZAP1 | YJL055W | PEP8 | TDH1 | IRC8 | YJL049W | UBX6 | RTT101 | AIM22 | YJL045W | GYP6 | YJL043W |
| 3 | LOH1 | IRC18 | SNX4 | MAD2 | UBC8 | YPT31 | SPO73 | THO1 | RRT13 | BUB1 | YGR201C | ADE3 |
| 5 | HSE1 | PRS3 | YHL039W | YHR003C | NEM1 | STP2 | SOD2 | YHR009C | THR1 | PPA1 | SRB2 | 0 |
| 7 | FYV4 | HTD2 | YHR127W | YHR131C | YHR180W | PFS1 | MDM31 | YLL007C | RIM13 | GOR1 | PPM2 | YOL150C |
| 9 | ENB1 | YOL159C | YOL160W | YOL162W | YOL163W | OPI3 | HOC1 | BNA2 | YJR079W | EAF6 | ACF4 | EMC2 |
| 11 | BUD4 | VPS25 | URA8 | ADO1 | ABM1 | YMR1 | YJR111C | YJR115W | RSF2 | YJR128W | YJR129C | STR2 |
| 13 | MCM22 | MET5 | YJR146W | HMS2 | YJR149W | DAL5 | YJR154W | OMA1 | TVP38 | TGL4 | PXL1 | SRL3 |
| 15 | SRP40 | 0 | PTR2 | PCK1 | UBP11 | BAS1 | SKG1 | SIR1 | NFT1 | YKR104W | VBA5 | COX19 |

## Slide 11
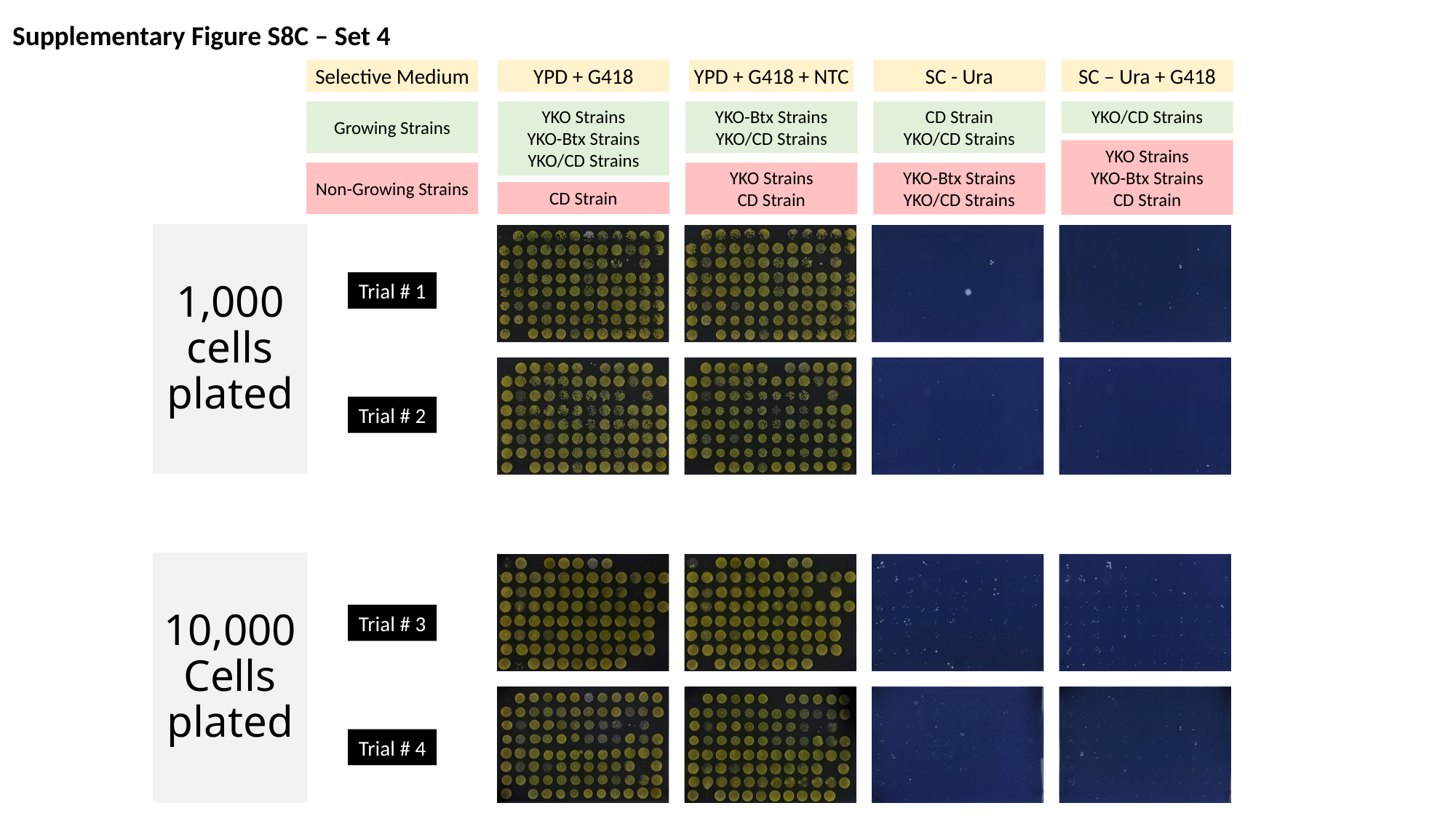

Supplementary Figure S8C – Set 4
Selective Medium
Growing Strains
Non-Growing Strains
Trial # 1
Trial # 2
Trial # 3
Trial # 4
YPD + G418
YKO Strains
YKO-Btx Strains
YKO/CD Strains
CD Strain
YPD + G418 + NTC
YKO-Btx Strains
YKO/CD Strains
YKO Strains
CD Strain
SC - Ura
CD Strain
YKO/CD Strains
YKO-Btx Strains
YKO/CD Strains
SC – Ura + G418
YKO/CD Strains
YKO Strains
YKO-Btx Strains
CD Strain
# 1,000 cells plated
10,000
Cells
plated
