# Supplementary material for: CRI-SPA: a high-throughput method for systematic genetic editing of yeast libraries
Source: Nucleic Acids Res. 2023 Aug 12;51(17):e91. doi: 10.1093/nar/gkad656 (PMC10516668; doi:10.1093/nar/gkad656)
Supplement: gkad656_supplemental_files [file gkad656_supplemental_files.zip › Supplementary Material.docx]

**CRI-SPA – a high-throughput method for systematic genetic editing of yeast libraries**

Paul Cachera*, Helén Olsson*, Hilde Coumou*, Mads L. Jensen, Benjamín J. Sánchez, Tomas Strucko, Marcel van den Broek, Jean-Marc Daran, Michael K. Jensen, Nikolaus Sonnenschein, Michael Lisby, Uffe H. Mortensen.


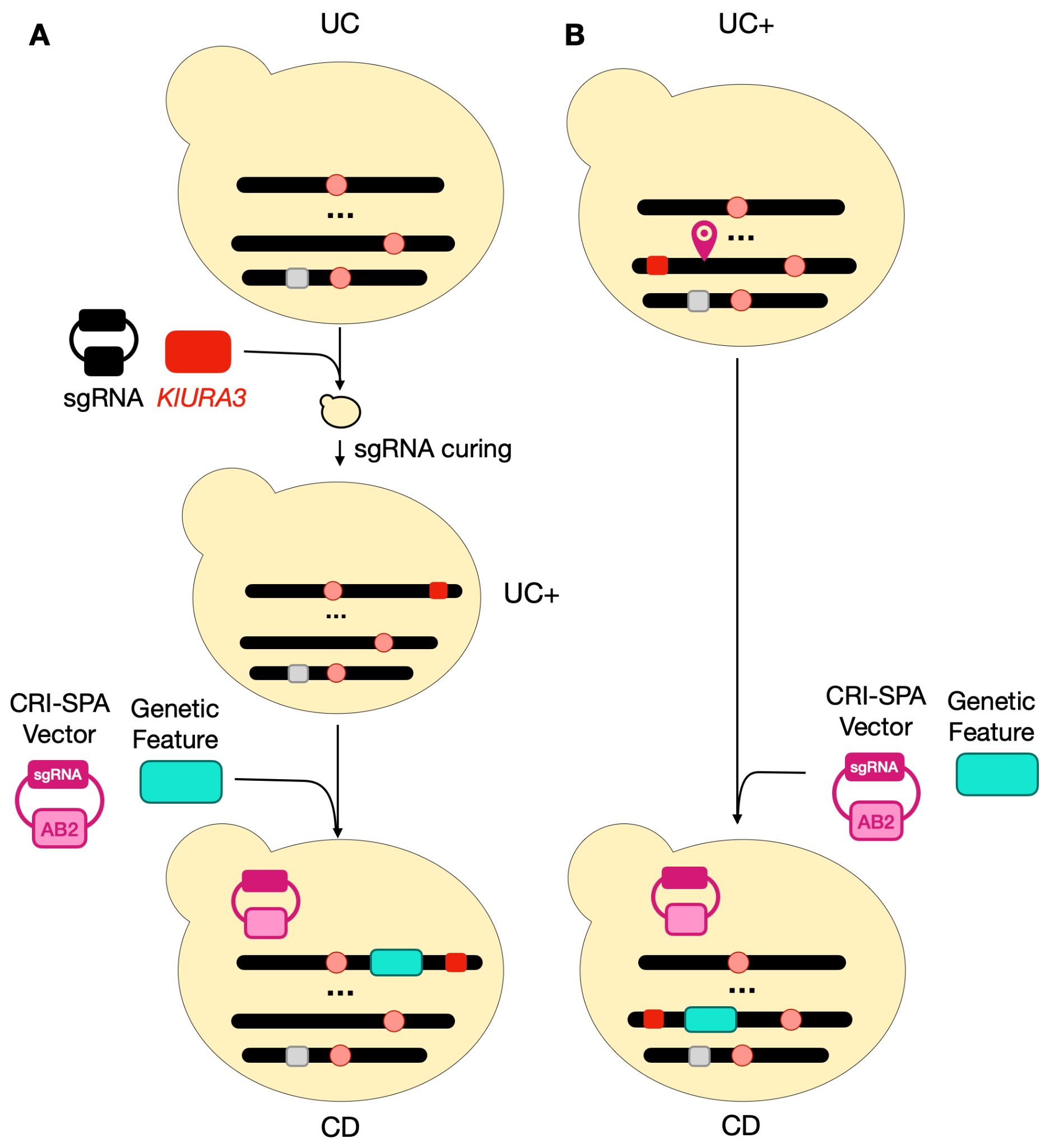


**Supplementary Figure S1. CRI-SPA Donor (CD) Strain construction.** Construction of a CD strain from a Universal CRI-SPA (UC) depends on the nature of the genetic feature of interest. **(A)** This method employs the basic UC strains and can be used to make any CD strain, but is typically used if the transfer site has not previously been used in a CRI-SPA transfer experiment. In this case, the CD strain needs to be constructed in three steps using a UC strain. Firstly, a *Kl_URA3* marker needs to be incorporated on the telomeric side of the transfer site with the help of an sgRNA plasmid. Secondly, the strain is cured for the sgRNA plasmid to form an UC+ strain. Thirdly, an appropriate gene-targeting substrate containing the genetic feature is transformed into the UC+ strain together with the relevant CRI-SPA vector to produce the CD strain. **(B)** For construction of a set of CD strains harboring different gene-expression cassettes in the same common insertion site, a common UC+ strain serves as the starting point for construction of all strains in the set to keep strain construction work at a minimum. In this case, construction of the first CD in the set follows track (A) and is constructed in two steps. The UC+ strain created in this process can subsequently be used as a starting point for the construction of the remaining CD strains of the set, which can be made in a single step as shown in panel B. In this manuscript, we describe a UC+ strain, UC+:XII-5, which comes with a pre-integrated *Kl_URA3* marker between the defined expression site XII-5 and the corresponding telomere. Moreover, an efficient CRI-SPA vector for this site is also available (pHO-XII-5 see Supplementary Table S2). For convenience, UC- and UC+:XII-5 strains are available in both mating types (see Supplementary Table S1).


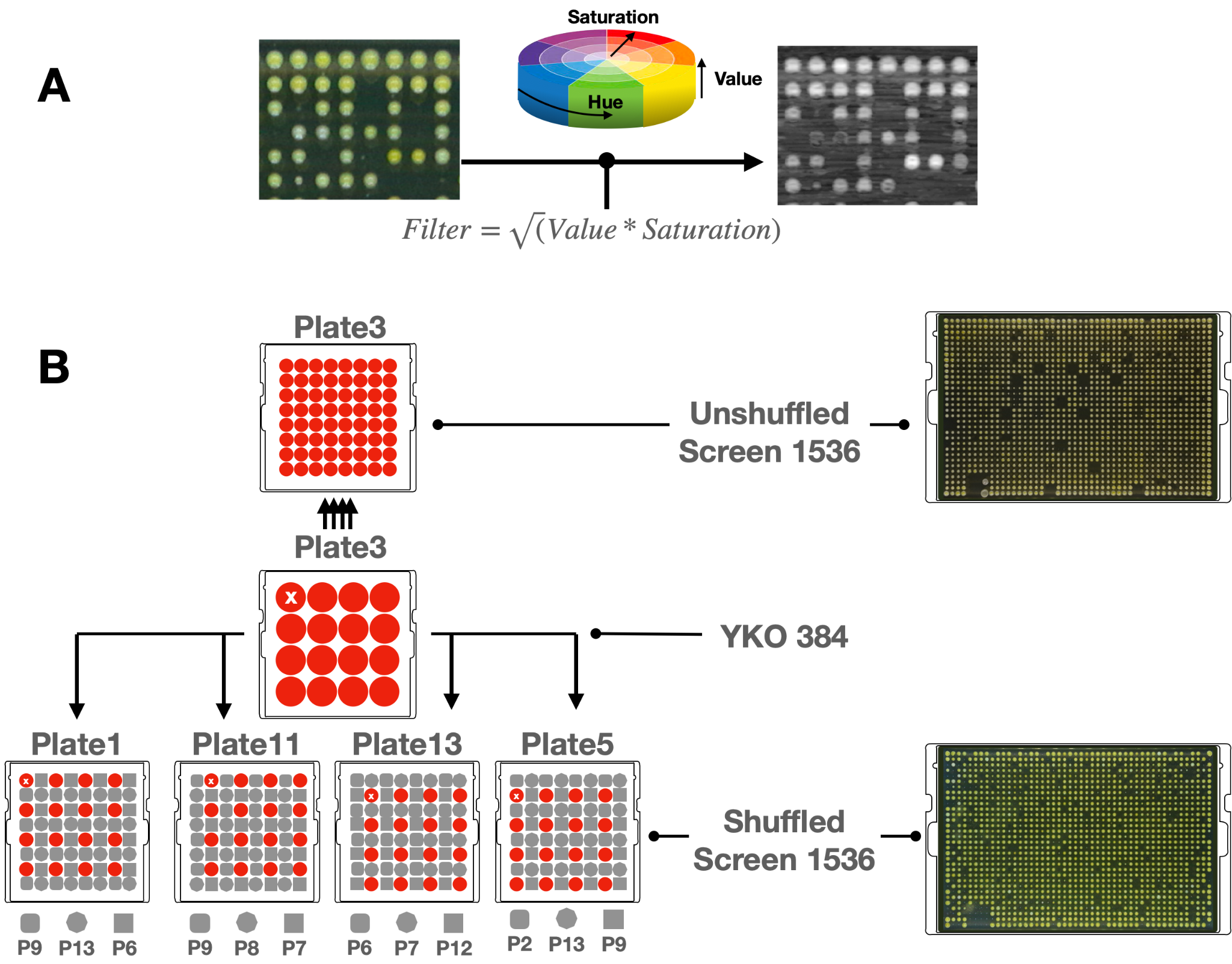


**Supplementary Figure S2. Colony Shuffling Scheme and Color FIltering. (A)** Left: first version of the screen. During expansion from 386 to 1544 format, individual mutant colonies are pinned as colony quadruplicates in a square of the same plate. As a result, each quadruplicate colony is affected by identical agar quality and similar neighbor effects. Blue square shows YOR1 mutant. Right, second version of the screen. During expansion from 386 to 1544 format, individual mutant colonies are shuffled across random screen-plates. For example, YKO plate 1 is pinned on screen plates 7, 3, 6 and 10. This increases the diversity of neighbors and reduces potential agar gradient biases. **(B)** Heuristic to quantify yellow intensity. Yellowness was quantified as the geometric mean between Value and Saturation in the HSV domain.

**
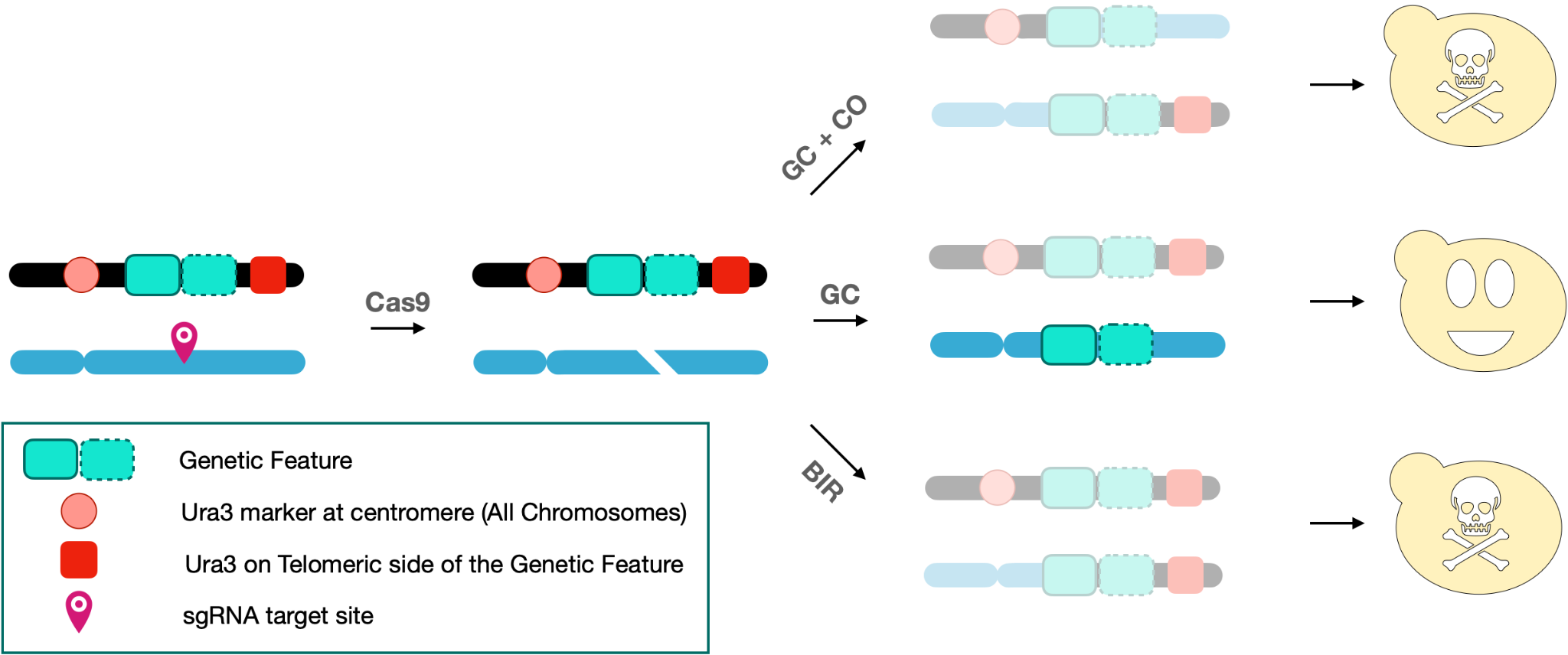
**

**Supplementary Figure S3. Cas9 induced crossover and break induced replication produce chimeric strains.** In CRI-SPA, transfer of the desirable genetic information from the donor to the target site by homology directed repair of a DNA DSB in the target site is performed by gene conversion (GC). However, homology directed repair of the DNA DSB may also occur via GC accompanied by a crossing over event (GC + CO) or by break induced replication (BIR). In both cases, the final strain after CRI-SPA will contain an undesirable chimeric chromosome consisting of donor and target chromosome sections. In our CRI-SPA system, strains created by GC + CO or BIR are counterselected in step 4 of the CRI-SPA procedure (5-FOA counter selection) as we have incorporated a *K. lactis* *URA3* marker between the donor site and the telomere; and this *URA3* marker will be transferred to the target chromosome in case that repair of the CRISPR induced DNA DSB involves GC + CO or BIR.

**
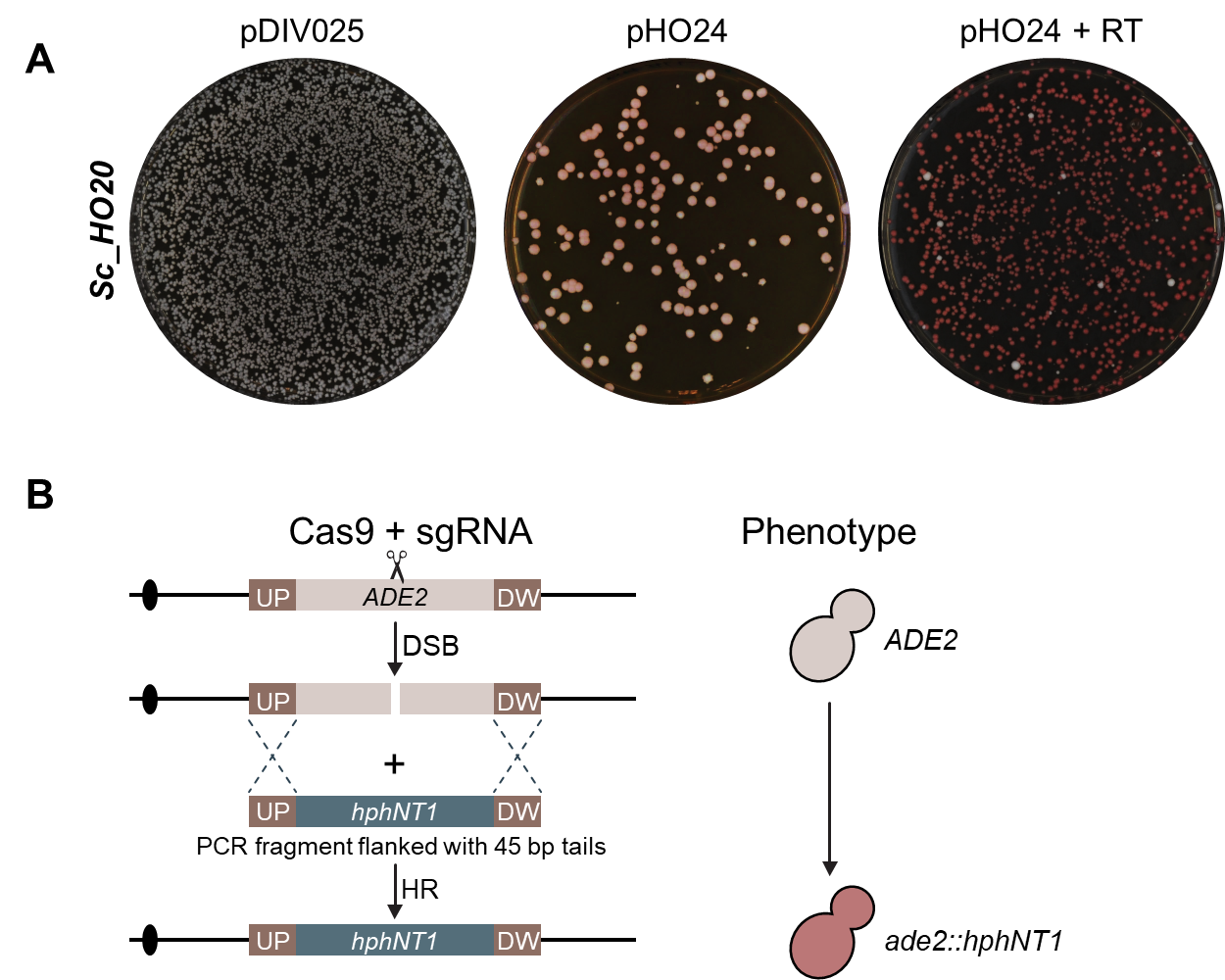
**

**Supplementary Figure S4. TAPE experiment to assess Cas9/gRNA cleavage efficiency of a target protospacer.** A TAPE (tool assess protospacer efficiency) experiment (Vanegas et al., 2017) was performed to determine how efficiently the CRISPR nuclease Cas9/*ADE2-*gRNA cuts the *ADE2* locus in a UC strain (UC+XII5α). **(A)** Plates showing the numbers of transformants obtained after transformation of the UC strain with an empty plasmid pDIV025, with pHO24 encoding the *ADE2*-sgRNA, and after co-transformation with pHO24 and a repair template (RT) composed by a *ade2Δ::hphNT1* PCR fragment flanked with 45 bp long UP and DW targeting regions. The amounts of vector DNA used in the three transformation experiments were stoichiometrically the same. (**B**) Cartoon showing HR mediated repair of the Cas9/*ADE2-*gRNA induced DNA DSB in ADE2 using *ade2Δ::hphNT1* as repair template for breaks induced at *ADE2*. The development of a red phenotype resulting from the *ade2Δ::hphNT1* mutation is indicated. The high number of transformants obtained with pDIV025 relative to the number obtained with pHO24 indicates that lethal DNA DSBs are produced by the Cas9/*ADE2-s*gRNA CRISPR nuclease in the UC strain transformed with pHO24. This conclusion was supported by two observations. Firsty, the number of transformants obtained after transformation with pHO24 increases to match the number of transformants obtained with pDIV25 when the repair fragment was included in the reaction allowing for efficient DNA DSB repair by homologous recombination. Secondly, virtually all transformations obtaied in the co-transformation experiment were red as *ADE2* was delete

**
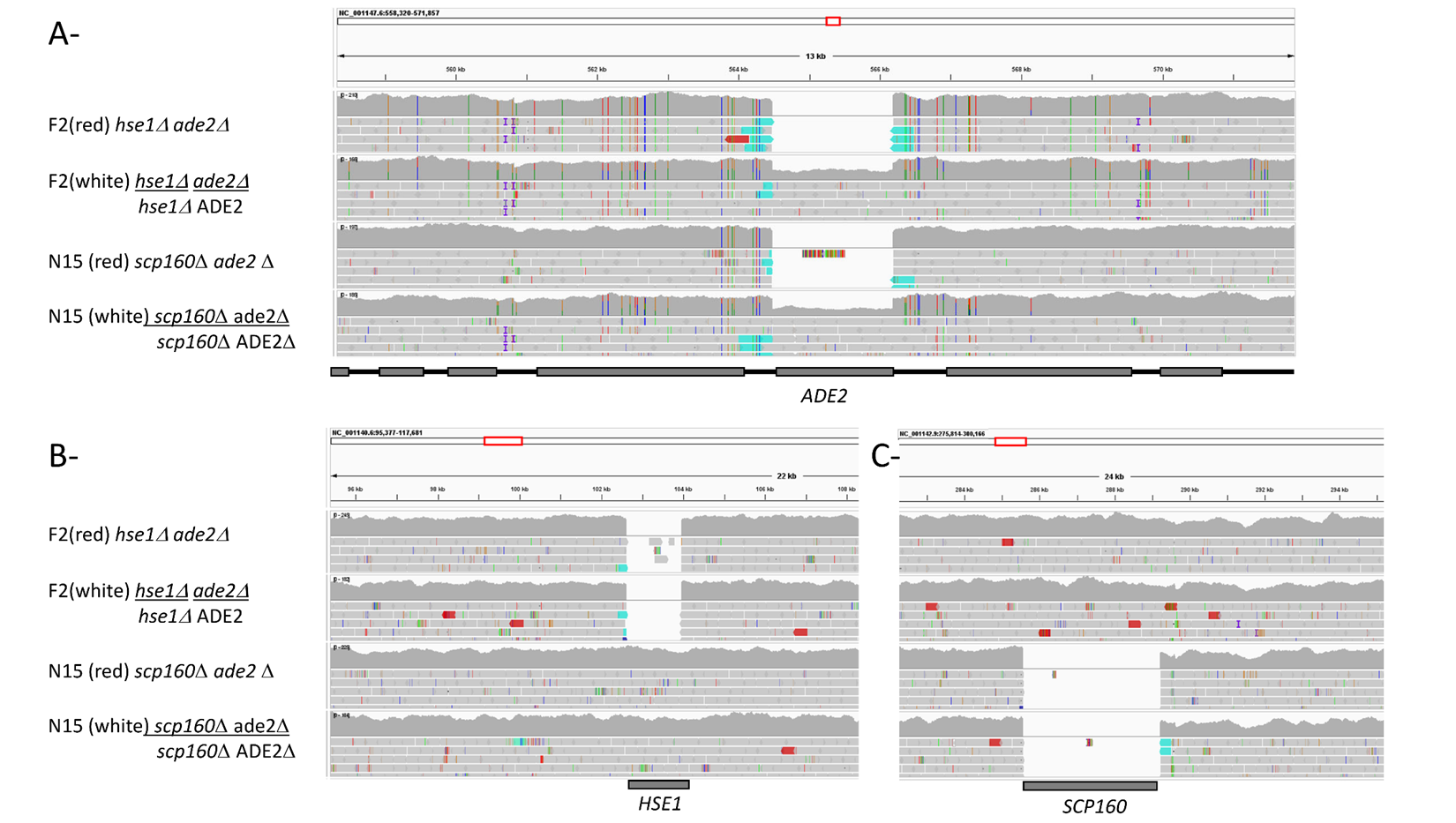
**

**Supplementary Figure S5. Sequencing of selected CRI-SPA strain after CRI-SPA mediated *ade2*Δ*::hphNT1* cassette transfer from CD-*ade2*Δ strain to plate 9 strains of the YKO library.** IGV screenshot of mapping of sequencing reads of red and white isolates of library clones F2 and N15, which harbor an *HSE1* and *SCP160* deletion, respectively, after CRI-SPA transformation for *ADE2* deletion on genome of the Saccharomyces cerevisiae strain CEN.PK113-7D. **(A)** shows illumina sequencing read mapping at *ADE2* locus. **(B)** shows mapping at *HSE1* locus and **(C)** at locus *SCP160* locus.


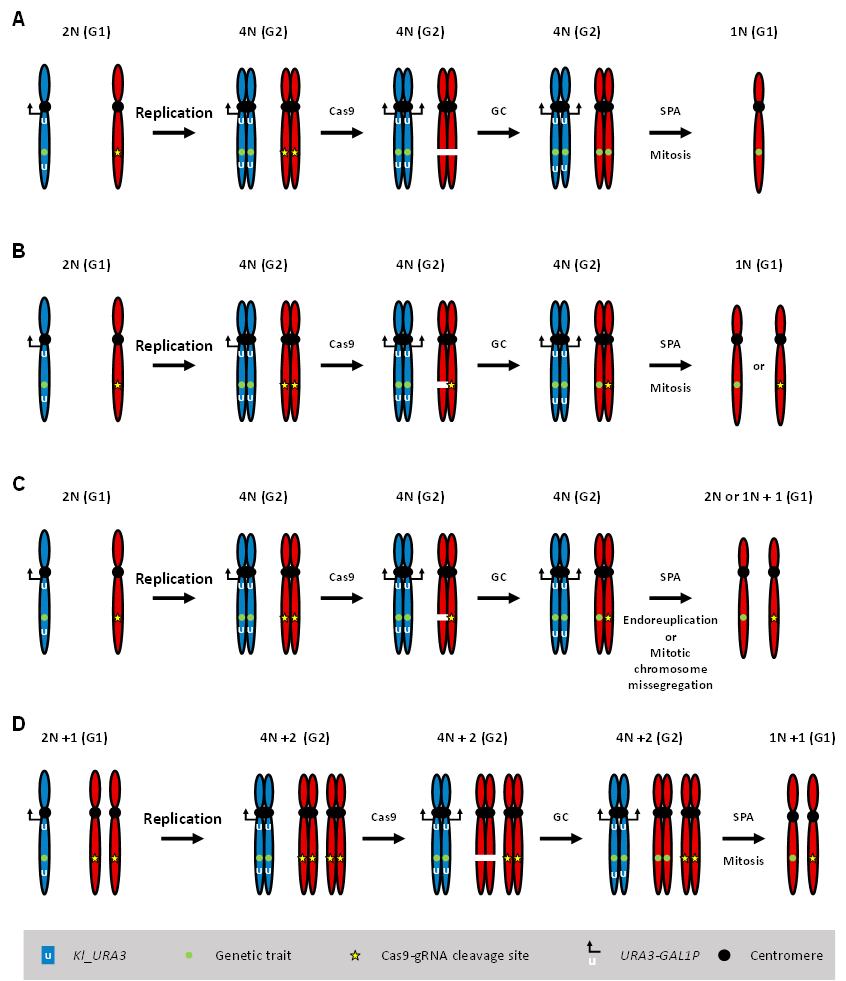


**Supplementary Figure S6. Models explaining background cells generated by CRI-SPA.** Background may be produced in cells containing an extra copy of the target recipient chromosome. The extra chromosome may be due to the recipient strain being aneuploid from the beginning, or due to endoreduplication during SPA. If Cas9 only cleaves the target sequence in one of the two target chromosomes during CRI-SPA, CRI-SPA mediated transfer of a genetic trait from a donor chromosome (blue) to the corresponding recipient chromosome (red) is incomplete. Different scenarious are illustrated in the panel below with the diploid strain formed by mating of the CD to a recipient strain during CRI-SPA as a starting point. **(A)** in normal CRI-SPA, Cas9 will cleave both recipient target chromatids in the diploid cells formed by fusion of donor and recipient cells. Repair of the breaks by gene conversion (GC) transfers the genetic trait from a donor chromatid to the corresponding recipient chromatids. Haploidization by SPA generates recipient chromosomes Containing the genetic trait of interest. **(B)** If CRISPR mediated GC takes place after the target locus has been replicated, the possibility exists that only one of the two recipient chromatids may be cleaved by Cas9 in the timeframe of the CRI-SPA process where Cas9 is active. If so, only one of the two recipient chromatids will receive genetic information from the corresponding donor chromatid. In this case cells may contain either a recipient chromosome containing the desired genetic trait, or one that does not. If the genetic trait is selected for, the latter type of cells will die. If the genetic trait is not selected for, the latter type of cells will survive and produce background. **(C)** Same scenario as in B) except that the process does not continue with a perfect mitosis, rather the entire genome or the target chromosome is duplicated as the result of endoreduplication or the number of target chromosomes is doubled due to chromosome missegregation. As a result, cells containing heterozygous recipient disomes are formed where only one chromosome contains the desired genetic trait. Such cells are expected to survive in the presence or absence of selection for the genetic trait to produce background. **(D)**. The starting recipient cell is aneuploid and contains two target chromosomes of which only one contains the Btx-cassette. Like in the other scenarios, incomplete cleavage by Cas9 produce one modified and one un-modified target chromosome in the final cell.

**
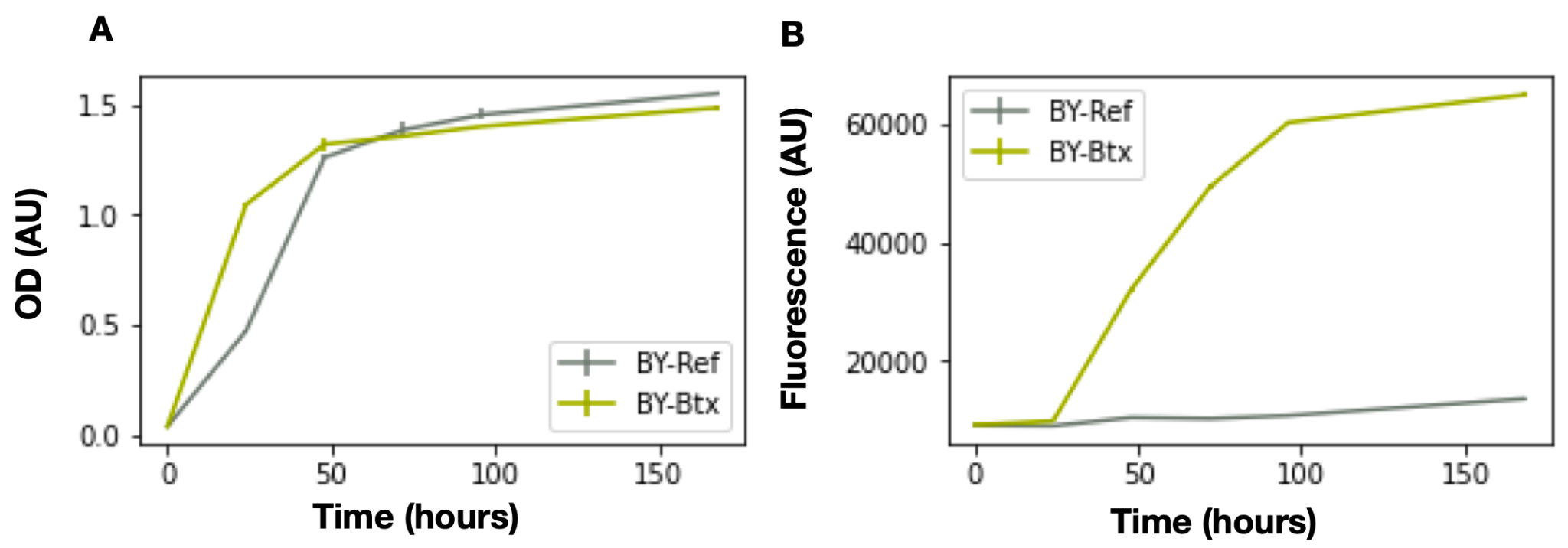
**

**Supplementary Figure S7. Growth profile and Betaxanthin production of BY-Btx.**

**(A)** Growth profile of the betaxanthin producing BY-btx strain and the reference background strain BY-Ref. **(B)** Betaxanthin synthesis for the same samples measured by fluorescence. Error bars are experimental triplicates of the same biological sample.

**SEE SUPPLEMENTARY FIGURE S8 DOCUMENT**

**Supplementary Figure S8. Analysis of CRI-SPA colonies for the presence of unmodified YKO-, CD-, and diploid cells in the absence of selection for the Btx-Cassette during CRI-SPA mediated transfer**


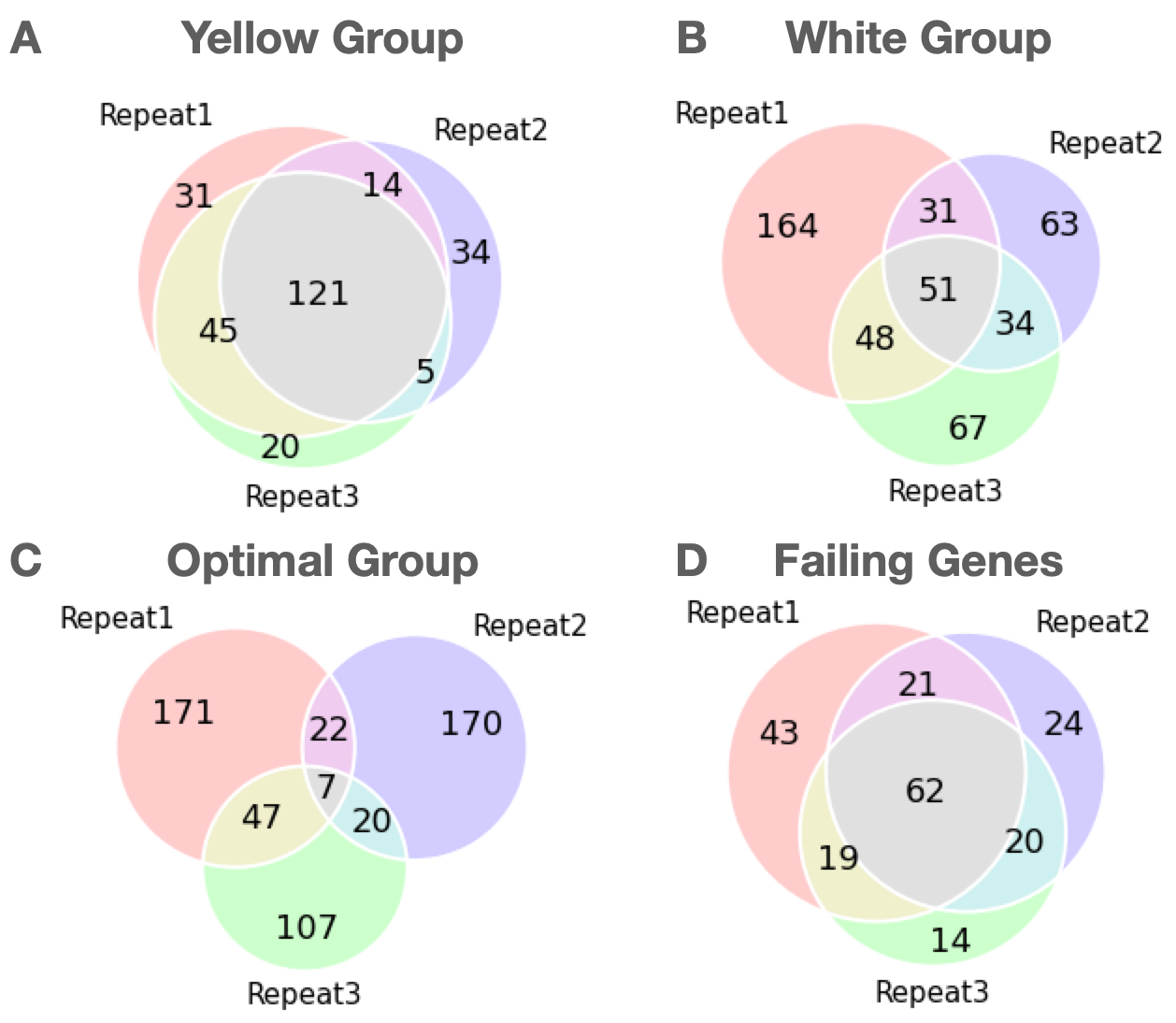
**Supplementary Figure S9. Screen repeats overlap.** Overlap in gene groups between three screen repeats. **(A)** Yellow Group, top betaxanthin (Yellow intensity > 1.2 std above screen mean). **(B)** White Group, lowest betaxanthin (Yellow intensity < 1.2 std below screen mean). **(C)** Cyan group (i.e.“cyan group”, 0.8 < Yellow intensity < 1.2 std above screen mean). **(D)** Failing genes, genes producing 0 colonies after CRI-SPA procedure.

**SUPPLEMENTARY TABLES**

**SUPPLEMENTARY TABLE S1: Strains Used in this Study**

| **Strain Name** | **Parent** | **Genotype** | **Source** |
| --- | --- | --- | --- |
| W8164-2B | - | MATα CEN1-16::pGal1-KlURA3  ADE2 can1-100 his3-11,15 leu2-3,112 LYS2 met17 trp1-1 ura3-1 RAD5 | Reid et al., 2011 |
| **W8164-2C** | - | MATa CEN1-16::pGAL1-KlURA3 ADE2 can1-100 his3-11,15 leu2-3,112 LYS2 met17 trp1-1 ura3-1 RAD5 | Reid et al., 2011 |
| **BY4741** | - | MATa his3Δ1 leu2Δ0 met15Δ0 ura3Δ0 | Baker Brachmann et al., 1998 |
| **UC1α** | W8164-2B | MATα CEN1-16::pGAL1-KlURA3 ADE2 can1-100 his3-11,15 leu2-3,112 LYS2 met17 trp1-1 ura3-1 RAD5  X-3::pTEF1-SpCas9(Hs)-tCYC1-loxP-KlLEU2 | This study |
| **UC1a** | W8164-2C | MATa CEN1-16::pGAL1-KlURA3 ADE2 can1-100 his3-11,15 leu2-3,112 LYS2 met17 trp1-1 ura3-1 RAD5  X-3::pTEF1-SpCas9(Hs)-tCYC1-loxP-KlLEU2 | This study |
| **UC+XII5α** | W8164-2B | MATα CEN1-16::pGAL1-KlURA3 ADE2 can1-100 his3-11,15 leu2-3,112 LYS2 met17 trp1-1 ura3-1 RAD5  X-3::pTEF1-Spcas9(Hs)-tCYC1-loxP-KlLEU2 (dw XII-5 IS) KlURA3 | This study |
| **UC+XII5a** | W8164-2B | MATa CEN1-16::pGAL1-KlURA3 ADE2 can1-100 his3-11,15 leu2-3,112 LYS2 met17 trp1-1 ura3-1 RAD5  X-3::pTEF1-SpCas9(Hs)-tCYC1-loxP-KlLEU2 (dw XII-5 IS) KlURA3 | This study |
| **CD-*ade2Δ*** | UCS1α | MATα CEN1-16::pGAL1-KlURA3can1-100 his3-11,15 leu2-3,112 LYS2 met17 trp1-1 ura3-1 RAD5 X-3::pTEF1-SpCas9-tCYC1-loxP-KlLEU2-loxP KlURA3(dw XII-5 IS) | This study |
| **CD-1** | **UCS1α** | MATα CEN1-16::pGAL1-KlURA3can1-100 his3-11,15 leu2-3,112 LYS2 met17 trp1-1 ura3-1 RAD5 X-3::pTEF1-SpCas9-tCYC1-loxP-KlLEU2-loxP KlURA3(dw XII-5 IS) | This study |
| **CD-2** | UCS1a | MATa CEN1-16::pGAL1-KlURA3can1-100 his3-11,15 leu2-3,112 LYS2 met17 trp1-1 ura3-1 RAD5  X-3::pTEF1-SpCas9-tCYC1-loxP-KlLEU2-loxP  KlURA3(dw XII-5 IS) | This study |
| **CD-btx** | **UCS2α** | *MAT*α CEN1-16::pGal1-*KlURA3 can1-100 his3-11,15 leu2-3,112 LYS2 met17 trp1-1 ura3-1 RAD5* X-3::p*TEF1*-SpCas9-t*CYC1*-loxP-*KlLEU2* (dw XII-5 IS) *KlURA3*  XII-5::*TADH1-ARO7G141S-PTEF1-PPGK1-ARO4K229L-TTDH3-*  BvCYP76AD1-PTPI1-PCCW12-MjDOD-Pgossypii:NatMX:tTEF1gossypii | This study |
| BY-Btx | BY4741 | MATa his3Δ1 leu2Δ0 met15Δ0 ura3Δ0 XII-5::*TADH1-ARO7G141S-PTEF1-PPGK1-ARO4K229L-TTDH3-*  BvCYP76AD1-PTPI1-PCCW12-MjDOD-Pgossypii:NatMX:tTEF1gossypii | This study |
| **ScBTX007** | BY-Btx | MATa his3Δ1 leu2Δ0 met15Δ0 ura3Δ0 **dld2Δ0** XII-5::*TADH1-ARO7G141S-PTEF1-PPGK1-ARO4K229L-TTDH3-*  BvCYP76AD1-PTPI1-PCCW12-MjDOD-Pgossypii:NatMX:tTEF1gossypii | This study |
| **ScBTX008** | BY-Btx | MATa his3Δ1 leu2Δ0 met15Δ0 ura3Δ0 kgd**2Δ0** XII-5::*TADH1-ARO7G141S-PTEF1-PPGK1-ARO4K229L-TTDH3-*  BvCYP76AD1-PTPI1-PCCW12-MjDOD-Pgossypii:NatMX:tTEF1gossypii | This study |
| **ScBTX009** | BY-Btx | MATa his3Δ1 leu2Δ0 met15Δ0 ura3Δ0 mip1**Δ0** XII-5::*TADH1-ARO7G141S-PTEF1-PPGK1-ARO4K229L-TTDH3-*  BvCYP76AD1-PTPI1-PCCW12-MjDOD-Pgossypii:NatMX:tTEF1gossypii | This study |
| **ScBTX011** | BY-Btx | MATa his3Δ1 leu2Δ0 met15Δ0 ura3Δ0 rcr2**Δ0** XII-5::*TADH1-ARO7G141S-PTEF1-PPGK1-ARO4K229L-TTDH3-*  BvCYP76AD1-PTPI1-PCCW12-MjDOD-Pgossypii:NatMX:tTEF1gossypii | This study |
| **ScBTX012** | BY-Btx | MATa his3Δ1 leu2Δ0 met15Δ0 ura3Δ0 sgm1**Δ0** XII-5::*TADH1-ARO7G141S-PTEF1-PPGK1-ARO4K229L-TTDH3-*  BvCYP76AD1-PTPI1-PCCW12-MjDOD-Pgossypii:NatMX:tTEF1gossypii | This study |
| **ScBTX013** | BY-Btx | MATa his3Δ1 leu2Δ0 met15Δ0 ura3Δ0 shy1**Δ0** XII-5::*TADH1-ARO7G141S-PTEF1-PPGK1-ARO4K229L-TTDH3-*  BvCYP76AD1-PTPI1-PCCW12-MjDOD-Pgossypii:NatMX:tTEF1gossypii | This study |
| **ScBTX014** | BY-Btx | MATa his3Δ1 leu2Δ0 met15Δ0 ura3Δ0 vps34**Δ0** XII-5::*TADH1-ARO7G141S-PTEF1-PPGK1-ARO4K229L-TTDH3-*  BvCYP76AD1-PTPI1-PCCW12-MjDOD-Pgossypii:NatMX:tTEF1gossypii | This study |
| **ScBTX016** | BY-Btx | MATa his3Δ1 leu2Δ0 met15Δ0 ura3Δ0 yer084W**Δ0** XII-5::*TADH1-ARO7G141S-PTEF1-PPGK1-ARO4K229L-TTDH3-*  BvCYP76AD1-PTPI1-PCCW12-MjDOD-Pgossypii:NatMX:tTEF1gossypii | This study |
| **ScBTX018** | BY-Btx | MATa his3Δ1 leu2Δ0 met15Δ0 ura3Δ0 cox12**Δ0** XII-5::*TADH1-ARO7G141S-PTEF1-PPGK1-ARO4K229L-TTDH3-*  BvCYP76AD1-PTPI1-PCCW12-MjDOD-Pgossypii:NatMX:tTEF1gossypii | This study |
| **ScBTX020** | BY4741 | MATa his3Δ1 leu2Δ0 met15Δ0 ura3Δ0 **XI-3::KanMX** | This study |
| **ScBTX021** | ScBTX020 | MATa his3Δ1 leu2Δ0 met15Δ0 ura3Δ0 **XI-3::KanMX** XII-5::TADH1-ARO7G141S-PTEF1-PPGK1-ARO4K229L-TTDH3-  BvCYP76AD1-PTPI1-PCCW12-MjDOD-Pgossypii:NatMX:tTEF1gossypii | This study |
| **ScBTX022** | ScBTX004 | MATa his3Δ1 leu2Δ0 met15Δ0 ura3Δ0 hem25**Δ0** XII-5::*TADH1-ARO7G141S-PTEF1-PPGK1-ARO4K229L-TTDH3-*  BvCYP76AD1-PTPI1-PCCW12-MjDOD-Pgossypii:NatMX:tTEF1gossypii | This study |
| **ScBTX024** | ScBTX004 | MATa his3Δ1 leu2Δ0 met15Δ0 ura3Δ0 pro2**Δ0** XII-5::*TADH1-ARO7G141S-PTEF1-PPGK1-ARO4K229L-TTDH3-*  BvCYP76AD1-PTPI1-PCCW12-MjDOD-Pgossypii:NatMX:tTEF1gossypii | This study |
| **ScBTX025** | ScBTX004 | MATa his3Δ1 leu2Δ0 met15Δ0 ura3Δ0 vma16**Δ0** XII-5::*TADH1-ARO7G141S-PTEF1-PPGK1-ARO4K229L-TTDH3-*  BvCYP76AD1-PTPI1-PCCW12-MjDOD-Pgossypii:NatMX:tTEF1gossypii | This study |
| **ScBTX027** | ScBTX004 | MATa his3Δ1 leu2Δ0 met15Δ0 ura3Δ0 get4**Δ0** XII-5::*TADH1-ARO7G141S-PTEF1-PPGK1-ARO4K229L-TTDH3-*  BvCYP76AD1-PTPI1-PCCW12-MjDOD-Pgossypii:NatMX:tTEF1gossypii | This study |
| **ScBTX028** | ScBTX004 | MATa his3Δ1 leu2Δ0 met15Δ0 ura3Δ0 pry1**Δ0** XII-5::*TADH1-ARO7G141S-PTEF1-PPGK1-ARO4K229L-TTDH3-*  BvCYP76AD1-PTPI1-PCCW12-MjDOD-Pgossypii:NatMX:tTEF1gossypii | This study |
| **ScBTX029** | ScBTX004 | MATa his3Δ1 leu2Δ0 met15Δ0 ura3Δ0 stv1**Δ0** XII-5::*TADH1-ARO7G141S-PTEF1-PPGK1-ARO4K229L-TTDH3-*  BvCYP76AD1-PTPI1-PCCW12-MjDOD-Pgossypii:NatMX:tTEF1gossypii | This study |
| **ScBTX030** | ScBTX004 | MATa his3Δ1 leu2Δ0 met15Δ0 ura3Δ0 ycr101C**Δ0** XII-5::*TADH1-ARO7G141S-PTEF1-PPGK1-ARO4K229L-TTDH3-*  BvCYP76AD1-PTPI1-PCCW12-MjDOD-Pgossypii:NatMX:tTEF1gossypii | This study |
| **ScBTX031** | ScBTX004 | MATa his3Δ1 leu2Δ0 met15Δ0 ura3Δ0 YLR271W**Δ0** XII-5::*TADH1-ARO7G141S-PTEF1-PPGK1-ARO4K229L-TTDH3-*  BvCYP76AD1-PTPI1-PCCW12-MjDOD-Pgossypii:NatMX:tTEF1gossypii | This study |
| **ScBTX032** | ScBTX004 | MATa his3Δ1 leu2Δ0 met15Δ0 ura3Δ0 glr1**Δ0** XII-5::*TADH1-ARO7G141S-PTEF1-PPGK1-ARO4K229L-TTDH3-*  BvCYP76AD1-PTPI1-PCCW12-MjDOD-Pgossypii:NatMX:tTEF1gossypii | This study |
| **ScBTX051** | ScBTX004 | MATa his3Δ1 leu2Δ0 met15Δ0 ura3Δ0 qcr10**Δ0** XII-5::*TADH1-ARO7G141S-PTEF1-PPGK1-ARO4K229L-TTDH3-*  BvCYP76AD1-PTPI1-PCCW12-MjDOD-Pgossypii:NatMX:tTEF1gossypii | This study |
| **ScBTX052** | ScBTX004 | MATa his3Δ1 leu2Δ0 met15Δ0 ura3Δ0 ssm4**Δ0** XII-5::*TADH1-ARO7G141S-PTEF1-PPGK1-ARO4K229L-TTDH3-*  BvCYP76AD1-PTPI1-PCCW12-MjDOD-Pgossypii:NatMX:tTEF1gossypii | This study |
| **ScBTX053** | ScBTX004 | MATa his3Δ1 leu2Δ0 met15Δ0 ura3Δ0 ubc7**Δ0** XII-5::*TADH1-ARO7G141S-PTEF1-PPGK1-ARO4K229L-TTDH3-*  BvCYP76AD1-PTPI1-PCCW12-MjDOD-Pgossypii:NatMX:tTEF1gossypii | This study |
| **ScBTX055** | ScBTX004 | MATa his3Δ1 leu2Δ0 met15Δ0 ura3Δ0 rpn4**Δ0** XII-5::*TADH1-ARO7G141S-PTEF1-PPGK1-ARO4K229L-TTDH3-*  BvCYP76AD1-PTPI1-PCCW12-MjDOD-Pgossypii:NatMX:tTEF1gossypii | This study |

**SUPPLEMENTARY TABLE S2: Plasmids Used in this Study**

| **Plasmid Name** | **Genetic Element** | **Yeast Marker** | **Type** |
| --- | --- | --- | --- |
| **pHO29** | pSNR52-gRNA(XII-5)-tSUP4 | hphNT1 | 2μ |
| **pHO-*ADE2*** | pSNR52-gRNA(ADE2)-tSUP4, NatMX | hphNT1 | 2μ |
| **pHO8** | p*TEF1*-*Sp*Cas9-t*CYC1* | *KlLEU2* | Integrative, X-3 |
| **pHO22** | pSNR52-target(ChrXV)-tSUP4-NatMX |  | Integrative downstream of ADE2 |
| **pHO25** | pSNR52-target(downstream XII-5)-tSUP4-NatMX |  | Integrative downstream of XII-5 |
| **pBTX1** | PSNR52-gRNA.XII-5-TSUP4  ARO7G141S-PTEF1-PPGK1-ARO4K229L-TTDH3-  BvCYP76AD1-PTPI1-PCCW12-MjDOD pTEFagossypii::NatMX::tTEFagossypi | NatMX, URA3 | Integrative, XII-5 |
| **pBTX2** | TSUP4ARO7G141S-PTEF1-PPGK1-ARO4K229L-TTDH3-  BvCYP76AD1-PTPI1-PCCW12-MjDOD | NatMX, URA3 | Integrative, XII-5 |
| **pHO25** | pSNR52-gRNA-tSUP4 | NatMX, URA3 | 2μ |
| **pHO29** | gRNA(XII-5)-hphMX (from pMEL12) | hphMX (from pMEL12) | 2μ |
| **pCCM023** | HomologyA::Cloning site:: HomologyB | Amp (E.coli) | pUC |
| **pBTX013** | Gene::Up KanMX Gene:Dwn Gene=**GLR1** | Amp (E.coli) | pUC |
| **pBTX014** | Gene::Up KanMX Gene:Dwn Gene=**HEM25** | Amp (E.coli) | pUC |
| **pBTX016** | Gene::Up KanMX Gene:Dwn Gene= **PRO2** | Amp (E.coli) | pUC |
| **pBTX017** | Gene::Up KanMX Gene:Dwn Gene= **VMA16** | Amp (E.coli) | pUC |
| **pBTX019** | Gene::Up KanMX Gene:Dwn Gene=**GET4** | Amp (E.coli) | pUC |
| **pBTX020** | Gene::Up KanMX Gene:Dwn Gene=**PRY1** | Amp (E.coli) | pUC |
| **pBTX021** | Gene::Up KanMX Gene:Dwn Gene=**STV1** | Amp (E.coli) | pUC |
| **pBTX022** | Gene::Up KanMX Gene:Dwn Gene=**YCR101C** | Amp (E.coli) | pUC |
| **pBTX023** | Gene::Up KanMX Gene:Dwn Gene= **YLR271W** | Amp (E.coli) | pUC |

**SUPPLEMENTARY TABLE S3: Primers Used in this Study**

| **Primers** | **Sequence** | **Note** |
| --- | --- | --- |
| **HOP60** | GTTTTAGAGCUAGAAATAGCAAG | For exlcusion of protospacer sequence in pMEL12 (or any vector with the same pSNR52/crRNA set-up) while adding USER tails enabling new protospacer to be inserted. |
| **HOP61** | GATCATTTAUCTTTCACTGC | For exlcusion of protospacer sequence in pMEL12 (or any vector with the same pSNR52/crRNA set-up) while adding USER tails enabling new protospacer to be inserted. |
| **HOP64** | TATAACAATCAAGAAAAACAAGAAAATCGGACAAAACAATCAAGTGCATAGGCCACTAGTGG | For generating a ade2Δ::hygR deletion cassette from PCR with pMEL12 |
| **HOP65** | ATTTTATAATTATTTGCTGTACAAGTATATCAATAAACTTATATACATCAATAGGCACCTTCG | For generating a ade2Δ::hygR deletion cassette from PCR with pMEL12 |
| **HOP89** | ACACGCGAUGCTGGAGCTCATAGCTTCA | To amplify pTEF1-Cas9-tCYC1 from pCfB2312 while generating USER tails for cloning into pCfB257/259 linearised by HO_P91 and HO_P92. |
| **HOP90** | ACGTGCGAUCTATAGGGCGAATTGGGTAC | To amplify pTEF1-Cas9-tCYC1 from pCfB2312 while generating USER tails for cloning into pCfB257/259 linearised by HO_P91 and HO_P92. |
| **HOP91** | ATCGCGTGUGAGCTCGCTGAGGACTT | Opening up pCfB257 or pCfB259. Excluding tADH1 and tCYC1 from the USER cassette, adding USER tails for cloning of new cassette. |
| HOP92 | ATCGCACGUCAGCTGAAGCTTCGTACG | Opening up pCfB257 or pCfB259. Excluding tADH1 and tCYC1 from the USER cassette, adding USER tails for cloning of new cassette. |
| HOP175 | TGATACTGTTCTCATAAACATGTGACTGCATTGGTGGTTGgggttctcgagagctcg | 40 bp tail corresponding to UP cas9 target site downstrream ADE2 |
| **HOP176** | TCCGAGGGTAACTGTGATAGCTTCAAAAGACTTTTAGCATggtctagagatcccaataca | 40 bp tail corresponding to DW cas9 target site downstream ADE2 |
| **HOP181** | GTATACCTTTCATTTACAAAATGTCAAAGACTTTACACAGGGGTTCTCGAGAGCTCG | To amplify KlURA3 from pCfB390 while adding 40 bp tails for integration at genomic site downstream the XII-5 site. |
| **HOP182** | CGCAATGACATAGCTTAGAGCAGTCAGAAAAGTGATTGTAGGTCTAGAGATCCCAATACA | To amplify KlURA3 from pCfB390 while adding 40 bp tails for integration at genomic site downstream the XII-5 site. |
| **HOP183** | ATTCATGTGATAATTTGACGGTTTTAGAGCT | Cloning gRNA target sequence for downstream XII-5, (+) strand |
| **HOP184** | CGTCAAATTATCACATGAATGATCATTTAT | Cloning gRNA target sequence for downstream XII-5, (+) strand |
| **HOP190** | AATTGTAGAGACTATCCACAGTTTTAGAGCT | Cloning gRNA target sequence for *ADE2*, (+) strand |
| **HOP191** | TGTGGATAGTCTCTACAATTGATCATTTAT | Cloning gRNA target sequence for *ADE2*, (-) strand |
| **HOP217** | ACGTGCGAUTCTCGAGAGCTCGTTAAAGC | hphNT1 cassette amplification from pMEL12 |
| **HOP218** | ACA CGC GAUAGAGATCTGTTTAGCTTGCCTCG | hphNT1 cassette amplification from pMEL12 |
| **HOP258** | ATTCTGCAGGUAGGGAAAGATATGAG | Construction of plasmid pHO36 |
| **HOP259** | ACCTGCAGAAUcgagctcgctgaggac | Construction of plasmid pHO36 |
| **PR_DIV2288** | **CGTGCGAU**TTACTCTTCCAACCTTCTTAGCAAG | Building pBTX2 from pBTX1 excluding the NTC marker |
| **ANT_P494** | **CACGCGAU**TTAGGATCCGTCGGTCTTTTGG | Building pBTX2 from pBTX1 excluding the NTC marker |
| **BTX.006** | gggacgaggcaagctaaacagatctctagacctaGGATCTTAGTTTCGTTTTTAGTAATG | gene KO: COX12 Upper cap: anneals to gene, ‚Ä®Low. cap: homol. to KanMX |
| **BTX.007** | agtgtcgaaaacgagctctcgagaacccttaatGTCTATTATACTGTATTTATGTGCTTG | gene KO: COX12 Upper cap: anneals to gene, ‚Ä®Low. cap: homol. to KanMX |
| **BTX.0103** | tcgaaaacgagctctcgagaacccttaatTTTGATGCGGAAACAACGGG | gene KO: QCR10 Upper cap: anneals to gene, ‚Ä®Low. cap: homol. to KanMX |
| **BTX.0104** | gacgaggcaagctaaacagatctctagacctaCCAAAACATCCAACCGGCT | gene KO: QCR10 Upper cap: anneals to gene, ‚Ä®Low. cap: homol. to KanMX |
| **BTX.014** | ggcggggacgaggcaagctaaacagatctctagacctaCTTGTTATATGCCACTTGTAGT | gene KO: DLD2 Upper cap: anneals to gene, ‚Ä®Low. cap: homol. to KanMX |
| **BTX.015** | gtgtcgaaaacgagctctcgagaacccttaatAAATAAAATGCCAAGTTTTATAGTTATC | gene KO: DLD2 Upper cap: anneals to gene, ‚Ä®Low. cap: homol. to KanMX |
| **BTX.018** | cggcggggacgaggcaagctaaacagatctctagacctaTTTGAAATTTGCAGCCCATAC | gene KO: KGD2 Upper cap: anneals to gene, ‚Ä®Low. cap: homol. to KanMX |
| **BTX.019** | gtgtcgaaaacgagctctcgagaacccttaatTGTTTAAATAATTATCTGAAGTTGTGTC | gene KO: KGD2 Upper cap: anneals to gene, ‚Ä®Low. cap: homol. to KanMX |
| **BTX.022** | ggcggggacgaggcaagctaaacagatctctagacctaGCAAAATCTGCATTAGCTTTCG | gene KO: MIP1 Upper cap: anneals to gene, ‚Ä®Low. cap: homol. to KanMX |
| **BTX.023** | gccatccagtgtcgaaaacgagctctcgagaacccttaatACTTGTCCCTCGTTGCT | gene KO: MIP1 Upper cap: anneals to gene, ‚Ä®Low. cap: homol. to KanMX |
| **BTX.030** | ggggacgaggcaagctaaacagatctctagacctaTTCAAAACTATACTCTCTGTTTAGC | gene KO: RCR2 Upper cap: anneals to gene, ‚Ä®Low. cap: homol. to KanMX |
| **BTX.031** | agtgtcgaaaacgagctctcgagaacccttaatTTTTTCGTTTAATGAATTTATTATCGC | gene KO: RCR2 Upper cap: anneals to gene, ‚Ä®Low. cap: homol. to KanMX |
| **BTX.034** | cgaggcaagctaaacagatctctagacctaCTAGAAATACACTCTTATCTTTAGATTTAG | gene KO: SGM1 Upper cap: anneals to gene, ‚Ä®Low. cap: homol. to KanMX |
| **BTX.035** | catccagtgtcgaaaacgagctctcgagaacccttaatAGCAAAATTGGTATACCACTAC | gene KO: SGM1 Upper cap: anneals to gene, ‚Ä®Low. cap: homol. to KanMX |
| **BTX.038** | cggggacgaggcaagctaaacagatctctagacctaTGCTTTCCTCTTTTATTAAATCCT | gene KO: SHY1 Upper cap: anneals to gene, ‚Ä®Low. cap: homol. to KanMX |
| **BTX.039** | gccatccagtgtcgaaaacgagctctcgagaacccttaatACGGCAAACTCCTCTGC | gene KO: SHY1 Upper cap: anneals to gene, ‚Ä®Low. cap: homol. to KanMX |
| **BTX.042** | ccggcggggacgaggcaagctaaacagatctctagacctaTGTTGGAGTTAAACCCTTCC | gene KO: VPS34 Upper cap: anneals to gene, ‚Ä®Low. cap: homol. to KanMX |
| **BTX.043** | ccatccagtgtcgaaaacgagctctcgagaacccttaatTTGGTTGATAATTGGTGCTTC | gene KO: VPS34 Upper cap: anneals to gene, ‚Ä®Low. cap: homol. to KanMX |
| **BTX.053** | TAGGTCTAGAGATCTGTTTAGC | kanMX |
| **BTX.054** | ATTAAGGGTTCTCGAGAGC | kanMX |
| **BTX.057** | ccggcggggacgaggcaagctaaacagatctctagacctaGTTTGTAGCATCAGCAACG | gene KO: YER084W Upper cap: anneals to gene, ‚Ä®Low. cap: homol. to KanMX |
| **BTX.058** | cagtgtcgaaaacgagctctcgagaacccttaatCTCAGATTTTCAAGAAACAGAATAGC | gene KO: YER084W Upper cap: anneals to gene, ‚Ä®Low. cap: homol. to KanMX |
| **BTX.064** | ATATATTTTATATATTGCCCTCAAAACT | Verification Primer CAX4 |
| **BTX.065** | AAACACGCTCTTGAAATTTTTAAAG | Verification Primer CAX4 |
| **BTX.066** | TCAGGCTGTTGATGAAGGT | Verification Primer COX12 |
| **BTX.067** | CAGATTTAGGGACTGAGGAA | Verification Primer COX12 |
| **BTX.068** | TTCTGATTGTAATATAATGACAAATTTTAATC | Verification Primer CUR1 |
| **BTX.069** | TTCCGTTTTATATGTTTATATTCATTGA | Verification Primer CUR1 |
| **BTX.070** | CAAGGTCTTCTCTACTGGC | Verification Primer DLD2 |
| **BTX.071** | ATGAATCAGAAGTTTTAGTTGATAGAA | Verification Primer DLD2 |
| **BTX.072** | TCAACTATTTAGGATTTTGTAAAGAAAA | Verification Primer KGD2 |
| **BTX.073** | GTGGGACATATTCGAACGT | Verification Primer KGD2 |
| **BTX.074** | TACACATCGCCTCCAGA | Verification Primer MIP1 |
| **BTX.075** | GAATATCCCGTCTAGCGAC | Verification Primer MIP1 |
| **BTX.078** | GAAGCCTTCACTTTTGCTATC | Verification Primer RCR2 |
| **BTX.079** | CATTTTGCCTTACTGCATCC | Verification Primer RCR2 |
| **BTX.080** | GGACAAAAGAATATTCCGATTCA | Verification Primer SGM1 |
| **BTX.081** | AAAACTGCAGCATCAAATGAC | Verification Primer SGM1 |
| **BTX.082** | CTCAAAAAATTACTAAGGAAGGC | Verification Primer SHY1 |
| **BTX.083** | CTCTTATTTTAGGCAACAGAAGC | Verification Primer SHY1 |
| **BTX.084** | AACTCAAACGTTAATATTATCGGA | Verification Primer VPS34 |
| **BTX.085** | CCAGCCGAATAAACTTGAC | Verification Primer VPS34 |
| **BTX.090** | ctgattgagtaaatgcataggc | Verification Primer YER084W |
| **BTX.091** | GAGGGTTTTGAACAGGGG | Verification Primer YER084W |
| **BTX.102** | CATTTTGGAAACTGCCCCGA | Verification Primer QCR10 |
| **BTX.105** | CATAGAGATGTGCTGCCCAA | gene KO: QCR10 Upper cap: anneals to gene, ‚Ä®Low. cap: homol. to KanMX |
| **BTX.105** | CATAGAGATGTGCTGCCCAA | Verification Primer QCR10 |
| **BTX.116** | AAGAGGGCuATTTCACTTCGTCCTTAATATTTTAAC | USER primer HEM25_up |
| **BTX.117** | CGTGCGAuATGCAAATTTAAATGTAATACGGTA | USER primer HEM25_up |
| **BTX.118** | CACGCGAuGTGCACTTATCCATCGACT | USER primer HEM25_dw |
| **BTX.119** | ACACAGGCuAAGGTATAAATACGTGTCTTTTTAGT | USER primer HEM25_dw |
| **BTX.120** | CGTGCGAuGGCCTATTGGGTATCTGC | USER primer VMA16_up |
| **BTX.121** | AAGAGGGCuAACACAGTATACTAGGGGTA | USER primer VMA16_up |
| **BTX.122** | ACACAGGCuAGCGCATCCATATTTACTAATAG | USER primer VMA16_dw |
| **BTX.123** | CACGCGAuACTGAATAGCTTTTGGGCA | USER primer VMA16_dw |
| **BTX.128** | AAGAGGGCuTTGTAAAGTGACCTTGTTTTGAC | USER primer PRO2_up |
| **BTX.129** | CGTGCGAuCTAAGGGAAATGGATAGTTTGA | USER primer PRO2_up |
| **BTX.130** | CACGCGAuGAGTAGATGTAAGACGTGAAAATG | USER primer PRO2_dw |
| **BTX.131** | ACACAGGCuTCCAATTAGCTTTCAGTTTTCC | USER primer PRO2_dw |
| **BTX.132** | CGTGCGAuTGGTCAATTCTATGCCTTTTATG | USER primer GLR1_up |
| **BTX.132** | CGTGCGAuTGGTCAATTCTATGCCTTTTATG | USER primer GLR1_up |
| **BTX.133** | AAGAGGGCuTACGTATAAAACCAACAATTTTATGC | USER primer GLR1_up |
| **BTX.133** | AAGAGGGCuTACGTATAAAACCAACAATTTTATGC | USER primer GLR1_up |
| **BTX.134** | ACACAGGCuCTTTTACTGCTTTCTAACTTTAAGC | USER primer GLR1_dw |
| **BTX.134** | ACACAGGCuCTTTTACTGCTTTCTAACTTTAAGC | USER primer GLR1_dw |
| **BTX.135** | CACGCGAuGATTGGTTACTCCTCAAAGATTG | USER primer GLR1_dw |
| **BTX.135** | CACGCGAuGATTGGTTACTCCTCAAAGATTG | USER primer GLR1_dw |
| **BTX.149** | TATACCTTCCACAAAGGTTTTT | Verification Primer GLR1 |
| **BTX.150** | TGTTGGTCCAGATTTGGC | Verification Primer GLR1 |
| **BTX.151** | CCAAATAAGCAAATTTGGTTGG | Verification Primer HEM25 |
| **BTX.152** | CTCGAATTTAATCTCTCCTCTAATAG | Verification Primer HEM25 |
| **BTX.155** | CAGTAAAAAGTCCACTTAGCAC | Verification Primer PRO2 |
| **BTX.156** | GATCTTCTATCAGGAGAGTTAACC | Verification Primer PRO2 |
| **BTX.157** | GCCGTCTTGACCACAG | Verification Primer VMA16 |
| **BTX.158** | GATGAGGGAGAGCCAATAA | Verification Primer VMA16 |
| **BTX.161** | CGTGCGAuTTCTAGTATCTACACTGAAGTCAA | USER primer PRY1_up |
| **BTX.162** | AAGAGGGCuTTGTGCTGGAGGGCT | USER primer PRY1_up |
| **BTX.163** | ACACAGGCuGAAAAATTTTCGCTCATTTTCGC | USER primer PRY1_dw |
| **BTX.164** | CACGCGAuTGCTGTTGTGTACTTAGACG | USER primer PRY1_dw |
| **BTX.165** | CGTGCGAuAAAGTATGTCAACCGTCGAG | USER primer GET4_up |
| **BTX.166** | AAGAGGGCuTGATGTTTACTTGGTTGTTTTACC | USER primer GET4_up |
| **BTX.167** | ACACAGGCuAAAGAGCATATTTGCGAAGGA | USER primer GET4_dw |
| **BTX.168** | CACGCGAuAGACATAGAAAGCTATACTCTTATAC | USER primer GET4_dw |
| **BTX.169** | CGTGCGAuGGTTGTTAAAATTCTCACATCATCC | USER primer YLR271W_up |
| **BTX.170** | AAGAGGGCuTAATTGTTTCATTCTTAGTCTTTAATTTATTT | USER primer YLR271W_up |
| **BTX.171** | ACACAGGCuGATGATTTTCTTGAAACTCTCCTTA | USER primer YLR271W_up |
| **BTX.172** | CACGCGAuGACAGGTTAAGGTTAAAGGAC | USER primer YLR271W_up |
| **BTX.173** | CGTGCGAuTACCTATAATAGCGGCTCG | USER primer YCR101C_up |
| **BTX.174** | AAGAGGGCuACCACAGAAAATGGTACAAGA | USER primer YCR101C_up |
| **BTX.175** | ACACAGGCuAATGATACTTCGATACTCTGTTTGT | USER primer YCR101C_dw |
| **BTX.176** | CACGCGAuTGCCCGTCATCTTGCG | USER primer YCR101C_dw |
| **BTX.177** | CGTGCGAuTATTAATAGGTGCTTCTTCCAGG | USER primer STV1_up |
| **BTX.178** | AAGAGGGCuCACCTTCGTGGGCC | USER primer STV1_up |
| **BTX.179** | ACACAGGCuCACATAGTTTACGGTATATATATGTC | USER primer STV1_dw |
| **BTX.180** | CACGCGAuGTGGCATTCCTAGTTAAAATGA | USER primer STV1_dw |
| **BTX.181** | AACAATCTCGACATCTTGTAGC | Verification Primer PRY1 |
| **BTX.182** | GGCGAATCAATGGTAGTTAC | Verification Primer PRY1 |
| **BTX.183** | CCACTCGATTGAGAACCG | Verification Primer GET4 |
| **BTX.184** | CCCAACGAGAAACTTGGG | Verification Primer GET4 |
| **BTX.185** | GACCAGAAGGCTACATGAA | Verification Primer CMG1 |
| **BTX.186** | TTATCATCAAAATATTGTGAAAAGAGC | Verification Primer CMG1 |
| **BTX.187** | TATATCCTTACTATCTACTATCCAAC | Verification Primer YCR101C |
| **BTX.188** | CATTCAACAGGTGAGAATTTCAA | Verification Primer YCR101C |
| **BTX.189** | TTTGCATCTTTTTGTCCACG | Verification Primer STV1 |
| **BTX.190** | ACTAAGAACCTAAACGGCG | Verification Primer STV1 |
| **BTX.200** | agagtaccactaattgaatcaaagagactagaagtgtgaaagtcGCAGTCGAGCTCATGC | gene KO: SSM4 repair up Upper cap: anneals to gene, ‚Ä®Low. cap: homol. to KanMX |
| **BTX.201** | taaatatgctagcattcattttaaatgtaaggaagaaaacgcctGCATGAGCTCGACTGC | gene KO: SSM4 repair dw Upper cap: anneals to gene, ‚Ä®Low. cap: homol. to KanMX |
| **BTX.202** | aaaAGGAACTTCCCTAGTAATAGTGTAATTTGGAAGGGCATAGCGCAGTCGAGCTCATGC | gene KO: UBC7 repair up Upper cap: anneals to gene, ‚Ä®Low. cap: homol. to KanMX |
| **BTX.203** | cagttaaaaggaagaccaaatgatcattaacctgctacctgcttGCATGAGCTCGACTGC | gene KO: UBC7 repair dw Upper cap: anneals to gene, ‚Ä®Low. cap: homol. to KanMX |
| **BTX.205** | gcatcgattgaggacattg | Verification Primer SSM4 |
| **BTX.206** | ggatcaaatgcacaacttctc | Verification Primer SSM4 |

**SUPPLEMENTARY TABLE S4: Reversed Engineered Hits**

| **Gene KO** | **Top/Bottom Hit** | **Normalised Fitness CRI-SPA score** | **Normalised Yellowness CRI-SPA score** | **SGD annotation** |
| --- | --- | --- | --- | --- |
| **WT** | NA | 0.35 | 0.22 | Betaxanthin making control strain, BY4741. Parent to all KOs |
| **SHY1** | Top | -0.82 | 2.25 | Mitochondrial inner membrane protein required for complex IV assembly |
| **DLD2** | Top | 0.82 | 0.52 | D-2-hydroxyglutarate dehydrogenase, and minor D-lactate dehydrogenase; located in the mitochondrial matrix |
| **MIP1** | Top | -1.23 | 1.51 | Mitochondrial DNA polymerase gamma; single subunit of mitochondrial DNA polymerase in yeast |
| **RCR2** | Top | 0.85 | 0.94 | Vacuolar protein; presumably functions within the endosomal-vacuolar trafficking pathway, |
| **SGM1** | Top | 0.65 | 0.65 | hypothetical protein; required for wild-type growth rate on galactose and mannose; localizes to COPI coated vesicles and the Golgi apparatus |
| **YER084W** | Top | 0.0 | 1.19 | hypothetical protein; expressed at both mRNA and protein levels, mitochondrial protein |
| **COX12** | Top | -0.75 | 1.54 | Subunit VIb of cytochrome c oxidase; cytochrome c oxidase is also known as respiratory Complex IV and is the terminal member of the mitochondrial inner membrane electron transport chain; required for assembly of cytochrome c oxidase but not required for activity after assembly; |
| **GET4** | Top | 0.3 | 0.76 | Protein involved in inserting tail-anchored proteins into ER membranes; |
| **PRY1** | Top | 0.2 | 1.01 | Sterol binding protein involved in the export of acetylated sterols; |
| **STV1** | Top | 0.8 | 0.76 | Subunit a of the vacuolar-ATPase V0 domain; one of two isoforms (Stv1p and Vph1p); Stv1p is located in V-ATPase complexes of the Golgi and endosomes |
| **YCR101C** | Top | 0.61 | 0.59 | hypothetical protein; localizes to the membrane fraction; |
| **YLR271W** | Top | 1.29 | 0.93 | hypothetical protein; green fluorescent protein (GFP)-fusion protein localizes to the cytoplasm and the nucleus |
| **QCR10** | Top | 1.63 | 0.25 | Subunit of the ubiqunol-cytochrome c oxidoreductase complex; this complex comprises part of the mitochondrial respiratory chain |
| **SSM4** | Top | 0.13 | 1.92 | Membrane-embedded ubiquitin-protein ligase and retrotranslocase; ER and inner nuclear membrane localized RING-CH domain E3 ligase involved in ER-associated protein degradation (ERAD); |
| **UBC7** | Top | -0.38 | 2.7 | Ubiquitin conjugating enzyme; involved in the ER-associated protein degradation (ERAD) pathway and in the inner nuclear membrane-associated degradation (INMAD) pathway; |
| **RPN4** | Top | -0.15 | 1.15 | Transcription factor that stimulates expression of proteasome genes |
| **GLR1** | Bottom | 0.2 | -0.99 | Cytosolic and mitochondrial glutathione oxidoreductase; converts oxidized glutathione to reduced glutathione; |
| **HEM25** | Bottom | -0.83 | -1.65 | Mitochondrial glycine transporter; required for the transport of glycine into mitochondria for initiation of heme biosynthesis, |
| **PRO2** | Bottom | -1.92 | -1.8 | Gamma-glutamyl phosphate reductase; catalyzes the second step in proline biosynthesis |
| **VMA16** | Bottom | -2.08 | -1.57 | Subunit c'' of the vacuolar ATPase; v-ATPase functions in acidification of the vacuole |
| **VPS34** | Bottom | -1.39 | -2.05 | Phosphatidylinositol (PI) 3-kinase that synthesizes PI-3-phosphate; forms membrane-associated signal transduction complex with Vps15p to regulate protein sorting; |
| **KGD2** | Bottom | -0.23 | -1.71 | component of the mitochondrial alpha-ketoglutarate dehydrogenase complex, which catalyzes the oxidative decarboxylation of alpha-ketoglutarate to succinyl-CoA in the TCA cycle; phosphorylated |

**SUPPLEMENTARY TABLE S5: Top Betaxanthin Hits**

| **gene** | **Yield_Rank** | **Yield_Score** | **Size_Score** | **Description** |
| --- | --- | --- | --- | --- |
| **UBC7** | 1 | 3.042 | -0.497 | Ubiquitin conjugating enzyme; involved in the ER-associated protein degradation (ERAD) pathway and in the inner nuclear membrane-associated degradation (INMAD) pathway; requires Cue1p for recruitment to the ER membrane; proposed to be involved in chromatin assembly |
| **COQ2** | 2 | 2.972 | -0.675 | Para hydroxybenzoate polyprenyl transferase; catalyzes the second step in ubiquinone (coenzyme Q) biosynthesis; human COQ2, mutations in which are implicated in an increased risk of mutiple-system atrophy, can complement a yeast coq2 null mutant |
| **CBP6** | 3 | 2.904 | -0.48 | Mitochondrial protein required for translation of the COB mRNA; forms a complex with Cbp3p that binds to mt ribosomes near the polypeptide tunnel exit and promotes efficient translation of the COB mRNA; Cbp3p-Cbp6p complex also interacts with newly synthesized cytochrome b (Cobp) and Cbp4p to promote assembly of Cobp into the cytochrome bc1 complex; Cbp3p-Cbp6p complex is sequestered if assembly of Complex III is blocked, downregulating COB mRNA translation |
| **SHY1** | 4 | 2.869 | -0.827 | Mitochondrial inner membrane protein required for complex IV assembly; associates with complex IV assembly intermediates and complex III/complex IV supercomplexes; similar to human SURF1 involved in Leigh Syndrome; complex IV is also known as cytochrome c oxidase |
| **PET100** | 5 | 2.791 | -0.886 | Chaperone that facilitates the assembly of cytochrome c oxidase; integral to the mitochondrial inner membrane; interacts with a subcomplex of subunits VII, VIIa, and VIII (Cox7p, Cox9p, and Cox8p) but not with the holoenzyme |
| **SEC28** | 6 | 2.764 | -0.426 | Epsilon-COP subunit of the coatomer; regulates retrograde Golgi-to-ER protein traffic; stabilizes Cop1p, the alpha-COP and the coatomer complex; non-essential for cell growth; protein abundance increases in response to DNA replication stress |
| **MRPL7** | 7 | 2.717 | -0.55 | Mitochondrial ribosomal protein of the large subunit; MRPL7 produces both YmL5 and YmL7, which are two different modified forms of the same protein |
| **MRM1** | 8 | 2.636 | -1.035 | Ribose methyltransferase; modifies a functionally critical, conserved nucleotide in mitochondrial 21S rRNA |
| **MTG2** | 9 | 2.632 | -0.86 | Putative GTPase; member of the Obg family; peripheral protein of the mitochondrial inner membrane that associates with the large ribosomal subunit; required for mitochondrial translation, possibly via a role in ribosome assembly |
| **PET54** | 10 | 2.629 | -0.689 | Mitochondrial inner membrane protein; binds to the 5' UTR of the COX3 mRNA to activate its translation together with Pet122p and Pet494p; also binds to the COX1 Group I intron AI5 beta to facilitate exon ligation during splicing |
| **SSM4** | 11 | 2.619 | 0.037 | Membrane-embedded ubiquitin-protein ligase; ER and inner nuclear membrane localized RING-CH domain E3 ligase involved in ER-associated protein degradation (ERAD); targets misfolded cytosolic/nucleoplasmic domains of soluble and membrane embedded proteins (ERAD-C) and a transmembrane domain containing substrate (ERAD-M), Sbh2p; C-terminal element (CTE), conserved in human ortholog MARCH10/TEB4, determines substrate selectivity |
| **COQ5** | 12 | 2.619 | -0.893 | 2-hexaprenyl-6-methoxy-1,4-benzoquinone methyltransferase; involved in ubiquinone (Coenzyme Q) biosynthesis; localizes to the matrix face of the mitochondrial inner membrane in a large complex with other ubiquinone biosynthetic enzymes; respiratory defect of the null mutant is partially complemented by human COQ5 |
| **COQ10** | 13 | 2.61 | -0.327 | Coenzyme Q (ubiquinone) binding protein; functions in the delivery of Q6 to its proper location for electron transport during respiration; START domain protein with homologs in bacteria and eukaryotes; respiratory growth defect of the null mutant is functionally complemented by human COQ10A |
| **CBS2** | 14 | 2.61 | -1.409 | Mitochondrial translational activator of the COB mRNA; interacts with translating ribosomes, acts on the COB mRNA 5'-untranslated leader |
| **PET111** | 15 | 2.608 | -0.319 | Mitochondrial translational activator specific for the COX2 mRNA; located in the mitochondrial inner membrane |
| **ABF2** | 16 | 2.603 | -0.512 | Mitochondrial DNA-binding protein; involved in mitochondrial DNA replication and recombination, member of HMG1 DNA-binding protein family; activity may be regulated by protein kinase A phosphorylation; ABF2 has a paralog, IXR1, that arose from the whole genome duplication; human homolog TFAM can complement yeast abf2 mutant, rescuing the loss-of-mitochondrial DNA phenotype in a yeast abf2 strain |
| **MSS2** | 17 | 2.586 | -0.681 | Peripherally bound inner membrane protein of the mitochondrial matrix; involved in membrane insertion of C-terminus of Cox2p, interacts genetically and physically with Cox18p |
| **PET494** | 18 | 2.577 | -1.028 | Mitochondrial translational activator specific for the COX3 mRNA; acts together with Pet54p and Pet122p; located in the mitochondrial inner membrane |
| **SCO1** | 19 | 2.576 | -0.884 | Copper-binding protein of mitochondrial inner membrane; required for cytochrome c oxidase activity and respiration; may function to deliver copper to cytochrome c oxidase; similar to thioredoxins; SCO1 has a paralog, SCO2, that arose from the whole genome duplication |
| **RRG8** | 20 | 2.545 | -1.354 | hypothetical protein; required for mitochondrial genome maintenance; null mutation results in a decrease in plasma membrane electron transport |
| **MRPL51** | 21 | 2.537 | -1.363 | Mitochondrial ribosomal protein of the large subunit |
| **CBP2** | 22 | 2.533 | -0.858 | Required for splicing of the group I intron bI5 of the COB pre-mRNA; nuclear-encoded mitochondrial protein that binds to the RNA to promote splicing; also involved in but not essential for splicing of the COB bI2 intron and the intron in the 21S rRNA gene |
| **CYC3** | 23 | 2.505 | -0.656 | Cytochrome c heme lyase (holocytochrome c synthase); attaches heme to apo-cytochrome c (Cyc1p or Cyc7p) in mitochondrial intermembrane space; human homolog HCCS implicated in microphthalmia with linear skin defects (MLS), and can complement yeast null mutant |
| **IRC19** | 24 | 2.505 | -0.827 | hypothetical protein; YLL033W is not an essential gene but mutant is defective in spore formation; null mutant displays increased levels of spontaneous Rad52p foci |
| **DIA4** | 25 | 2.504 | -0.491 | Probable mitochondrial seryl-tRNA synthetase; mutant displays increased invasive and pseudohyphal growth |
| **MNE1** | 26 | 2.484 | -0.522 | Protein involved in splicing Group I aI5-beta intron from COX1 mRNA; mitochondrial matrix protein |
| **MRH4** | 27 | 2.466 | -0.769 | Mitochondrial ATP-dependent RNA helicase of the DEAD-box family; required for assembly of the large subunit of mitochondrial ribosomes; binds to the large subunit rRNA, 21S_rRNA; localizes to the matrix face of the mitochondrial inner membrane and associates with the large subunit precursor and with mature ribosomes |
| **IFM1** | 28 | 2.453 | -0.497 | Mitochondrial translation initiation factor 2 |
| **QCR7** | 29 | 2.45 | -0.566 | Subunit 7 of ubiquinol cytochrome-c reductase (Complex III); Complex III is a component of the mitochondrial inner membrane electron transport chain; oriented facing the mitochondrial matrix; N-terminus appears to play a role in complex assembly |
| **MRPL8** | 30 | 2.449 | -0.629 | Mitochondrial ribosomal protein of the large subunit |
| **MLS1** | 31 | 2.437 | -0.677 | Malate synthase, enzyme of the glyoxylate cycle; involved in utilization of non-fermentable carbon sources; expression is subject to carbon catabolite repression; localizes in peroxisomes during growth on oleic acid, otherwise cytosolic; can accept butyryl-CoA as acyl-CoA donor in addition to traditional substrate acetyl-CoA |
| **CUE3** | 32 | 2.429 | -0.428 | hypothetical protein; has a CUE domain that binds ubiquitin, which may facilitate intramolecular monoubiquitination |
| **SLS1** | 33 | 2.423 | -0.696 | Mitochondrial membrane protein; coordinates expression of mitochondrially-encoded genes; may facilitate delivery of mRNA to membrane-bound translation machinery |
| **PET130** | 34 | 2.404 | -0.807 | Protein required for respiratory growth; the authentic, non-tagged protein is detected in highly purified mitochondria in high-throughput studies |
| **COX18** | 35 | 2.387 | -0.9 | Protein required for membrane insertion of C-terminus of Cox2p; mitochondrial integral inner membrane protein; interacts genetically and physically with Mss2p and Pnt1p; similar to S. cerevisiae Oxa1, N. crassa Oxa2p, and E. coli YidC; respiratory defect of the null mutant is functionally complemented by human COX18 carrying the N-terminal 54 amino acids of S. cerevisiae Cox18p |
| **COX23** | 36 | 2.386 | -0.649 | Protein that functions in mitochondrial copper homeostasis; mitochondrial intermembrane space protein; essential for functional cytochrome oxidase expression; homologous to Cox17p; contains twin cysteine-x9-cysteine motifs |
| **HAP2** | 37 | 2.383 | -0.38 | Subunit of the Hap2p/3p/4p/5p CCAAT-binding complex; complex is heme-activated and glucose-repressed; complex is a transcriptional activator and global regulator of respiratory gene expression; contains sequences sufficient for both complex assembly and DNA binding; respiratory defect of the null mutant is functionally complemented by human NFYA |
| **COQ3** | 38 | 2.382 | -0.695 | O-methyltransferase; catalyzes two different O-methylation steps in ubiquinone (Coenzyme Q) biosynthesis; component of a mitochondrial ubiquinone-synthesizing complex; phosphoprotein |
| **COQ4** | 39 | 2.379 | -0.98 | Protein with a role in ubiquinone (Coenzyme Q) biosynthesis; possibly functioning in stabilization of Coq7p; located on matrix face of mitochondrial inner membrane; component of a mitochondrial ubiquinone-synthesizing complex; human homolog COQ4 can complement yeast coq4 null mutant |
| **PET112** | 40 | 2.375 | -1.213 | Subunit of the trimeric GatFAB AmidoTransferase(AdT) complex; involved in the formation of Q-tRNAQ; mutation is functionally complemented by the bacterial GatB ortholog |
| **MSR1** | 41 | 2.362 | -1.131 | Mitochondrial arginyl-tRNA synthetase; mutations in human ortholog are associated with pontocerebellar hypoplasia type 6; MSR1 has a paralog, YDR341C, that arose from the whole genome duplication |
| **COQ6** | 42 | 2.359 | -0.619 | Flavin-dependent monooxygenase involved in ubiquinone biosynthesis; responsible for hydroxylation at position C5 and deamination at C4 during ubiquinone (Coenzyme Q) biosynthesis; localizes to matrix face of mitochondrial inner membrane in a large complex with other ubiquinone biosynthetic enzymes; human homolog COQ6 can complement yeast null mutant and is implicated in steroid-resistant nephrotic syndrome (SRNS) |
| **CCM1** | 43 | 2.357 | -0.696 | Mitochondrial 15S rRNA-binding protein; required for intron removal of COB and COX1 pre-mRNAs; has separable roles in stabilizing mitochondrial 15S rRNA and in maturation of the COB and COX1 mRNAs; contains pentatricopeptide repeat (PPR) motifs; mutant is respiratory deficient and has defective plasma membrane electron transport |
| **COX19** | 44 | 2.353 | -0.773 | Protein required for cytochrome c oxidase assembly; located in the cytosol and mitochondrial intermembrane space; putative copper metallochaperone that delivers copper to cytochrome c oxidase; contains twin cysteine-x9-cysteine motifs |
| **CUE1** | 45 | 2.348 | -0.202 | Ubiquitin-binding protein; ER membrane protein that recruits and integrates the ubiquitin-conjugating enzyme Ubc7p into ER membrane-bound ubiquitin ligase complexes that function in the ER-associated degradation (ERAD) pathway for misfolded proteins; contains a CUE domain that binds ubiquitin to facilitate intramolecular monoubiquitination and to promote diubiquitin elongation, facilitating polyubiquitin chain formation |
| **MRPL32** | 46 | 2.343 | -1.177 | Mitochondrial ribosomal protein of the large subunit; protein abundance increases in response to DNA replication stress |
| **CYT2** | 47 | 2.337 | -0.941 | Cytochrome c1 heme lyase; involved in maturation of cytochrome c1, which is a subunit of the mitochondrial ubiquinol-cytochrome-c reductase; links heme covalently to apocytochrome c1; human homolog HCCS can complement yeast cyt2 null mutant |
| **MRPL38** | 48 | 2.335 | -1.015 | Mitochondrial ribosomal protein of the large subunit; appears as two protein spots (YmL34 and YmL38) on two-dimensional SDS gels; protein abundance increases in response to DNA replication stress |
| **MSF1** | 49 | 2.326 | -0.985 | Mitochondrial phenylalanyl-tRNA synthetase; active as a monomer, unlike the cytoplasmic subunit which is active as a dimer complexed to a beta subunit dimer; similar to the alpha subunit of E. coli phenylalanyl-tRNA synthetase |
| **EXO5** | 50 | 2.326 | -0.697 | Mitochondrial 5'-3' exonuclease and sliding exonuclease; required for mitochondrial genome maintenance; distantly related to the RecB nuclease domain of bacterial RecBCD recombinases; may be regulated by the transcription factor Ace2 |
| **MEF2** | 51 | 2.321 | -0.801 | Mitochondrial elongation factor involved in translational elongation |
| **CBT1** | 52 | 2.314 | -0.529 | Protein involved in 5' RNA end processing; substrates include mitochondrial COB, 15S_rRNA, and RPM1 transcripts; may also have a role in 3' end processing of the COB pre-mRNA; displays genetic interaction with cell cycle-regulated kinase Dbf2p |
| **YER140W** | 53 | 2.311 | -1.029 | Integral membrane protein of the ER; forms an ER-membrane associated protein complex with Slp1p; identified along with SLP1 in a screen for mutants defective in the unfolded protein response (UPR); proposed to function in the folding of integral membrane proteins; interacts genetically with MPS3; the authentic, non-tagged protein is detected in highly purified mitochondria in high-throughput studies |
| **CAT5** | 54 | 2.302 | -1.198 | Protein required for ubiquinone (Coenzyme Q) biosynthesis; localizes to the matrix face of the mitochondrial inner membrane in a large complex with ubiquinone biosynthetic enzymes; required for gluconeogenic gene activation |
| **MIP1** | 55 | 2.301 | -1.267 | Mitochondrial DNA polymerase gamma; single subunit of mitochondrial DNA polymerase in yeast, in contrast to metazoan complex of catalytic and accessory subunits; polymorphic in yeast, petites occur more frequently in some lab strains; human ortholog POLG complements yeast mip1 mutant; mutations in human POLG associated with Alpers-Huttenlocher syndrome (AHS), progressive external ophthalmoplegia (PEO), parkinsonism, other mitochondrial diseases |
| **MRPL17** | 56 | 2.295 | -1.013 | Mitochondrial ribosomal protein of the large subunit |
| **TDH1** | 57 | 2.283 | -0.565 | Glyceraldehyde-3-phosphate dehydrogenase (GAPDH), isozyme 1; involved in glycolysis and gluconeogenesis; tetramer that catalyzes the reaction of glyceraldehyde-3-phosphate to 1,3 bis-phosphoglycerate; detected in the cytoplasm and cell wall; protein abundance increases in response to DNA replication stress; GAPDH-derived antimicrobial peptides secreted by S. cerevisiae are active against a wide variety of wine-related yeasts and bateria |
| **SLM3** | 58 | 2.283 | -0.83 | tRNA-specific 2-thiouridylase; responsible for 2-thiolation of the wobble base of mitochondrial tRNAs; human homolog TRMU is implicated in myoclonus epilepsy associated with ragged red fibers (MERRF), and can complement yeast null mutant |
| **SHM1** | 60 | 2.268 | -1.105 | Mitochondrial serine hydroxymethyltransferase; converts serine to glycine plus 5,10 methylenetetrahydrofolate; involved in generating precursors for purine, pyrimidine, amino acid, and lipid biosynthesis; reverse reaction generates serine |
| **MRP20** | 59 | 2.268 | -1.52 | Mitochondrial ribosomal protein of the large subunit |
| **RRG9** | 61 | 2.266 | -1.321 | hypothetical protein; null mutant lacks mitochondrial DNA and cannot grow on glycerol; the authentic, non-tagged protein is detected in highly purified mitochondria in high-throughput studies |
| **MRPS5** | 62 | 2.261 | -1.451 | Mitochondrial ribosomal protein of the small subunit |
| **COQ9** | 63 | 2.252 | -1.131 | Protein required for ubiquinone biosynthesis and respiratory growth; localizes to matrix face of mitochondrial inner membrane in a large complex with ubiquinone biosynthetic enzymes; ubiquinone is also known as coenzyme Q; human homolog COQ9 can complement yeast coq9 null mutant |
| **AMS1** | 64 | 2.244 | -0.932 | Vacuolar alpha mannosidase; involved in free oligosaccharide (fOS) degradation; delivered to the vacuole in a novel pathway separate from the secretory pathway |
| **PIM1** | 65 | 2.226 | -1.184 | ATP-dependent Lon protease; involved in degradation of misfolded proteins in mitochondria; required for biogenesis and maintenance of mitochondria |
| **MRPL9** | 66 | 2.225 | -1.554 | Mitochondrial ribosomal protein of the large subunit |
| **COR1** | 67 | 2.214 | -1.059 | Core subunit of the ubiquinol-cytochrome c reductase complex; the ubiquinol-cytochrome c reductase complex (bc1 complex) is a component of the mitochondrial inner membrane electron transport chain |
| **CBS1** | 68 | 2.212 | -0.634 | Mitochondrial translational activator of the COB mRNA; membrane protein that interacts with translating ribosomes, acts on the COB mRNA 5'-untranslated leader |
| **MRPL11** | 70 | 2.207 | -1.275 | Mitochondrial ribosomal protein of the large subunit; localizes to vacuole in response to H2O2 |
| **HER2** | 69 | 2.207 | -1.548 | Subunit of the trimeric GatFAB AmidoTransferase(AdT) complex; involved in the formation of Q-tRNAQ; required for remodeling of ER caused by Hmg2p overexpression; similar to bacterial GatA glutamyl-tRNA amidotransferase |
| **MRPL23** | 71 | 2.187 | -1.071 | Mitochondrial ribosomal protein of the large subunit; localizes to vacuole in response to H2O2 |
| **QCR2** | 72 | 2.176 | -0.7 | Subunit 2 of ubiquinol cytochrome-c reductase (Complex III); Complex III is a component of the mitochondrial inner membrane electron transport chain; phosphorylated; transcription is regulated by Hap1p, Hap2p/Hap3p, and heme |
| **MRP51** | 73 | 2.175 | -1.058 | Mitochondrial ribosomal protein of the small subunit; MRP51 exhibits genetic interactions with mutations in the COX2 and COX3 mRNA 5'-untranslated leader sequences |
| **MTF2** | 74 | 2.174 | -0.928 | Mitochondrial protein that interacts with mitochondrial RNA polymerase; interacts with an N-terminal region of mitochondrial RNA polymerase (Rpo41p) and couples RNA processing and translation to transcription |
| **QRI5** | 76 | 2.166 | -0.872 | Mitochondrial inner membrane protein; required for accumulation of spliced COX1 mRNA; may have an additional role in translation of COX1 mRNA |
| **COX6** | 75 | 2.166 | -0.797 | Subunit VI of cytochrome c oxidase (Complex IV); Complex IV is the terminal member of the mitochondrial inner membrane electron transport chain; expression is regulated by oxygen levels |
| **PET117** | 77 | 2.164 | -0.752 | Protein required for assembly of cytochrome c oxidase |
| **COX7** | 78 | 2.149 | -0.525 | Subunit VII of cytochrome c oxidase (Complex IV); Complex IV is the terminal member of the mitochondrial inner membrane electron transport chain |
| **RSM24** | 79 | 2.138 | -1.767 | Mitochondrial ribosomal protein of the small subunit |
| **MRPL3** | 80 | 2.131 | -0.456 | Mitochondrial ribosomal protein of the large subunit; located in close proximity to the polypeptide exit channel of the ribosome; mutations in human homolog MRPL44 cause childhood cardiomyopathy; human MRPL44 deficiency results in inefficient assembly of the mitochondrial ribosome, and in tissue-specific respiratory chain deficiency, manifesting as either Complex I+Complex IV or Complex IV deficiency, depending on a cell type |
| **AIM10** | 81 | 2.117 | -1.495 | Protein with similarity to tRNA synthetases; non-tagged protein is detected in purified mitochondria; null mutant is viable and displays elevated frequency of mitochondrial genome loss |
| **MSM1** | 82 | 2.107 | -0.774 | Mitochondrial methionyl-tRNA synthetase (MetRS); functions as a monomer in mitochondrial protein synthesis; functions similarly to cytoplasmic MetRS although the cytoplasmic form contains a zinc-binding domain not found in Msm1p |
| **PET123** | 83 | 2.093 | -1.199 | Mitochondrial ribosomal protein of the small subunit; PET123 exhibits genetic interactions with PET122, which encodes a COX3 mRNA-specific translational activator |
| **MSD1** | 84 | 2.087 | -0.755 | Mitochondrial aspartyl-tRNA synthetase; required for acylation of aspartyl-tRNA; yeast and bacterial aspartyl-, asparaginyl-, and lysyl-tRNA synthetases contain regions with high sequence similarity, suggesting a common ancestral gene |
| **CBP4** | 85 | 2.07 | 0.02 | Mitochondrial protein required for assembly of cytochrome bc1 complex; interacts with the Cbp3p-Cbp6p complex and newly synthesized cytochrome b (Cobp) to promote assembly of Cobp into the cytochrome bc1 complex |
| **CYT1** | 86 | 2.068 | -1.16 | Cytochrome c1; component of the mitochondrial respiratory chain; expression is regulated by the heme-activated, glucose-repressed Hap2p/3p/4p/5p CCAAT-binding complex |
| **MRPL13** | 87 | 2.066 | -1.258 | Mitochondrial ribosomal protein of the large subunit; not essential for mitochondrial translation |
| **MSW1** | 88 | 2.063 | -1.232 | Mitochondrial tryptophanyl-tRNA synthetase |
| **HAP5** | 89 | 2.054 | -0.327 | Subunit of the Hap2p/3p/4p/5p CCAAT-binding complex; complex is heme-activated and glucose repressed; complex is a transcriptional activator and global regulator of respiratory gene expression; required for assembly and DNA binding activity of the complex |
| **ATP11** | 90 | 2.052 | -0.832 | Molecular chaperone; required for the assembly of alpha and beta subunits into the F1 sector of mitochondrial F1F0 ATP synthase; N-terminally propionylated in vivo |
| **OCT1** | 91 | 2.045 | -1.439 | Mitochondrial intermediate peptidase; cleaves destabilizing N-terminal residues of a subset of proteins upon import, after their cleavage by mitochondrial processing peptidase (Mas1p-Mas2p); may contribute to mitochondrial iron homeostasis |
| **QCR8** | 92 | 2.041 | -1.648 | Subunit 8 of ubiquinol cytochrome-c reductase (Complex III); Complex III is a component of the mitochondrial inner membrane electron transport chain; oriented facing the intermembrane space; expression is regulated by Abf1p and Cpf1p |
| **MRPS35** | 93 | 2.037 | -1.175 | Mitochondrial ribosomal protein of the small subunit; null mutant does not grow on glycerol, is sensitive to 2,4-dichlorophenol, and accumulates large lipid droplets |
| **COX10** | 94 | 2.036 | -0.865 | Heme A:farnesyltransferase; catalyzes first step in conversion of protoheme to heme A prosthetic group required for cytochrome c oxidase activity; human ortholog COX10 can complement yeast cox10 null mutant; human ortholog COX10 is associated with mitochondrial disorders |
| **SEM1** | 95 | 2.026 | -1.384 | 19S proteasome regulatory particle lid subcomplex component; role in Ub-dependent proteolysis and proteasome stability; involved in TREX-2 mediated mRNA export, and in the prevention of transcription-associated genome instability; ubiquitinated by Nedd4-like E3-ligase, Rsp5p; human ortholog DSS1, a BRCA1 binding protein implicated in cancer, complements the yeast null; drives trinucleotide repeat expansion; protein abundance increases in response to DNA replication stress |
| **TUF1** | 96 | 2.025 | -1.595 | Mitochondrial translation elongation factor Tu (EF-Tu); involved in fundamental pathway of mtDNA homeostasis; comprises both GTPase and guanine nucleotide exchange factor activities, while these activities are found in separate proteins in S. pombe and humans; rare mutations in human mitochondrial elongation factor Tu (EFTu) associated with severe lactic acidosis, rapidly progressive fatal encephalopathy, severe infantile macrocystic leukodystrophy with micropolygyria |
| **MSS51** | 97 | 2.017 | -0.727 | Specific translational activator for the mitochondrial COX1 mRNA; loosely associated with the matrix face of the mitochondrial inner membrane; localizes to vacuole membrane in response to H2O2; influences both COX1 mRNA translation and Cox1p assembly into cytochrome c oxidase; binds to heme B, which may be a mechanism for sensing oxygen levels in order to regulate cytochrome c oxidase biogenesis |
| **COX12** | 98 | 2.015 | -0.505 | Subunit VIb of cytochrome c oxidase; cytochrome c oxidase is also known as respiratory Complex IV and is the terminal member of the mitochondrial inner membrane electron transport chain; required for assembly of cytochrome c oxidase but not required for activity after assembly; phosphorylated; easily released from the intermembrane space, suggesting a loose association with Complex IV |
| **IMG2** | 99 | 2.009 | -1.157 | Mitochondrial ribosomal protein of the large subunit; conserved in metazoa, with similarity to human mitochondrial ribosomal protein MRPL49 |
| **MRPL16** | 100 | 2.004 | -1.273 | Mitochondrial ribosomal protein of the large subunit; homologous to bacterial L16 ribosomal protein; synthetic lethality with hac1 mutation suggests a possible role in synthesis of precursors for protein glycosylation |
| **MRPL20** | 102 | 1.993 | -1.31 | Mitochondrial ribosomal protein of the large subunit |
| **BCS1** | 101 | 1.993 | -0.592 | Protein translocase and chaperone required for Complex III assembly; member of the AAA ATPase family; forms a homo-oligomeric complex in the mitochondrial inner membrane that translocates the C-terminal domain of Rip1p from the matrix across the inner membrane and delivers it to an assembly intermediate of respiratory Complex III; also required for assembly of the Qcr10p subunit; mutation is functionally complemented by human homolog BCS1L, linked to neonatal diseases |
| **POR1** | 103 | 1.99 | -1.249 | Mitochondrial porin (voltage-dependent anion channel); outer membrane protein required for maintenance of mitochondrial osmotic stability and mitochondrial membrane permeability; couples the glutathione pools of the intermembrane space (IMS) and the cytosol; interacts with Om45 and Om14 in the outer membrane; phosphorylated; protein abundance increases in response to DNA replication stress |
| **MRP49** | 104 | 1.989 | -0.538 | Mitochondrial ribosomal protein of the large subunit; not essential for mitochondrial translation |
| **YGR102C** | 105 | 1.985 | -1.54 | Subunit of the trimeric GatFAB AmidoTransferase(AdT) complex; involved in the formation of Q-tRNAQ; transposon insertion mutant is salt sensitive and null mutant has growth defects; non-tagged protein is detected in purified mitochondria |
| **HAP3** | 106 | 1.967 | -0.26 | Subunit of the Hap2p/3p/4p/5p CCAAT-binding complex; complex is heme-activated and glucose-repressed; complex is a transcriptional activator and global regulator of respiratory gene expression; contains sequences contributing to both complex assembly and DNA binding |
| **MRPL10** | 107 | 1.955 | -1.466 | Mitochondrial ribosomal protein of the large subunit; appears as two protein spots (YmL10 and YmL18) on two-dimensional SDS gels |
| **MRPL37** | 108 | 1.939 | -1.716 | Mitochondrial ribosomal protein of the large subunit |
| **QCR9** | 109 | 1.92 | -0.362 | Subunit 9 of ubiquinol cytochrome-c reductase (Complex III); Complex III is a component of the mitochondrial inner membrane electron transport chain; required for electron transfer at the ubiquinol oxidase site of the complex |
| **MHR1** | 110 | 1.911 | -1.293 | Mitochondrial ribosomal protein of the large subunit; also involved in homologous recombination in mitochondria; required for recombination-dependent mtDNA partitioning; involved in stimulation of mitochondrial DNA replication in response to oxidative stress |
| **MRPL1** | 111 | 1.909 | -0.802 | Mitochondrial ribosomal protein of the large subunit |
| **PET309** | 112 | 1.906 | -1.467 | Specific translational activator for the COX1 mRNA; binds to the COX1 mRNA; also influences stability of intron-containing COX1 primary transcripts; localizes to the mitochondrial inner membrane; contains 12 pentatricopeptide repeats (PPRs) |
| **SLM5** | 113 | 1.898 | -1.4 | Mitochondrial asparaginyl-tRNA synthetase |
| **MEF1** | 114 | 1.892 | -1.014 | Mitochondrial elongation factor involved in translational elongation |
| **MRPL49** | 115 | 1.877 | -1.347 | Mitochondrial ribosomal protein of the large subunit |
| **SWS2** | 116 | 1.866 | -1.373 | Putative mitochondrial ribosomal protein of the small subunit; has similarity to E. coli S13 ribosomal protein; participates in controlling sporulation efficiency; localizes to vacuole in response to H2O2 |
| **QRI7** | 117 | 1.859 | -0.578 | Protein involved in threonylcarbamoyl adenosine biosynthesis; Sua5p and Qri7p are necessary and sufficient for RNA t6A modification in vitro; highly conserved mitochondrial protein; essential for t6A modification of mitochondrial tRNAs that decode ANN codons; similar to Kae1p and E. coli YgjD, both of which are also required for tRNA t6A modification; when directed to the cytoplasm, complements the essential function of Kae1p in the KEOPS complex |
| **ATP22** | 118 | 1.849 | -0.917 | Specific translational activator for the mitochondrial ATP6 mRNA; Atp6p encodes a subunit of F1F0 ATP synthase; localized to the mitochondrial inner membrane |
| **RSM22** | 119 | 1.844 | -1.352 | Mitochondrial ribosomal protein of the small subunit; also predicted to be an S-adenosylmethionine-dependent RNA methyltransferase |
| **ATP18** | 120 | 1.844 | -1.355 | Subunit of the mitochondrial F1F0 ATP synthase; F1F0 ATP synthase is a large, evolutionarily conserved enzyme complex required for ATP synthesis; termed subunit I or subunit j; does not correspond to known ATP synthase subunits in other organisms |
| **MRP21** | 121 | 1.833 | -1.59 | Mitochondrial ribosomal protein of the small subunit; MRP21 exhibits genetic interactions with mutations in the COX2 and COX3 mRNA 5'-untranslated leader sequences |
| **ATP23** | 122 | 1.817 | -0.698 | Putative metalloprotease of the mitochondrial inner membrane; required for processing of Atp6p; has an additional role in assembly of the F0 sector of the F1F0 ATP synthase complex; substrate of the Mia40p-Erv1p disulfide relay system, and folding is assisted by Mia40p |
| **MSS116** | 123 | 1.788 | -1.112 | Mitochondrial transcription elongation factor; DEAD-box protein; required for efficient splicing of mitochondrial Group I and II introns; non-polar RNA helicase that also facilities strand annealing; promotes RNA folding by stabilizing an early assembly intermediate |
| **MRPL22** | 124 | 1.771 | -0.935 | Mitochondrial ribosomal protein of the large subunit |
| **MRP7** | 125 | 1.769 | -1.312 | Mitochondrial ribosomal protein of the large subunit |
| **IMP1** | 126 | 1.758 | -0.702 | Catalytic subunit of mitochondrial inner membrane peptidase complex; required for maturation of mitochondrial proteins of the intermembrane space; complex contains two catalytic subunits (Imp1p and Imp2p that differ in substrate specificty) and Som1p |
| **UBX4** | 127 | 1.755 | -0.772 | UBX domain-containing protein that interacts with Cdc48p; involved in degradation of polyubiquitinated proteins via the ERAD (ER-associated degradation) pathway; modulates the Cdc48p-Nplp-Ufd1p AAA ATPase complex during its role in delivery of misfolded proteins to the proteasome; protein abundance increases in response to DNA replication stress |
| **MRPL35** | 128 | 1.737 | -1.528 | Mitochondrial ribosomal protein of the large subunit |
| **RIM101** | 129 | 1.734 | -0.854 | Cys2His2 zinc-finger transcriptional repressor; involved in alkaline responsive gene repression as part of adaptation to alkaline conditions; involved in cell wall assembly; required for alkaline pH-stimulated haploid invasive growth and sporulation; activated by alkaline-dependent proteolytic processing which results in removal of the C-terminal tail; similar to A. nidulans PacC |
| **MRPL4** | 130 | 1.728 | -1.675 | Mitochondrial ribosomal protein of the large subunit; homolog of prokaryotic L29 ribosomal protein; located at the ribosomal tunnel exit |
| **COX20** | 132 | 1.703 | -0.164 | Mitochondrial inner membrane protein; required for proteolytic processing of Cox2p and its assembly into cytochrome c oxidase |
| **COX11** | 133 | 1.68 | -0.744 | Protein required for delivery of copper to Cox1p; mitochondrial inner membrane protein; association with mitochondrial ribosomes suggests that copper delivery may occur during translation of Cox1p |
| **PPA2** | 134 | 1.663 | -1.252 | Mitochondrial inorganic pyrophosphatase; required for mitochondrial function and possibly involved in energy generation from inorganic pyrophosphate; human ortholog, PPA2, functionally complements the null mutant; mutations in human PPA2 cause a mitochondrial disease resulting in sudden unexpected cardiac arrest in infants |
| **MRPL33** | 135 | 1.64 | -1.337 | Mitochondrial ribosomal protein of the large subunit |
| **BUG1** | 136 | 1.638 | -0.163 | Cis-golgi localized protein involved in ER to Golgi transport; forms a complex with the mammalian GRASP65 homolog, Grh1p; mutants are compromised for the fusion of ER-derived vesicles with Golgi membranes |
| **NRP1** | 137 | 1.631 | -0.873 | Putative RNA binding hypothetical protein; localizes to stress granules induced by glucose deprivation; predicted to be involved in ribosome biogenesis |
| **RPO41** | 138 | 1.629 | -1.276 | Mitochondrial RNA polymerase; single subunit enzyme similar to those of T3 and T7 bacteriophages; requires a specificity subunit encoded by MTF1 for promoter recognition; Mtf1p interacts with and stabilizes the Rpo41p-promoter complex, enhancing DNA bending and melting to facilitate pre-initiation open complex formation; Rpo41p also synthesizes RNA primers for mitochondrial DNA replication |
| **YPL183W** | 140 | 1.624 | -0.494 | No Description Found |
| **MRPL15** | 139 | 1.624 | -1.643 | Mitochondrial ribosomal protein of the large subunit |
| **AIM31** | 141 | 1.607 | -0.271 | No Description Found |
| **YMR244C-A** | 142 | 1.604 | 0.675 | Protein involved in cytochrome c oxidase (Complex IV) assembly; involved in delivery of copper to Complex IV; also required for efficient formation of respiratory supercomplexes comprised of Complexes III and IV; localizes to the mitochondrial intermembrane space; ortholog implicated in cardiac defects in zebrafish and human; transcription is induced in response to the DNA-damaging agent MMS; protein abundance increases in response to DNA replication stress |
| **MRP10** | 143 | 1.555 | -1.029 | Mitochondrial ribosomal protein of the small subunit; contains twin cysteine-x9-cysteine motifs; oxidized by Mia40p during import into mitochondria |
| **OMS1** | 144 | 1.537 | -0.217 | Protein integral to the mitochondrial membrane; has a conserved methyltransferase motif and is predicted to be an RNA methyltransferase; multicopy suppressor of respiratory defects caused by OXA1 mutations |
| **ATP2** | 145 | 1.525 | -0.564 | Beta subunit of the F1 sector of mitochondrial F1F0 ATP synthase; which is a large, evolutionarily conserved enzyme complex required for ATP synthesis; F1 translationally regulates ATP6 and ATP8 expression to achieve a balanced output of ATP synthase genes encoded in nucleus and mitochondria; phosphorylated |
| **MRPS12** | 146 | 1.521 | -1.167 | Mitochondrial protein; may interact with ribosomes based on co-purification experiments; similar to E. coli and human mitochondrial S12 ribosomal proteins |
| **GDT1** | 147 | 1.506 | 0.085 | Calcium transporter localized to the cis- and medial-Golgi apparatus; required for protein glycosylation; GFP-fusion protein localizes to the vacuole; TMEM165, a human gene which causes Congenital Disorders of Glycosylation is orthologous and functionally complements the null allele; expression pattern and physical interactions suggest a possible role in ribosome biogenesis; expression reduced in a gcr1 null mutant |
| **RML2** | 148 | 1.482 | -1.504 | Mitochondrial ribosomal protein of the large subunit (L2); has similarity to E. coli L2 ribosomal protein; mutant allele (fat21) causes inability to utilize oleate, and induce oleic acid oxidation; may interfere with activity of the Adr1p transcription factor |
| **MAK10** | 149 | 1.481 | -0.222 | Non-catalytic subunit of the NatC N-terminal acetyltransferase; required for replication of dsRNA virus; expression is glucose-repressible; human NatC ortholog, Naa35, requires co-expression of the human catalytic subunit, Naa30, to functionally complement the null allele |
| **UFO1** | 150 | 1.478 | -0.485 | F-box receptor protein; subunit of the Skp1-Cdc53-F-box receptor (SCF) E3 ubiquitin ligase complex; binds to phosphorylated Ho endonuclease, allowing its ubiquitination by SCF and subsequent degradation |
| **OST4** | 151 | 1.476 | -1.58 | Subunit of the oligosaccharyltransferase complex of the ER lumen; complex catalyzes protein asparagine-linked glycosylation; type I membrane protein required for incorporation of Ost3p or Ost6p into the OST complex |
| **MRPL40** | 152 | 1.472 | -1.533 | Mitochondrial ribosomal protein of the large subunit |
| **RSM19** | 153 | 1.418 | -1.292 | Mitochondrial ribosomal protein of the small subunit; has similarity to E. coli S19 ribosomal protein |
| **MAC1** | 154 | 1.417 | -0.609 | Copper-sensing transcription factor; involved in regulation of genes required for high affinity copper transport; required for regulation of yeast copper genes in response to DNA-damaging agents; undergoes changes in redox state in response to changing levels of copper or MMS |
| **COX16** | 155 | 1.399 | -1.817 | Mitochondrial inner membrane protein; required for assembly of cytochrome c oxidase |
| **PET127** | 156 | 1.394 | -0.32 | Protein with a role in 5'-end processing of mitochondrial RNAs; located in the mitochondrial membrane |
| **VAM3** | 157 | 1.389 | 0.004 | Syntaxin-like vacuolar t-SNARE; functions with Vam7p in vacuolar protein trafficking; mediates docking/fusion of late transport intermediates with the vacuole; has an acidic di-leucine sorting signal and C-terminal transmembrane region |
| **YJR120W** | 158 | 1.384 | -0.499 | hypothetical protein; essential for growth under anaerobic conditions; mutation causes decreased expression of ATP2, impaired respiration, defective sterol uptake, and altered levels/localization of ABC transporters Aus1p and Pdr11p |
| **RPN4** | 159 | 1.379 | -0.327 | Transcription factor that stimulates expression of proteasome genes; Rpn4p levels are in turn regulated by the 26S proteasome in a negative feedback control mechanism; RPN4 is transcriptionally regulated by various stress responses; relative distribution to the nucleus increases upon DNA replication stress |
| **ATP12** | 160 | 1.378 | -0.901 | Assembly factor for F1 sector of mitochondrial F1F0 ATP synthase; conserved protein; required for assembly of alpha and beta subunits into F1 sector of mitochondrial F1F0 ATP synthase; human homolog ATPAF2 can complement yeast atp12 mutant; mutation of human homolog reduces active ATP synthase levels and is associated with the disorder ATPAF2 deficiency |
| **MAK3** | 161 | 1.367 | 0.395 | Catalytic subunit of the NatC type N-terminal acetyltransferase (NAT); involved in subcellular targeting of select N-terminally acetylated substrates to the Golgi apparatus (Arl3p and Grh1p) and the inner nuclear membrane (Trm1p); required for replication of dsRNA virus; human NatC ortholog, Naa60, functionally complements the null, requiring either auxiliary subunit Mak10p or co-expression of human ortholog, Naa35; Naa60, the human NatF gene, also complements the null allele |
| **SSN3** | 162 | 1.361 | -0.651 | Cyclin-dependent protein kinase; component of RNA polymerase II holoenzyme; involved in phosphorylation of the RNA polymerase II C-terminal domain; involved in glucose repression |
| **CYC2** | 163 | 1.337 | -0.348 | Mitochondrial peripheral inner membrane protein; contains a FAD cofactor in a domain exposed in the intermembrane space; exhibits redox activity in vitro; likely participates in ligation of heme to acytochromes c and c1 (Cyc1p and Cyt1p) |
| **COX8** | 164 | 1.336 | 0.048 | Subunit VIII of cytochrome c oxidase (Complex IV); Complex IV is the terminal member of the mitochondrial inner membrane electron transport chain |
| **CRD1** | 165 | 1.328 | 0.085 | Cardiolipin synthase; produces cardiolipin, which is a phospholipid of the mitochondrial inner membrane that is required for normal mitochondrial membrane potential and function and for correct integration of membrane-multispanning proteins into the mitochondrial outer membrane; required to maintain tubular mitochondrial morphology and functions in mitochondrial fusion; also required for normal vacuolar ion homeostasis |
| **PRY1** | 166 | 1.32 | 0.343 | Sterol binding protein involved in the export of acetylated sterols; secreted glycoprotein and member of the CAP protein superfamily (cysteine-rich secretory proteins (CRISP), antigen 5, and pathogenesis related 1 proteins); sterol export function is redundant with that of PRY2; may be involved in detoxification of hydrophobic compounds; PRY1 has a paralog, PRY2, that arose from the whole genome duplication |
| **ATP7** | 167 | 1.293 | -1.563 | Subunit d of the stator stalk of mitochondrial F1F0 ATP synthase; F1F0 ATP synthase is a large, evolutionarily conserved enzyme complex required for ATP synthesis |
| **YER084W** | 168 | 1.287 | -0.02 | hypothetical protein; expressed at both mRNA and protein levels |
| **MRPS8** | 169 | 1.283 | -1.335 | Mitochondrial ribosomal protein of the small subunit |
| **NIP100** | 170 | 1.273 | -1.064 | Large subunit of the dynactin complex; dynactin is involved in partitioning the mitotic spindle between mother and daughter cells; putative ortholog of mammalian p150(glued) |
| **COG1** | 171 | 1.27 | -1.577 | Essential component of the conserved oligomeric Golgi complex; a cytosolic tethering complex (Cog1p through Cog8p) that functions in protein trafficking to mediate fusion of transport vesicles to Golgi compartments |
| **YGL079W** | 172 | 1.269 | -0.154 | Subunit of the BLOC-1 complex involved in endosomal maturation; null mutant is sensitive to drug inducing secretion of vacuolar cargo; GFP-fusion protein localizes to the endosome |
| **ICP55** | 173 | 1.267 | 0.084 | Mitochondrial aminopeptidase; cleaves the N termini of at least 38 imported proteins after cleavage by the mitochondrial processing peptidase (MPP), thereby increasing their stability; member of the aminopeptidase P family |
| **YDR493W** | 174 | 1.25 | -0.135 | Protein required for assembly of the cytochrome bc(1) complex; acts as a chaperone for Rip1p and facilitates its insertion into the complex at a late stage of assembly; localized to the mitochondrial matrix; null mutant exhibits a respiratory growth defect and reduced mitochondrial zinc levels, which is characteristic of mutations affecting bc(1) complex assembly; member of the LYR protein family; human LYRM7 is a functional ortholog |
| **MMR1** | 175 | 1.238 | 0.106 | Phosphorylated protein of the mitochondrial outer membrane; localizes only to mitochondria of the bud; interacts with Myo2p to mediate mitochondrial distribution to buds; mRNA is targeted to the bud via the transport system involving She2p |
| **CYC1** | 176 | 1.236 | -0.149 | Cytochrome c, isoform 1; also known as iso-1-cytochrome c; electron carrier of mitochondrial intermembrane space that transfers electrons from ubiquinone-cytochrome c oxidoreductase to cytochrome c oxidase during cellular respiration; CYC1 has a paralog, CYC7, that arose from the whole genome duplication; human homolog CYC1 can complement yeast null mutant; mutations in human CYC1 cause insulin-responsive hyperglycemia |
| **GEF1** | 177 | 1.235 | -0.261 | Voltage-gated chloride channel; localized to the golgi, the endosomal system, and plasma membrane; involved in cation homeostasis; highly homologous to vertebrate voltage-gated chloride channels; modulates TBSV model (+) RNA virus replication by regulating copper metabolism |
| **STV1** | 178 | 1.226 | 0.895 | Subunit a of the vacuolar-ATPase V0 domain; one of two isoforms (Stv1p and Vph1p); Stv1p is located in V-ATPase complexes of the Golgi and endosomes while Vph1p is located in V-ATPase complexes of the vacuole |
| **YSC83** | 179 | 1.213 | 0.581 | Non-essential mitochondrial hypothetical protein; mRNA induced during meiosis, peaking between mid to late prophase of meiosis I; similar to S. douglasii YSD83 |
| **MRPL25** | 180 | 1.204 | -1.518 | Mitochondrial ribosomal protein of the large subunit; mutation confers increased replicative lifespan |
| **YML009c** | 181 | 1.202 | 0.069 | Mitochondrial ribosomal protein of the large subunit |

**SUPPLEMENTARY TABLE S6: Bottom Betaxanthin Hits**

| **gene** | **Yield_Rank** | **Yield_Score** | **Size_Score** | **Description** |
| --- | --- | --- | --- | --- |
| **BUD25** | 4227 | -2.876 | -2.56 | Protein involved in bud-site selection; diploid mutants display a random budding pattern instead of the wild-type bipolar pattern |
| **THP1** | 4226 | -2.696 | -2.586 | Nuclear pore-associated protein; component of TREX-2 complex (Sac3p-Thp1p-Sus1p-Cdc31p) involved in transcription elongation and mRNA export from the nucleus; involved in post-transcriptional tethering of active genes to the nuclear periphery and to non-nascent mRNP; contains a PAM domain implicated in protein-protein binding |
| **PRS3** | 4225 | -2.628 | -2.227 | 5-phospho-ribosyl-1(alpha)-pyrophosphate synthetase; synthesizes PRPP, which is required for nucleotide, histidine, and tryptophan biosynthesis; one of five related enzymes, which are active as heteromultimeric complexes |
| **GAL10** | 4224 | -2.588 | -1.592 | UDP-glucose-4-epimerase; catalyzes interconversion of UDP-galactose and UDP-D-glucose in galactose metabolism; also catalyzes conversion of alpha-D-glucose or alpha-D-galactose to their beta-anomers; human homolog GALE implicated in galactosemia, can complement yeast null mutant |
| **VMA13** | 4223 | -2.561 | -1.948 | Subunit H of the V1 peripheral membrane domain of V-ATPase; part of the electrogenic proton pump found throughout the endomembrane system; serves as an activator or a structural stabilizer of the V-ATPase; the V1 peripheral membrane domain of the vacuolar H+-ATPase (V-ATPase) has eight subunits |
| **APQ12** | 4222 | -2.405 | -2.158 | Nuclear envelope/ER integral membrane protein; interacts and functions with Brr6p and Brl1p in lipid homeostasis; mutants are defective in nuclear pore complex biogenesis, nuclear envelope morphology, mRNA export from the nucleus and are sensitive to sterol biosynthesis inhibitors and membrane fluidizing agents; exhibits synthetic lethal genetic interactions with genes involved in lipid metabolism |
| **BUR2** | 4221 | -2.368 | -1.14 | Cyclin for the Sgv1p (Bur1p) protein kinase; Sgv1p and Bur2p comprise the CDK-cyclin BUR kinase complex which is involved in transcriptional regulation through its phosphorylation of the carboxy-terminal domain (CTD) of the largest subunit of RNA polymerase II (Rpo21p); BUR kinase is also involved in the recruitment of Spt6p to the CTD at the onset of transcription |
| **CAX4** | 4220 | -2.348 | -2.555 | Dolichyl pyrophosphate (Dol-P-P) phosphatase; has a luminally oriented active site in the ER; cleaves the anhydride linkage in Dol-P-P; required for Dol-P-P-linked oligosaccharide intermediate synthesis and protein N-glycosylation |
| **ZUO1** | 4219 | -2.32 | -2.31 | Ribosome-associated chaperone; zuotin functions in ribosome biogenesis and as a chaperone for nascent polypeptide chains in partnership with Ssz1p and SSb1/2; contains a DnaJ domain and functions as a J-protein partner for Ssb1p and Ssb2p; human gene DNAJC2 can partially complement yeast zuo1 null mutant |
| **VPS34** | 4218 | -2.286 | -1.326 | Phosphatidylinositol (PI) 3-kinase that synthesizes PI-3-phosphate; forms membrane-associated signal transduction complex with Vps15p to regulate protein sorting; activated by the GTP-bound form of Gpa1p; a fraction is localized, with Vps15p, to nuclear pores at nucleus-vacuole junctions and may facilitate transcription elongation for genes positioned at the nuclear periphery |
| **DBP7** | 4217 | -2.286 | -2.711 | Putative ATP-dependent RNA helicase of the DEAD-box family; involved in ribosomal biogenesis; required at post-transcriptional step for efficient retrotransposition; essential for growth under anaerobic conditions |
| **MAP1** | 4216 | -2.276 | -1.929 | Methionine aminopeptidase; catalyzes the cotranslational removal of N-terminal methionine from nascent polypeptides; function is partially redundant with that of Map2p |
| **PDX3** | 4215 | -2.218 | -0.592 | Pyridoxine (pyridoxamine) phosphate oxidase; has homologs in E. coli and Myxococcus xanthus; transcription is under the general control of nitrogen metabolism |
| **RPS10A** | 4214 | -2.211 | -2.328 | Protein component of the small (40S) ribosomal subunit; homologous to mammalian ribosomal protein S10, no bacterial homolog; RPS10A has a paralog, RPS10B, that arose from the whole genome duplication; mutations in the human homolog associated with Diamond-Blackfan anemia |
| **ERG3** | 4213 | -2.204 | -2.125 | C-5 sterol desaturase; glycoprotein that catalyzes the introduction of a C-5(6) double bond into episterol, a precursor in ergosterol biosynthesis; transcriptionally down-regulated when ergosterol is in excess; mutants are viable, but cannot grow on non-fermentable carbon sources; substrate of HRD ubiquitin ligase; mutation is functionally complemented by human SC5D |
| **RTF1** | 4212 | -2.172 | -1.319 | Subunit of RNAPII-associated chromatin remodeling Paf1 complex; regulates gene expression by directing cotranscriptional histone modification, influences transcription and chromatin structure through several independent functional domains; directly or indirectly regulates DNA-binding properties of Spt15p and relative activities of different TATA elements; involved in transcription elongation as demonstrated by the G-less-based run-on (GLRO) assay |
| **RPL27A** | 4211 | -2.118 | -2.196 | Ribosomal 60S subunit protein L27A; homologous to mammalian ribosomal protein L27, no bacterial homolog; RPL27A has a paralog, RPL27B, that arose from the whole genome duplication |
| **NUP84** | 4210 | -2.097 | -2.371 | Subunit of the Nup84p subcomplex of the nuclear pore complex (NPC); contributes to nucleocytoplasmic transport and NPC biogenesis; also plays roles in several processes that may require localization of genes or chromosomes at the nuclear periphery, including double-strand break repair, transcription and chromatin silencing; homologous to human NUP107 |
| **YGL081W** | 4209 | -2.095 | -1.939 | hypothetical protein; non-essential gene; interacts genetically with CHS5, a gene involved in chitin biosynthesis |
| **PRO2** | 4208 | -2.085 | -2.059 | Gamma-glutamyl phosphate reductase; catalyzes the second step in proline biosynthesis |
| **1.00 BRE** | 4207 | -2.064 | -1.146 | E3 ubiquitin ligase; forms heterodimer with Rad6p to regulate K63 polyubiquitination in response to oxidative stress and to monoubiquinate histone H2B-K123, which is required for the subsequent methylation of histone H3-K4 and H3-K79; required for DSBR, transcription, silencing, and checkpoint control; interacts with RNA-binding protein Npl3p, linking histone ubiquitination to mRNA processing; Bre1p-dependent histone ubiquitination promotes pre-mRNA splicing |
| **YDL119C** | 4206 | -2.063 | -0.958 | Mitochondrial glycine transporter; required for the transport of glycine into mitochondria for initiation of heme biosynthesis, with YMC1 acting as a secondary transporter; homolog of human SLC25A38, a mitochondrial glycine transporter associated with nonsyndromic autosomal recessive congenital sideroblastic anemia; human SLC25A38 can complement the heme deficiency associated with the null mutant; GFP-fusion protein is induced in response to the DNA-damaging agent MMS |
| **SLM2** | 852 | -2.058 | -2.379 | Phosphoinositide PI4,5P(2) binding protein, forms a complex with Slm1p; acts downstream of Mss4p in a pathway regulating actin cytoskeleton organization in response to stress; TORC2 complex substrate and effector; SLM2 has a paralog, SLM1, that arose from the whole genome duplication |
| **VMA2** | 4204 | -2.054 | -1.902 | Subunit B of V1 peripheral membrane domain of vacuolar H+-ATPase; electrogenic proton pump found throughout the endomembrane system; contains nucleotide binding sites; also detected in the cytoplasm; protein abundance increases in response to DNA replication stress; human homolog ATP6V1B1, implicated in autosomal-recessive distal renal tubular acidosis (RTA) with sensorineural deafness, complements yeast null mutant |
| **ANP1** | 4203 | -2.051 | -1.709 | Subunit of the alpha-1,6 mannosyltransferase complex; type II membrane protein; has a role in retention of glycosyltransferases in the Golgi; involved in osmotic sensitivity and resistance to aminonitrophenyl propanediol |
| **RRN10** | 4202 | -2.045 | -1.957 | Protein involved in promoting high level transcription of rDNA; subunit of UAF (upstream activation factor) for RNA polymerase I |
| **SLM4** | 4201 | -2.044 | -2.082 | Component of the EGO and GSE complexes; essential for integrity and function of EGO; EGO is involved in the regulation of microautophagy and GSE is required for proper sorting of amino acid permease Gap1p; gene exhibits synthetic genetic interaction with MSS4 |
| **SFP1** | 4200 | -2.031 | -1.974 | Regulates transcription of ribosomal protein and biogenesis genes; regulates response to nutrients and stress, G2/M transitions during mitotic cell cycle and DNA-damage response, and modulates cell size; regulated by TORC1 and Mrs6p; sequence of zinc finger, ChIP localization data, and protein-binding microarray (PBM) data, and computational analyses suggest it binds DNA directly at highly active RP genes and indirectly through Rap1p at others; can form the [ISP+] prion |
| **RPS19B** | 4199 | -2.023 | -2.396 | Protein component of the small (40S) ribosomal subunit; required for assembly and maturation of pre-40 S particles; homologous to mammalian ribosomal protein S19, no bacterial homolog; mutations in human RPS19 are associated with Diamond Blackfan anemia; RPS19B has a paralog, RPS19A, that arose from the whole genome duplication |
| **PHO88** | 4198 | -2.021 | -1.957 | Protein involved in SRP-independent targeting of substrates to the ER; component of an alternative ER targeting pathway that has partial functional redundancy with the GET pathway; preference for substrates with downstream transmembrane domains; interacts with Snd1p, Env10p/Snd2p, and Sec61p-translocon subunits; can compensate for loss of SRP; role in phosphate transport, interacting with pho88, and in the maturation of secretory proteins |
| **KGD2** | 4196 | -2.013 | -0.276 | Dihydrolipoyl transsuccinylase; component of the mitochondrial alpha-ketoglutarate dehydrogenase complex, which catalyzes the oxidative decarboxylation of alpha-ketoglutarate to succinyl-CoA in the TCA cycle; phosphorylated |
| **UAF30** | 4197 | -2.013 | -2.153 | Subunit of UAF (upstream activation factor) complex; UAF is an RNA polymerase I specific transcription stimulatory factor composed of Uaf30p, Rrn5p, Rrn9p, Rrn10p, histones H3 and H4; targeting factor for the UAF that facilitates activation of many rDNA genes; deletion decreases cellular growth rate; UAF30 has a paralog, TRI1, that arose from the whole genome duplication |
| **GON7** | 4195 | -2.006 | -2.108 | Component of the EKC/KEOPS protein complex; EKC/KEOPS complex is required for t6A tRNA modification and telomeric TG1-3 recombination; may have role in transcription; implicated in osmotic stress response; other complex members are Kae1p, Cgi121p, Pcc1p, and Bud32p |
| **YHR039C-B** | 4194 | -1.994 | -2.111 | No Description Found |
| **VMA22** | 4193 | -1.99 | -1.698 | Protein that is required for vacuolar H+-ATPase (V-ATPase) function; peripheral membrane protein; not an actual component of the V-ATPase complex; functions in the assembly of the V-ATPase; localized to the yeast endoplasmic reticulum (ER) |
| **PEP12** | 4192 | -1.982 | -2.276 | Target membrane receptor (t-SNARE); for vesicular intermediates traveling between the Golgi apparatus and the vacuole; controls entry of biosynthetic, endocytic, and retrograde traffic into the prevacuolar compartment; syntaxin |
| **BUD31** | 4191 | -1.974 | -2.479 | Component of the SF3b subcomplex of the U2 snRNP; increases efficiency of first and second step pre-mRNA splicing; diploid mutants display a random budding pattern instead of the wild-type bipolar pattern; facilitates passage through G1/S Start, but is not required for G2/M transition or exit from mitosis |
| **SNF7** | 4190 | -1.973 | -0.215 | One of four subunits of the ESCRT-III complex; involved in the sorting of transmembrane proteins into the multivesicular body (MVB) pathway; recruited from the cytoplasm to endosomal membranes; ESCRT-III stands for endosomal sorting complex required for transport III |
| **BDF1** | 4189 | -1.964 | -2.019 | Protein involved in transcription initiation; functions at TATA-containing promoters; associates with the basal transcription factor TFIID; contains two bromodomains; corresponds to the C-terminal region of mammalian TAF1; redundant with Bdf2p; BDF1 has a paralog, BDF2, that arose from the whole genome duplication |
| **PEX32** | 4188 | -1.963 | -1.666 | Peroxisomal integral membrane protein; involved in negative regulation of peroxisome size; partially functionally redundant with Pex31p; genetic interactions suggest action at a step downstream of steps mediated by Pex28p and Pex29p |
| **RPS17A** | 4187 | -1.952 | -2.668 | Ribosomal protein 51 (rp51) of the small (40s) subunit; homologous to mammalian ribosomal protein S17, no bacterial homolog; RPS17A has a paralog, RPS17B, that arose from the whole genome duplication |
| **GAL4** | 4186 | -1.949 | -1.9 | DNA-binding transcription factor required for activating GAL genes; responds to galactose; repressed by Gal80p and activated by Gal3p |
| **INO4** | 4185 | -1.947 | -2.31 | Transcription factor involved in phospholipid synthesis; required for derepression of inositol-choline-regulated genes involved in phospholipid synthesis; forms a complex, with Ino2p, that binds the inositol-choline-responsive element through a basic helix-loop-helix domain |
| **VMA6** | 4184 | -1.938 | -2.338 | Subunit d of the V0 integral membrane domain of V-ATPase; part of the electrogenic proton pump found in the endomembrane system; required for V1 domain assembly on the vacuolar membrane; the V0 integral membrane domain of vacuolar H+-ATPase (V-ATPase) has five subunits |
| **SNX4** | 4183 | -1.929 | -1.286 | Sorting nexin; involved in retrieval of late-Golgi SNAREs from post-Golgi endosomes to the trans-Golgi network and in cytoplasm to vacuole transport; contains a PX phosphoinositide-binding domain; forms complexes with Snx41p and with Atg20p |
| **THR1** | 4182 | -1.908 | -1.864 | Homoserine kinase; conserved protein required for threonine biosynthesis; long-lived protein that is preferentially retained in mother cells and forms cytoplasmic filaments; expression is regulated by the GCN4-mediated general amino acid control pathway |
| **ILV1** | 4181 | -1.902 | -2.294 | Threonine deaminase, catalyzes first step in isoleucine biosynthesis; expression is under general amino acid control; ILV1 locus exhibits highly positioned nucleosomes whose organization is independent of known ILV1 regulation |
| **PUT2** | 4180 | -1.888 | 0.043 | Delta-1-pyrroline-5-carboxylate dehydrogenase; nuclear-encoded mitochondrial protein involved in utilization of proline as sole nitrogen source; deficiency of human homolog ALDH4A1 causes type II hyperprolinemia (HPII), an autosomal recessive inborn error of metabolism; human homolog ALDH4A1 can complement yeast null mutant |
| **JJJ1** | 4179 | -1.88 | -1.67 | Co-chaperone that stimulates the ATPase activity of Ssa1p; required for a late step of ribosome biogenesis; associated with the cytosolic large ribosomal subunit; contains a J-domain; mutation causes defects in fluid-phase endocytosis |
| **RPL19B** | 4178 | -1.872 | -1.795 | Ribosomal 60S subunit protein L19B; rpl19a and rpl19b single null mutations result in slow growth, while the double null mutation is lethal; homologous to mammalian ribosomal protein L19, no bacterial homolog; RPL19B has a paralog, RPL19A, that arose from the whole genome duplication |
| **SKI7** | 4177 | -1.854 | -2.449 | GTP-binding protein that couples the Ski complex and exosome; putative pseudo-translational GTPase involved in 3'-to-5' mRNA decay pathway; interacts with both the cytoplasmic exosome and the Ski complex; eRF3-like domain targets nonstop mRNA for degradation; null mutants have a superkiller phenotype; SKI7 has a paralog, HBS1, that arose from the whole genome duplication |
| **SNT309** | 4176 | -1.852 | -0.812 | Member of the NineTeen Complex (NTC); this complex contains Prp19p and stabilizes U6 snRNA in catalytic forms of the spliceosome containing U2, U5, and U6 snRNAs; interacts physically and genetically with Prp19p |
| **PPA1** | 4175 | -1.836 | -1.962 | No Description Found |
| **SAP30** | 4174 | -1.834 | -0.847 | Component of Rpd3L histone deacetylase complex; involved in silencing at telomeres, rDNA, and silent mating-type loci; involved in telomere maintenance |
| **VPS25** | 4173 | -1.829 | -0.513 | Component of the ESCRT-II complex; ESCRT-II is involved in ubiquitin-dependent sorting of proteins into the endosome |
| **VMA5** | 4172 | -1.828 | -1.91 | Subunit C of the V1 peripheral membrane domain of V-ATPase; part of the electrogenic proton pump found throughout the endomembrane system; required for the V1 domain to assemble onto the vacuolar membrane; the V1 peripheral membrane domain of vacuolar H+-ATPase (V-ATPase) has eight subunits |
| **ARO1** | 4171 | -1.825 | -0.798 | Pentafunctional arom protein; catalyzes steps 2 through 6 in the biosynthesis of chorismate, which is a precursor to aromatic amino acids |
| **1.00 SHP** | 4170 | -1.809 | -2.224 | UBX domain-containing substrate adaptor for Cdc48p; ubiquitin regulatory X domain-containing protein that acts as a substrate recruiting cofactor for Cdc48p; positively regulates Glc7p PPase activity to promote growth and mitotic progression in complex with Cdc48p; ubiquitinated protein interactor involved in ER-associated degradation (ERAD); regulated by nuclear Ub-dependent degradation (INMAD pathway) independent of the Asi and Doa10 complexes; homolog of human p47 (NSFL1C) |
| **GCN4** | 4169 | -1.799 | -1.841 | bZIP transcriptional activator of amino acid biosynthetic genes; activator responds to amino acid starvation; expression is tightly regulated at both the transcriptional and translational levels |
| **RPL14A** | 4168 | -1.792 | -1.92 | Ribosomal 60S subunit protein L14A; N-terminally acetylated; homologous to mammalian ribosomal protein L14, no bacterial homolog; RPL14A has a paralog, RPL14B, that arose from the whole genome duplication |
| **ADE12** | 4167 | -1.792 | -2.008 | Adenylosuccinate synthase; catalyzes the first step in synthesis of adenosine monophosphate from inosine 5'monophosphate during purine nucleotide biosynthesis; exhibits binding to single-stranded autonomously replicating (ARS) core sequence |
| **YDJ1** | 4166 | -1.787 | -2.33 | Type I HSP40 co-chaperone; involved in regulation of HSP90 and HSP70 functions; acts as an adaptor that helps Rsp5p recognize cytosolic misfolded proteins for ubiquitination after heat shock; critical for determining cell size at Start as a function of growth rate; involved in protein translocation across membranes; member of the DnaJ family; chimeric protein in which human p58IPK J domain replaces yeast Ydj1p J domain can complement yeast ydj1 mutant |
| **RPL13B** | 4164 | -1.785 | -2.465 | Ribosomal 60S subunit protein L13B; not essential for viability; homologous to mammalian ribosomal protein L13, no bacterial homolog; RPL13B has a paralog, RPL13A, that arose from the whole genome duplication |
| **NUP133** | 4165 | -1.785 | -1.333 | Subunit of Nup84p subcomplex of nuclear pore complex (NPC); contributes to nucleocytoplasmic transport, NPC biogenesis; is involved in establishment of a normal nucleocytoplasmic concentration gradient of GTPase Gsp1p; also plays roles in several processes that may require localization of genes or chromosomes at nuclear periphery, including double-strand break repair, transcription and chromatin silencing; relocalizes to cytosol in response to hypoxia; homolog of human NUP133 |
| **ZAP1** | 4163 | -1.778 | -2.274 | Zinc-regulated transcription factor; binds to zinc-responsive promoters to induce transcription of certain genes in presence of zinc, represses other genes in low zinc; regulates its own transcription; contains seven zinc-finger domains |
| **ERG2** | 4162 | -1.773 | -1.505 | C-8 sterol isomerase; catalyzes isomerization of delta-8 double bond to delta-7 position at an intermediate step in ergosterol biosynthesis; transcriptionally down-regulated when ergosterol is in excess; mutation is functionally complemented by human EBP |
| **VPS20** | 4161 | -1.77 | -0.544 | Myristoylated subunit of the ESCRT-III complex; the endosomal sorting complex required for transport of transmembrane proteins into the multivesicular body pathway to the lysosomal/vacuolar lumen; cytoplasmic protein recruited to endosomal membranes |
| **LST7** | 4160 | -1.755 | -0.978 | Subunit of the Lst4p-Lst7p GTPase activating protein complex for Gtr2p; stimulates the GTPase activity of Rag family GTPase Gtr2p, within the context of the Gtr1p-Gtr2p heterodimer, after amino acid stimulation; required for activation of TORC1 in response to amino acid stimulation; recruited to the vacuolar membrane during amino acid starvation and released from the membrane by TORC1; required for the transport of amino acid permease Gap1p from the Golgi to the cell surface |
| **RNR1** | 4159 | -1.753 | -1.804 | Major isoform of large subunit of ribonucleotide-diphosphate reductase; the RNR complex catalyzes rate-limiting step in dNTP synthesis, regulated by DNA replication and DNA damage checkpoint pathways via localization of small subunits; relative distribution to the nucleus increases upon DNA replication stress; RNR1 has a paralog, RNR3, that arose from the whole genome duplication |
| **VPS9** | 4158 | -1.738 | -1.351 | Guanine nucleotide exchange factor (GEF) and ubiquitin receptor; involved in vesicle-mediated vacuolar transport, including Golgi-endosome trafficking and sorting through the multivesicular body (MVB); stimulates the intrinsic guanine nucleotide exchange activity of Rab family members (Vps21p/Ypt52p/Ypt53p); partially redundant with GEF MUK1; required for localization of the CORVET complex to endosomes; similar to mammalian ras inhibitors; contains a Ub-interacting CUE domain |
| **ASF1** | 4157 | -1.733 | -1.688 | Nucleosome assembly factor; involved in chromatin assembly, disassembly; required for recovery after DSB repair; role in H3K56 acetylation required for expression homeostasis, buffering mRNA synthesis rate against gene dosage changes in S phase; anti-silencing protein, derepresses silent loci when overexpressed; role in regulating Ty1 transposition; relocalizes to cytosol under hypoxia; growth defect of asf1 null is functionally complemented by either human ASF1A or ASF1B |
| **MTD1** | 4156 | -1.73 | -0.674 | NAD-dependent 5,10-methylenetetrahydrafolate dehydrogenase; plays a catalytic role in oxidation of cytoplasmic one-carbon units; expression is regulated by Bas1p and Bas2p, repressed by adenine, and may be induced by inositol and choline |
| **REF2** | 4155 | -1.726 | -2.698 | RNA-binding protein; involved in the cleavage step of mRNA 3'-end formation prior to polyadenylation, and in snoRNA maturation; part of holo-CPF subcomplex APT, which associates with 3'-ends of snoRNA- and mRNA-encoding genes; putative regulatory subunit of type 1 protein phosphatase Glc7p, required for actomyosin ring formation, and for timely dephosphorylation and release of Bnr1p from the division site; relocalizes to the cytosol in response to hypoxia |
| **5.00 CUP** | 4154 | -1.723 | -1.837 | No Description Found |
| **RPS27B** | 4153 | -1.721 | -0.953 | Protein component of the small (40S) ribosomal subunit; homologous to mammalian ribosomal protein S27, no bacterial homolog; RPS27B has a paralog, RPS27A, that arose from the whole genome duplication |
| **SSE1** | 4152 | -1.715 | -2.065 | ATPase component of heat shock protein Hsp90 chaperone complex; serves as nucleotide exchange factor to load ATP onto the SSA class of cytosolic Hsp70s; plays a role in prion propagation and determining prion variants; binds unfolded proteins; member of Hsp110 subclass of HSP70 proteins; deletion results in spindle elongation in S phase; SSE1 has a paralog, SSE2, that arose from the whole genome duplication |
| **MRE11** | 4151 | -1.702 | -1.591 | Nuclease subunit of the MRX complex with Rad50p and Xrs2p; complex functions in repair of DNA double-strand breaks and in telomere stability; Mre11p associates with Ser/Thr-rich ORFs in premeiotic phase; nuclease activity required for MRX function; widely conserved; forms nuclear foci upon DNA replication stress |
| **VPS28** | 4150 | -1.694 | -0.582 | Component of the ESCRT-I complex; complex is involved in ubiquitin-dependent sorting of proteins into the endosome; conserved C-terminal domain interacts with ESCRT-III subunit Vps20p; other members include Stp22p, Srn2p, Vps28p, and Mvb12p |
| **MOT2** | 4149 | -1.687 | -1.793 | Ubiquitin-protein ligase subunit of the CCR4-NOT complex; with Ubc4p, ubiquitinates nascent polypeptide-associated complex subunits and histone demethyase Jhd2p; CCR4-NOT has roles in transcription regulation, mRNA degradation, and post-transcriptional modifications; regulates levels of DNA Polymerase-{alpha} to promote efficient and accurate DNA replication |
| **IBA57** | 4148 | -1.677 | -2.133 | Protein involved in incorporating iron-sulfur clusters into proteins; mitochondrial matrix protein; involved in the incorporation of iron-sulfur clusters into mitochondrial aconitase-type proteins; activates the radical-SAM family members Bio2p and Lip5p; interacts with Ccr4p in the two-hybrid system |
| **LTV1** | 4147 | -1.676 | -2.422 | Component of the GSE complex; GSE is required for proper sorting of amino acid permease Gap1p; required for ribosomal small subunit export from nucleus; required for growth at low temperature |
| **SPT4** | 4146 | -1.672 | -1.925 | Spt4p/5p (DSIF) transcription elongation factor complex subunit; the Spt4/5 complex binds to ssRNA in a sequence-specific manner, and along with RNAP I and II has multiple roles regulating transcriptional elongation, RNA processing, quality control, and transcription-coupled repair; localizes to kinetochores and heterochromatin, influencing chromosomal dynamics and silencing; required for transcription through long trinucleotide repeats in ORFs and non-protein coding regions |
| **KGD1** | 4145 | -1.67 | -1.184 | Subunit of the mitochondrial alpha-ketoglutarate dehydrogenase complex; catalyzes a key step in the tricarboxylic acid (TCA) cycle, the oxidative decarboxylation of alpha-ketoglutarate to form succinyl-CoA |
| **RPS16B** | 4144 | -1.666 | -1.526 | Protein component of the small (40S) ribosomal subunit; homologous to mammalian ribosomal protein S16 and bacterial S9; RPS16B has a paralog, RPS16A, that arose from the whole genome duplication |
| **YGL138C** | 4143 | -1.664 | -0.92 | hypothetical protein; has no significant sequence similarity to any known protein |
| **PLC1** | 4142 | -1.655 | -1.107 | Phospholipase C; hydrolyzes phosphatidylinositol 4,5-biphosphate (PIP2) to generate the signaling molecules inositol 1,4,5-triphosphate (IP3) and 1,2-diacylglycerol (DAG); involved in regulating many cellular processes; Plc1p and inositol polyphosphates are required for acetyl-CoA homeostasis which regulates global histone acetylation |
| **MRS1** | 4141 | -1.652 | -1.61 | Splicing protein; required for splicing of two mitochondrial group I introns (BI3 in COB and AI5beta in COX1); forms a splicing complex, containing four subunits of Mrs1p and two subunits of the BI3-encoded maturase, that binds to the BI3 RNA; MRS1 has a paralog, CCE1, that arose from the whole genome duplication |
| **RPL39** | 4140 | -1.637 | -2.754 | Ribosomal 60S subunit protein L39; required for ribosome biogenesis; loss of both Rpl31p and Rpl39p confers lethality; also exhibits genetic interactions with SIS1 and PAB1; homologous to mammalian ribosomal protein L39, no bacterial homolog |
| **CTK1** | 4139 | -1.634 | -1.8 | Catalytic (alpha) subunit of C-terminal domain kinase I (CTDK-I); phosphorylates both RNA pol II subunit Rpo21p to affect transcription and pre-mRNA 3' end processing, and ribosomal protein Rps2p to increase translational fidelity; required for H3K36 trimethylation but not dimethylation by Set2p; suggested stimulatory role in 80S formation during translation initiation; similar to the Drosophila dCDK12 and human CDK12 and probably CDK13 |
| **LHS1** | 4138 | -1.634 | -2.372 | Molecular chaperone of the endoplasmic reticulum lumen; involved in polypeptide translocation and folding; nucleotide exchange factor for the ER lumenal Hsp70 chaperone Kar2p; regulated by the unfolded protein response pathway |
| **RNR4** | 4137 | -1.633 | -2.334 | Ribonucleotide-diphosphate reductase (RNR) small subunit; the RNR complex catalyzes the rate-limiting step in dNTP synthesis and is regulated by DNA replication and DNA damage checkpoint pathways via localization of the small subunits; relocalizes from nucleus to cytoplasm upon DNA replication stress; RNR4 has a paralog, RNR2, that arose from the whole genome duplication |
| **GTR1** | 4136 | -1.625 | -1.611 | Subunit of a TORC1-stimulating GTPase complex; subunit of the heterodimeric Gtr1-Gtr2 GTPase complex that stimulates TORC1 in response to amino acid stimulation; tethered to the vacuolar membrane as part of the EGOC, a complex required for sorting of Gap1p and microautophagy; involved in phosphate transport and telomeric chromatin silencing; activated by the the Iml1p (GAP) subunit of the SEACIT complex; similar to human RagA and RagB |
| **MRF1** | 4135 | -1.605 | -0.852 | Mitochondrial translation release factor; involved in stop codon recognition and hydrolysis of the peptidyl-tRNA bond during mitochondrial translation; lack of MRF1 causes mitochondrial genome instability |
| **GAC1** | 4134 | -1.6 | -0.035 | Regulatory subunit for Glc7p type-1 protein phosphatase (PP1); tethers Glc7p to Gsy2p glycogen synthase, binds Hsf1p heat shock transcription factor, required for induction of some HSF-regulated genes under heat shock; GAC1 has a paralog, PIG1, that arose from the whole genome duplication |
| **VPH2** | 4133 | -1.6 | -2.136 | Integral membrane protein required for V-ATPase function; not an actual component of the vacuolar H+-ATPase (V-ATPase) complex; functions in the assembly of the V-ATPase; localized to the endoplasmic reticulum (ER); involved in methionine restriction extension of chronological lifespan in an autophagy-dependent manner |
| **CDC40** | 4131 | -1.597 | -1.031 | Pre-mRNA splicing factor; important for catalytic step II of pre-mRNA splicing and plays a role in cell cycle progression, particularly at the G1/S phase transition; required for DNA synthesis during mitosis and meiosis; has WD repeats; thermosensitivity of the cdc40 null mutant is functionally complemented by a chimeric construct containing the N-terminal 156 amino acids of yeast Cdc40p fused to the C-terminal two thirds (297 amino acids) of human CDC40 |
| **RAI1** | 4132 | -1.597 | -2.355 | Nuclear protein with decapping endonuclease activity; targets mRNAs with unmethylated 7-methylguanosine cap structures and 5'-triphosphates; binds to and stabilizes the exoribonuclease Rat1p; required for pre-rRNA processing; relocalizes to the cytosol in response to hypoxia; homologous to human DOM3Z |
| **SSZ1** | 4130 | -1.596 | -2.541 | Hsp70 protein that interacts with Zuo1p (a DnaJ homolog); interacts with Zuo1p to form a ribosome-associated complex that binds the ribosome via the Zuo1p subunit; also involved in pleiotropic drug resistance via sequential activation of PDR1 and PDR5; binds ATP |
| **CTF4** | 4129 | -1.592 | -0.75 | Chromatin-associated protein; required for sister chromatid cohesion; interacts with DNA polymerase alpha (Pol1p) and may link DNA synthesis to sister chromatid cohesion |
| **SPT10** | 4128 | -1.584 | -2.084 | Histone H3 acetylase with a role in transcriptional regulation; sequence-specific activator of histone genes, binds specifically and cooperatively to pairs of UAS elements in core histone promoters, functions at or near TATA box; involved in S phase-specific acetylation of H3K56 at histone promoters, which is required for recruitment of SWI/SNF nucleosome remodeling complex and subsequent transcription |
| **VPS1** | 4127 | -1.582 | -1.938 | Dynamin-like GTPase required for vacuolar sorting; also involved in actin cytoskeleton organization, endocytosis, late Golgi-retention of some proteins, regulation of peroxisome biogenesis |
| **NPL3** | 4125 | -1.581 | -2.174 | RNA-binding protein; promotes elongation, regulates termination, and carries poly(A) mRNA from nucleus to cytoplasm; represses translation initiation by binding eIF4G; required for pre-mRNA splicing; interacts with E3 ubiquitin ligase Bre1p, linking histone ubiquitination to mRNA processing; may have role in telomere maintenance; dissociation from mRNAs promoted by Mtr10p; phosphorylated by Sky1p in cytoplasm; protein abundance increases in response to DNA replication stress |
| **1.00 TOP** | 4126 | -1.581 | -0.219 | Topoisomerase I; nuclear enzyme that relieves torsional strain in DNA by cleaving and re-sealing the phosphodiester backbone; relaxes both positively and negatively supercoiled DNA; functions in replication, transcription, and recombination; role in processing ribonucleoside monophosphates in genomic DNA into irreversible single-strand breaks; enzymatic activity and interaction with Nsr1p are negatively regulated by polyphosphorylation |
| **RPS21A** | 4124 | -1.58 | -0.981 | Protein component of the small (40S) ribosomal subunit; homologous to mammalian ribosomal protein S21, no bacterial homolog; RPS21A has a paralog, RPS21B, that arose from the whole genome duplication |
| **RPS7A** | 4123 | -1.574 | -1.629 | Protein component of the small (40S) ribosomal subunit; interacts with Kti11p; deletion causes hypersensitivity to zymocin; homologous to mammalian ribosomal protein S7, no bacterial homolog; RPS7A has a paralog, RPS7B, that arose from the whole genome duplication |
| **MOG1** | 4122 | -1.57 | -1.846 | Conserved nuclear protein that interacts with GTP-Gsp1p; stimulates nucleotide release from Gsp1p; involved in nuclear protein import; nucleotide release is inhibited by Yrb1p |
| **NSR1** | 4121 | -1.569 | -1.414 | Nucleolar protein that binds nuclear localization sequences; required for pre-rRNA processing and ribosome biogenesis; binds to single stranded telomeric DNA and mRNA; methylated by Hmt1p; interaction with Top1p and nucleolar localization are negatively regulated by polyphosphorylation |
| **SEC72** | 4119 | -1.545 | -1.453 | Non-essential subunit of Sec63 complex; with Sec61 complex, Kar2p/BiP and Lhs1p forms a channel competent for SRP-dependent and post-translational SRP-independent protein targeting and import into the ER; other members are Sec63p, Sec62p, and Sec66p |
| **CSF1** | 4120 | -1.545 | -1.508 | Protein required for fermentation at low temperature; plays a role in the maturation of secretory proteins; the authentic, non-tagged protein is detected in highly purified mitochondria in high-throughput studies |
| **UGO1** | 4118 | -1.537 | -2.077 | Outer membrane component of the mitochondrial fusion machinery; binds to Fzo1p and Mgm1p to link these two GTPases during mitochondrial fusion; involved in fusion of both the outer and inner membranes; facilitates dimerization of Fzo1p during fusion; import into the outer membrane is mediated by Tom70p and Mim1p; has similarity to carrier proteins but likely not a transporter; similar to human SLC25A46 implicated in optic atroprophy spectrum disorder |
| **PIH1** | 4117 | -1.518 | -0.808 | Component of the conserved R2TP complex (Rvb1-Rvb2-Tah1-Pih1); R2TP complex interacts with Hsp90 (Hsp82p and Hsc82p) to mediate assembly large protein complexes such as box C/D snoRNPs and RNA polymerase II |
| **RUP1** | 4116 | -1.513 | 0.084 | Protein that regulates ubiquitination of Rsp5p; has a WW domain consensus motif of PPPSY (residues 131-135) that mediates binding of Rsp5p to Ubp2p; contains an UBA domain; relative distribution to the nucleus increases upon DNA replication stress |
| **PTK2** | 4115 | -1.502 | -0.646 | Serine/threonine protein kinase; involved in regulation of ion transport across plasma membrane; carboxyl terminus is essential for glucose-dependent Pma1p activation via phosphorylation of Pma1p-Ser899; enhances spermine uptake; PTK2 has a paralog, PTK1, that arose from the whole genome duplication |
| **FCY22** | 4114 | -1.479 | -0.978 | Putative purine-cytosine permease; very similar to Fcy2p but cannot substitute for its function |
| **GND1** | 4113 | -1.473 | -1.561 | 6-phosphogluconate dehydrogenase (decarboxylating); catalyzes an NADPH regenerating reaction in the pentose phosphate pathway; required for growth on D-glucono-delta-lactone and adaptation to oxidative stress; GND1 has a paralog, GND2, that arose from the whole genome duplication |
| **IES6** | 4112 | -1.471 | -2.0 | Component of the INO80 chromatin remodeling complex; critical for INO80 function; involved in regulation of chromosome segregation and maintenance of normal centromeric chromatin structure; human ortholog INO80C is a member of the human INO80 complex; implicated in DNA repair based on genetic interactions with RAD52 epistasis genes |
| **RPL42A** | 4111 | -1.461 | 0.215 | Ribosomal 60S subunit protein L42A; homologous to mammalian ribosomal protein L36A, no bacterial homolog; RPL42A has a paralog, RPL42B, that arose from the whole genome duplication |
| **RPS6A** | 4110 | -1.456 | -1.594 | Protein component of the small (40S) ribosomal subunit; homologous to mammalian ribosomal protein S6, no bacterial homolog; phosphorylated on S233 by Ypk3p in a TORC1-dependent manner, and on S232 in a TORC1/2-dependent manner by Ypk1/2/3p; RPS6A has a paralog, RPS6B, that arose from the whole genome duplication |
| **VMA7** | 4109 | -1.453 | -2.325 | Subunit F of the V1 peripheral membrane domain of V-ATPase; part of the electrogenic proton pump found throughout the endomembrane system; required for the V1 domain to assemble onto the vacuolar membrane; the V1 peripheral membrane domain of vacuolar H+-ATPase (V-ATPase) has eight subunits |
| **SUV3** | 4108 | -1.442 | -1.152 | ATP-dependent RNA helicase; component of the mitochondrial degradosome along with the RNase Dss1p; the degradosome associates with the ribosome and mediates RNA turnover; also required during splicing of the COX1 AI5_beta intron; expression of a processed form of human homolog SUPV3L1 carrying an N-terminal deletion of 46 amino acids rescues yeast suv3 null mutant |
| **THR4** | 4107 | -1.44 | -1.301 | Threonine synthase; conserved protein that catalyzes formation of threonine from O-phosphohomoserine; expression is regulated by the GCN4-mediated general amino acid control pathway |
| **RPL12B** | 4106 | -1.436 | -1.981 | Ribosomal 60S subunit protein L12B; rpl12a rpl12b double mutant exhibits slow growth and slow translation; homologous to mammalian ribosomal protein L12 and bacterial L11; RPL12B has a paralog, RPL12A, that arose from the whole genome duplication |
| **BUD27** | 4105 | -1.427 | -1.606 | Unconventional prefoldin protein involved in translation initiation; required for correct assembly of RNAP I, II, and III in an Rpb5p-dependent manner; shuttles between nucleus and cytoplasm; mutants have inappropriate expression of nutrient sensitive genes due to translational derepression of Gcn4p transcription factor; diploid mutants show random budding; ortholog of human URI/RMP |
| **EOS1** | 4103 | -1.425 | -1.297 | Protein involved in N-glycosylation; deletion mutation confers sensitivity to exidative stress and shows synthetic lethality with mutations in the spindle checkpoint genes BUB3 and MAD1; YNL080C is not an essential gene |
| **SWD1** | 4104 | -1.425 | -0.695 | Subunit of the COMPASS (Set1C) complex; COMPASS methylates histone H3 on lysine 4 and is required in transcriptional silencing near telomeres; WD40 beta propeller superfamily member with similarity to mammalian Rbbp7 |
| **FYV6** | 4102 | -1.42 | -2.843 | hypothetical protein; required for survival upon exposure to K1 killer toxin; proposed to regulate double-strand break repair via non-homologous end-joining |
| **SOD1** | 4101 | -1.414 | -0.707 | Cytosolic copper-zinc superoxide dismutase; detoxifies superoxide; stabilizes Yck1p and Yck2p kinases in glucose to repress respiration; phosphorylated by Dun1p, enters nucleus under oxidative stress to promote transcription of stress response genes; human ortholog SOD1 implicated in ALS complements a null allele; abundance increases under DNA replication stress and during exposure to boric acid; localization to mitochondrial intermembrane space is modulated by MICOS complex |
| **PRS5** | 4100 | -1.41 | -1.159 | 5-phospho-ribosyl-1(alpha)-pyrophosphate synthetase; synthesizes PRPP, which is required for nucleotide, histidine, and tryptophan biosynthesis; one of five related enzymes, which are active as heteromultimeric complexes; forms cytoplasmic foci upon DNA replication stress |
| **RAD6** | 4099 | -1.404 | -1.666 | Ubiquitin-conjugating enzyme (E2); involved in postreplication repair as a heterodimer with Rad18p, regulation of K63 polyubiquitination in response to oxidative stress, DSBR and checkpoint control as a heterodimer with Bre1p, ubiquitin-mediated N-end rule protein degradation as a heterodimer with Ubr1p, ERAD with Ubr1p in the absence of canonical ER membrane ligases, and Rpn4p turnover as part of proteasome homeostasis, in complex with Ubr2p and Mub1p |
| **HOM3** | 4098 | -1.4 | -0.607 | Aspartate kinase (L-aspartate 4-P-transferase); cytoplasmic enzyme that catalyzes the first step in the common pathway for methionine and threonine biosynthesis; expression regulated by Gcn4p and the general control of amino acid synthesis |
| **GIM5** | 4097 | -1.381 | -1.735 | Subunit of the heterohexameric cochaperone prefoldin complex; prefoldin binds specifically to cytosolic chaperonin and transfers target proteins to it; prefoldin complex also localizes to chromatin of actively transcribed genes in the nucleus and facilitates transcriptional elongation |
| **HIT1** | 4096 | -1.37 | -1.801 | Protein involved in C/D snoRNP assembly; regulates abundance of Rsa1p; required for growth at high temperature; similar to human ZNHIT3 |
| **RPL31A** | 4095 | -1.369 | -2.265 | Ribosomal 60S subunit protein L31A; associates with karyopherin Sxm1p; loss of both Rpl31p and Rpl39p confers lethality; homologous to mammalian ribosomal protein L31, no bacterial homolog; RPL31A has a paralog, RPL31B, that arose from the whole genome duplication |
| **ELM1** | 4094 | -1.366 | -0.565 | Serine/threonine protein kinase; regulates the orientation checkpoint, the morphogenesis checkpoint and the metabolic switch from fermentative to oxidative metabolism by phosphorylating the activation loop of Kin4p, Hsl1p and Snf4p respectively; cooperates with Hsl7p in recruiting Hsl1p to the septin ring, a prerequisite for subsequent recruitment, phosphorylation, and degradation of Swe1p; forms part of the bud neck ring; regulates cytokinesis |
| **DOA4** | 4093 | -1.362 | -0.211 | Ubiquitin hydrolase; deubiquitinates intralumenal vesicle (ILVs) cargo proteins; required for recycling ubiquitin from proteasome-bound ubiquitinated intermediates, acts at the late endosome/prevacuolar compartment to recover ubiquitin from ubiquitinated membrane proteins destined for the vacuole; DOA4 has a paralog, UBP5, that arose from the whole genome duplication |
| **RPL37A** | 4092 | -1.361 | -1.766 | Ribosomal 60S subunit protein L37A; required for processing of 27SB pre-rRNA and formation of stable 66S assembly intermediates; homologous to mammalian ribosomal protein L37, no bacterial homolog; RPL37A has a paralog, RPL37B, that arose from the whole genome duplication |
| **AIR1** | 4091 | -1.358 | -0.176 | Zinc knuckle protein; involved in nuclear RNA processing and degradation as a component of the TRAMP complex; stimulates the poly(A) polymerase activity of Pap2p in vitro; AIR1 has a paralog, AIR2, that arose from the whole genome duplication; although Air1p and Air2p are homologous TRAMP subunits, they have nonredundant roles in regulation of substrate specificity of the exosome |
| **YDL133W** | 4090 | -1.356 | 0.026 | Regulator of phospholipase D (Spo14p); interacts with Spo14p and regulates its catalytic activity; capable of buffering the toxicity of C16:0 platelet activating factor, a lipid that accumulates intraneuronally in Alzheimer's patients |
| **RFX1** | 4089 | -1.353 | -0.551 | Major transcriptional repressor of DNA-damage-regulated genes; recruits repressors Tup1p and Cyc8p to their promoters; involved in DNA damage and replication checkpoint pathway; similar to a family of mammalian DNA binding RFX1-4 proteins |
| **SAT4** | 4088 | -1.352 | -0.871 | Ser/Thr protein kinase involved in salt tolerance; funtions in regulation of Trk1p-Trk2p potassium transporter; overexpression affects the Fe-S and lipoamide containing proteins in the mitochondrion; required for lipoylation of Lat1p, Kgd2p and Gcv3p; partially redundant with Hal5p; has similarity to Npr1p; localizes to the cytoplasm and mitochondrion |
| **SWI6** | 4086 | -1.349 | -2.301 | Transcription cofactor; forms complexes with Swi4p and Mbp1p to regulate transcription at the G1/S transition; involved in meiotic gene expression; also binds Stb1p to regulate transcription at START; cell wall stress induces phosphorylation by Mpk1p, which regulates Swi6p localization; required for the unfolded protein response, independently of its known transcriptional coactivators |
| **LAG1** | 4087 | -1.349 | 0.883 | Ceramide synthase component; involved in synthesis of ceramide from C26(acyl)-coenzyme A and dihydrosphingosine or phytosphingosine, functionally equivalent to Lac1p; forms ER foci upon DNA replication stress; homolog of human CERS2, a tumor metastasis suppressor gene whose silencing enhances invasion/metastasis of prostate cancer cells; LAG1 has a paralog, LAC1, that arose from the whole genome duplication |
| **RPA49** | 4085 | -1.343 | -1.994 | RNA polymerase I subunit A49; essential for nucleolar assembly and for high polymerase loading rate; required for nucleolar localization of Rpa34p |
| **APS2** | 4084 | -1.343 | 0.006 | Small subunit of the clathrin-associated adaptor complex AP-2; AP-2 is involved in protein sorting at the plasma membrane; related to the sigma subunit of the mammalian plasma membrane clathrin-associated protein (AP-2) complex |
| **STP22** | 4083 | -1.342 | 0.209 | Component of the ESCRT-I complex; ESCRT-I is involved in ubiquitin-dependent sorting of proteins into the endosome; prevents polyubiquitination of the arrestin-related protein Rim8p, thereby directing its monoubiquitination by Rsp5p; homologous to the mouse and human Tsg101 tumor susceptibility gene; mutants exhibit a Class E Vps phenotype |
| **DHH1** | 4082 | -1.341 | -0.868 | Cytoplasmic DEAD-box helicase, stimulates mRNA decapping; coordinates distinct steps in mRNA function and decay, interacting with both decapping and deadenylase complexes; role in translational repression, mRNA decay, and possibly mRNA export; interacts and cooperates with Ngr1p to promote specific mRNA decay; ATP- and RNA-bound form promotes processing body (PB) assembly, while ATPase stimulation by Not1p promotes PB disassembly; forms cytoplasmic foci on replication stress |
| **SNF4** | 4081 | -1.34 | -1.21 | Activating gamma subunit of the AMP-activated Snf1p kinase complex; additional subunits of the complex are Snf1p and a Sip1p/Sip2p/Gal83p family member; activates glucose-repressed genes, represses glucose-induced genes; role in sporulation, and peroxisome biogenesis; protein abundance increases in response to DNA replication stress |
| **LST4** | 4080 | -1.338 | -1.287 | Subunit of the Lst4p-Lst7p GTPase activating protein complex for Gtr2p; stimulates the GTPase activity of Rag family GTPase Gtr2p, within the context of the Gtr1p-Gtr2p heterodimer, after amino acid stimulation; required for activation of TORC1 in response to amino acid stimulation; recruited to the vacuolar membrane during amino acid starvation and released from the membrane by TORC1; required for the transport of amino acid permease Gap1p from the Golgi to the cell surface |
| **RPL21B** | 4079 | -1.332 | -0.512 | Ribosomal 60S subunit protein L21B; homologous to mammalian ribosomal protein L21, no bacterial homolog; RPL21B has a paralog, RPL21A, that arose from the whole genome duplication |
| **SER1** | 4078 | -1.33 | -0.841 | 3-phosphoserine aminotransferase; catalyzes the formation of phosphoserine from 3-phosphohydroxypyruvate, required for serine and glycine biosynthesis; regulated by the general control of amino acid biosynthesis mediated by Gcn4p; protein abundance increases in response to DNA replication stress |
| **VMA8** | 4077 | -1.324 | -1.574 | Subunit D of the V1 peripheral membrane domain of V-ATPase; part of the electrogenic proton pump found throughout the endomembrane system; plays a role in the coupling of proton transport and ATP hydrolysis; the V1 peripheral membrane domain of the vacuolar H+-ATPase (V-ATPase) has eight subunits |
| **VPS45** | 4076 | -1.314 | -1.171 | Protein of the Sec1p/Munc-18 family; essential for vacuolar protein sorting; required for the function of Pep12p and the early endosome/late Golgi SNARE Tlg2p; essential for fusion of Golgi-derived vesicles with the prevacuolar compartment |
| **IKS1** | 4074 | -1.307 | -0.41 | Protein kinase of unknown cellular role; putative serine/threonine kinase; expression is induced during mild heat stress; deletion mutants are hypersensitive to copper sulphate and resistant to sorbate; interacts with an N-terminal fragment of Sst2p |
| **HIS5** | 4075 | -1.307 | 0.035 | Histidinol-phosphate aminotransferase; catalyzes the seventh step in histidine biosynthesis; responsive to general control of amino acid biosynthesis; mutations cause histidine auxotrophy and sensitivity to Cu, Co, and Ni salts |
| **RTT109** | 4072 | -1.305 | -1.422 | Histone acetyltransferase; critical for cell survival in presence of DNA damage during S phase, required for recovery after DSB repair; acetylates H3K56, H3K9; H3K56 acetylation activity required for expression homeostasis, buffering of mRNA synthesis rate against changes in gene dosage during S phase; involved in non-homologous end joining and regulation of Ty1 transposition; prevents hyper-amplification of rDNA; interacts physically with Vps75p |
| **YGR042W** | 4073 | -1.305 | 0.087 | hypothetical protein; involved in maintenance of proper telomere length; green fluorescent protein (GFP)-fusion protein localizes to both the cytoplasm and the nucleus; forms nuclear foci upon DNA replication stress |
| **SSF1** | 4071 | -1.303 | -0.818 | Constituent of 66S pre-ribosomal particles; required for ribosomal large subunit maturation; functionally redundant with Ssf2p; member of the Brix family; SSF1 has a paralog, SSF2, that arose from the whole genome duplication |
| **RPL6B** | 4070 | -1.3 | -1.811 | Ribosomal 60S subunit protein L6B; binds 5.8S rRNA; homologous to mammalian ribosomal protein L6, no bacterial homolog; RPL6B has a paralog, RPL6A, that arose from the whole genome duplication |
| **LYS1** | 4069 | -1.299 | -1.462 | Saccharopine dehydrogenase (NAD+, L-lysine-forming); catalyzes the conversion of saccharopine to L-lysine, which is the final step in the lysine biosynthesis pathway; also has mRNA binding activity |
| **SAC1** | 4068 | -1.292 | -1.83 | Phosphatidylinositol phosphate (PtdInsP) phosphatase; involved in hydrolysis of PtdIns[4]P in the early and medial Golgi; regulated by interaction with Vps74p; ER localized transmembrane protein which cycles through the Golgi; involved in protein trafficking and processing, secretion, and cell wall maintenance; regulates sphingolipid biosynthesis through the modulation of PtdIns(4)P metabolism |
| **RPS24A** | 4066 | -1.291 | -1.058 | Protein component of the small (40S) ribosomal subunit; homologous to mammalian ribosomal protein S24, no bacterial homolog; RPS24A has a paralog, RPS24B, that arose from the whole genome duplication |
| **SEC66** | 4067 | -1.291 | -1.949 | Non-essential subunit of Sec63 complex; with Sec61 complex, Kar2p/BiP and Lhs1p forms a channel competent for SRP-dependent and post-translational SRP-independent protein targeting and import into the ER; other members are Sec63p, Sec62p, and Sec72p |
| **GLR1** | 4065 | -1.288 | 0.573 | Cytosolic and mitochondrial glutathione oxidoreductase; converts oxidized glutathione to reduced glutathione; cytosolic Glr1p is the main determinant of the glutathione redox state of the mitochondrial intermembrane space; mitochondrial Glr1p has a role in resistance to hyperoxia; protein abundance increases in response to DNA replication stress |
| **TPS1** | 4064 | -1.287 | -1.499 | Synthase subunit of trehalose-6-P synthase/phosphatase complex; synthesizes the storage carbohydrate trehalose, which is critically important for survival of long-term desiccation; also found in a monomeric form; expression is induced by the stress response and repressed by the Ras-cAMP pathway; protein abundance increases in response to DNA replication stress and in response to prolonged exposure to boric acid |
| **LGE1** | 4063 | -1.285 | -0.867 | hypothetical protein; null mutant forms abnormally large cells, and homozygous diploid null mutant displays delayed premeiotic DNA synthesis and reduced efficiency of meiotic nuclear division |
| **SLM6** | 4061 | -1.278 | -0.581 | No Description Found |
| **VPS36** | 4062 | -1.278 | -0.587 | Component of the ESCRT-II complex; contains the GLUE (GRAM Like Ubiquitin binding in EAP45) domain which is involved in interactions with ESCRT-I and ubiquitin-dependent sorting of proteins into the endosome; plays a role in the formation of mutant huntingtin (Htt) aggregates in yeast |
| **HFI1** | 4060 | -1.277 | -2.639 | Adaptor protein required for structural integrity of the SAGA complex; a histone acetyltransferase-coactivator complex that is involved in global regulation of gene expression through acetylation and transcription functions |
| **SRN2** | 4059 | -1.273 | 0.061 | Component of the ESCRT-I complex; ESCRT-I is involved in ubiquitin-dependent sorting of proteins into the endosome; suppressor of rna1-1 mutation; may be involved in RNA export from nucleus |
| **RPS27A** | 4058 | -1.272 | 0.176 | Protein component of the small (40S) ribosomal subunit; homologous to mammalian ribosomal protein S27, no bacterial homolog; RPS27A has a paralog, RPS27B, that arose from the whole genome duplication; protein abundance increases in response to DNA replication stress |
| **THO2** | 4057 | -1.269 | -0.496 | Subunit of the THO complex; THO is required for efficient transcription elongation and involved in transcriptional elongation-associated recombination; required for LacZ RNA expression from certain plasmids |
| **NUP2** | 4056 | -1.269 | 1.139 | Nucleoporin involved in nucleocytoplasmic transport; binds to either the nucleoplasmic or cytoplasmic faces of the nuclear pore complex depending on Ran-GTP levels; also has a role in chromatin organization |
| **SER2** | 4053 | -1.267 | -1.344 | Phosphoserine phosphatase of the phosphoglycerate pathway; involved in serine and glycine biosynthesis, expression is regulated by the available nitrogen source |
| **DLD1** | 4055 | -1.267 | -0.07 | Major mitochondrial D-lactate dehydrogenase; oxidizes D-lactate to pyruvate, transcription is heme-dependent, repressed by glucose, and derepressed in ethanol or lactate; located in the mitochondrial inner membrane |
| **YSY6** | 4054 | -1.267 | -0.607 | hypothetical protein; expression suppresses a secretory pathway mutation in E. coli; has similarity to the mammalian RAMP4 protein involved in secretion |
| **URM1** | 4052 | -1.263 | -1.341 | Ubiquitin-like protein involved in thiolation of cytoplasmic tRNAs; receives sulfur from the E1-like enzyme Uba4p and transfers it to tRNA; also functions as a protein tag with roles in nutrient sensing and oxidative stress response |
| **RSM7** | 4051 | -1.26 | -1.879 | Mitochondrial ribosomal protein of the small subunit; has similarity to E. coli S7 ribosomal protein |
| **CGI121** | 4050 | -1.258 | -0.822 | Component of the EKC/KEOPS complex; EKC/KEOPS complex is required for t6A tRNA modification and telomeric TG1-3 recombination; may have role in transcription; Cgi121p is dispensable for tRNA modification; other complex members are Bud32p, Kae1p, Pcc1p, and Gon7p |
| **FLX1** | 4049 | -1.253 | -0.235 | Mitochondrial flavin adenine dinucleotide transporter; FAD is a synthesis product of riboflavin; human homolog SLC25A32 is implicated in multiple acyl-CoA dehydrogenase deficiency (MADD) or glutaric aciduria type II (GAII), and can complement yeast null mutant |
| **OCA1** | 4048 | -1.249 | -0.728 | Putative protein tyrosine phosphatase; required for cell cycle arrest in response to oxidative damage of DNA |
| **DOC1** | 4047 | -1.244 | -1.207 | Processivity factor; required for the ubiquitination activity of the anaphase promoting complex (APC), mediates the activity of the APC by contributing to substrate recognition; involved in cyclin proteolysis; contains a conserved DOC1 homology domain |
| **DID2** | 4046 | -1.243 | -0.149 | Class E protein of the vacuolar protein-sorting (Vps) pathway; binds Vps4p and directs it to dissociate ESCRT-III complexes; forms a functional and physical complex with Ist1p; human ortholog may be altered in breast tumors |
| **GLN3** | 4045 | -1.243 | -0.667 | Transcriptional activator of genes regulated by nitrogen catabolite repression; localization and activity regulated by quality of nitrogen source and Ure2p |
| **TRM7** | 4044 | -1.241 | -0.301 | 2'-O-ribose methyltransferase; methylates the 2'-O-ribose of tRNA-Phe, tRNA-Trp, and tRNA-Leu at positions C32 and N34 of tRNA anticodon loop; crucial biological role likely modification of tRNA-Phe; interacts with Trm732p and Rtt10p in 2'-O-methylation of C32 and N34 substrate tRNAs, respectively; yeast null mutant can be functionally complemented by human FTSJ1, mutations in which have been implicated in nonsyndromic X-linked intellectual disability (NSXLID) |
| **ARG3** | 4043 | -1.238 | -1.756 | Ornithine carbamoyltransferase; also known as carbamoylphosphate:L-ornithine carbamoyltransferase; catalyzes the biosynthesis of the arginine precursor citrulline |
| **MMP1** | 4042 | -1.234 | -0.389 | High-affinity S-methylmethionine permease; required for utilization of S-methylmethionine as a sulfur source; has similarity to S-adenosylmethionine permease Sam3p |
| **CBC2** | 4040 | -1.233 | -1.296 | Small subunit of the heterodimeric cap binding complex with Sto1p; interacts with Npl3p, possibly to package mRNA for export from the nucleus; may have a role in telomere maintenance; contains an RNA-binding motif |
| **RPS0B** | 4041 | -1.233 | -0.988 | Protein component of the small (40S) ribosomal subunit; RPS0B has a paralog, RPS0A, that arose from the whole genome duplication; required for maturation of 18S rRNA along with Rps0Ap; deletion of either RPS0 gene reduces growth rate, deletion of both genes is lethal; homologous to human ribosomal protein SA and bacterial S2 |
| **ALF1** | 4039 | -1.232 | -1.19 | Alpha-tubulin folding protein; similar to mammalian cofactor B; Alf1p-GFP localizes to cytoplasmic microtubules; required for the folding of alpha-tubulin and may play an additional role in microtubule maintenance |
| **4.00 SIT** | 4038 | -1.231 | -2.428 | Ceramide-activated, type 2A-related serine-threonine phosphatase; functions in G1/S transition of mitotic cycle; controls lifespan, mitochondrial function, cell cycle progression by regulating HXK2 phosphorylation; regulator of COPII coat dephosphorylation; required for ER to Golgi traffic; interacts with Hrr25p kinase; cytoplasmic and nuclear protein that modulates functions mediated by Pkc1p including cell wall and actin cytoskeleton organization; similar to human PP6 |
| **MET5** | 4037 | -1.231 | 1.113 | Sulfite reductase beta subunit; involved in amino acid biosynthesis, transcription repressed by methionine |
| **RPS23A** | 4034 | -1.23 | -0.885 | Ribosomal protein 28 (rp28) of the small (40S) ribosomal subunit; required for translational accuracy; homologous to mammalian ribosomal protein S23 and bacterial S12; RPS23A has a paralog, RPS23B, that arose from the whole genome duplication; deletion of both RPS23A and RPS23B is lethal |
| **BUD20** | 4036 | -1.23 | -1.928 | C2H2-type zinc finger protein required for ribosome assembly; shuttling factor which associates with pre-60S particles in the nucleus, accompanying them to the cytoplasm; cytoplasmic dissociation of Bud20p requires Drg1p; N-terminus harbors a nuclear localization signal (NLS) and a nuclear export signal (NES); cytoplasmic Bud20p is reimported by Kap123-dependent pathway; involved in bud-site selection; diploid mutants display a random budding pattern; similar to human ZNF593 |
| **HIS6** | 4035 | -1.23 | -0.124 | Enzyme that catalyzes the fourth step in the histidine pathway; Phosphoribosylformimino-5-aminoimidazole carboxamide ribotide isomerase; mutations cause histidine auxotrophy and sensitivity to Cu, Co, and Ni salts |
| **PAR32** | 4033 | -1.226 | -1.285 | hypothetical protein; hyperphosphorylated upon rapamycin treatment in a Tap42p-dependent manner; green fluorescent protein (GFP)-fusion protein localizes to the cytoplasm; PAR32 is not an essential gene |
| **RAD52** | 4031 | -1.218 | -1.035 | Protein that stimulates strand exchange; stimulates strand exchange by facilitating Rad51p binding to single-stranded DNA; anneals complementary single-stranded DNA; involved in the repair of double-strand breaks in DNA during vegetative growth and meiosis and UV induced sister chromatid recombination |
| **YCL001W-A** | 4032 | -1.218 | -0.19 | hypothetical protein; YCL001W-A gene has similarity to DOM34 and is present in a region duplicated between chromosomes XIV and III |
| **GLY1** | 4030 | -1.209 | -1.637 | Threonine aldolase; catalyzes the cleavage of L-allo-threonine and L-threonine to glycine; involved in glycine biosynthesis |
| **YJL070C** | 4029 | -1.205 | -0.295 | Putative metallo-dependent hydrolase superfamily protein; similar to AMP deaminases but lacks key catalytic residues and does not rescue purine nucleotide metabolic defect of quadruple aah1 ade8 amd1 his1 mutant; may regulate purine nucleotide homeostasis as overexpression in an AMD1 strain grown in adenine results in greatly reduced GDP and GTP intracellular levels; not an essential gene; YJL070C has a paralog, YBR284W, that arose from the whole genome duplication |
| **RPL20A** | 4027 | -1.203 | -1.543 | Ribosomal 60S subunit protein L20A; homologous to mammalian ribosomal protein L18A, no bacterial homolog; RPL20A has a paralog, RPL20B, that arose from the whole genome duplication |
| **WSS1** | 4028 | -1.203 | -0.451 | SUMO-ligase and SUMO-targeted metalloprotease; involved in DNA repair; removes DNA-protein crosslinks at stalled replication forks during replication of damaged DNA; clears chromatin-bound sumoylated proteins; localizes to single spot on nuclear periphery of mother cells but not daughters; exhibits vacuolar localization upon genotoxic stress; activated by DNA binding; member of minigluzincins protease family with mammalian DVC1/Spartan |

**SUPPLEMENTARY TABLE S7: Failing Genes in Betaxanthin Screen**

| **gene** | **Yield_Rank** | **Yield_Score** | **Size_Score** | **Description** |
| --- | --- | --- | --- | --- |
| **YOR186W** | na | na | na | hypothetical protein; proper regulation of expression during heat stress is sphingolipid-dependent; mCherry fusion protein localizes to the vacuole; YOR186W has a paralog, YLR297W, that arose from the whole genome duplication |
| **STE11** | na | na | na | Signal transducing MEK kinase; involved in pheromone response and pseudohyphal/invasive growth pathways where it phosphorylates Ste7p, and the high osmolarity response pathway, via phosphorylation of Pbs2p; regulated by Ste20p and Ste50p; protein abundance increases in response to DNA replication stress |
| **YHR177W** | na | na | na | Putative transcription factor containing a WOPR domain; binds DNA in vitro; similar to C. albicans Wor1p transcription factor that regulates white-opaque switching; overexpression causes a cell cycle delay or arrest |
| **FUS3** | na | na | na | Mitogen-activated serine/threonine protein kinase involved in mating; phosphoactivated by Ste7p; substrates include Ste12p, Far1p, Bni1p, Sst2p; inhibits invasive growth during mating by phosphorylating Tec1p, promoting its; inhibits recruitment of Ste5p, Cdc42p-mediated asymmetry and mating morphogenesis |
| **KAR4** | na | na | na | Transcription factor required for response to pheromones; also required during meiosis; exists in two forms, a slower-migrating form more abundant during vegetative growth and a faster-migrating form induced by pheromone |
| **RIB1** | na | na | na | GTP cyclohydrolase II; catalyzes the first step of the riboflavin biosynthesis pathway |
| **DSE2** | na | na | na | Daughter cell-specific secreted protein with similarity to glucanases; degrades cell wall from the daughter side causing daughter to separate from mother; expression is repressed by cAMP |
| **IMP2'** | na | na | na | Transcriptional activator involved in maintenance of ion homeostasis; also involved in protection against DNA damage caused by bleomycin and other oxidants; contains a C-terminal leucine-rich repeat |
| **CDC26** | na | na | na | Subunit of the Anaphase-Promoting Complex/Cyclosome (APC/C); which is a ubiquitin-protein ligase required for degradation of anaphase inhibitors, including mitotic cyclins, during the metaphase/anaphase transition; relocalizes to the cytosol in response to hypoxia |
| **TIR3** | na | na | na | Cell wall mannoprotein; member of Srp1p/Tip1p family of serine-alanine-rich proteins; expressed under anaerobic conditions and required for anaerobic growth; TIR3 has a paralog, TIR2, that arose from the whole genome duplication |
| **MGT1** | na | na | na | DNA repair methyltransferase (6-O-methylguanine-DNA methylase); involved in protection against DNA alkylation damage |
| **YOR105W** | na | na | na | hypothetical protein; expressed at both mRNA and protein levels |
| **FYV7** | na | na | na | Essential protein required for maturation of 18S rRNA; required for survival upon exposure to K1 killer toxin |
| **CEX1** | na | na | na | Component of nuclear aminoacylation-dependent tRNA export pathway; cytoplasmic; interacts with nuclear pore component Nup116p; copurifies with tRNA export receptors Los1p and Msn5p, as well as eIF-1a; required for activation of RAN GTPase Gsp1p and dissociation of receptor-tRNA-Gsp1p export complex; recruits Rna1p from cytoplasm to NPC, facilitates Rna1p activation of Gsp1p GTPase activity by enabling Rna1p to gain access to Gsp1p-GTP bound to export receptor tRNA complex |
| **YFL035C-B** | na | na | na | No Description Found |
| **RPS24B** | na | na | na | Protein component of the small (40S) ribosomal subunit; homologous to mammalian ribosomal protein S24, no bacterial homolog; RPS24B has a paralog, RPS24A, that arose from the whole genome duplication |
| **YOR314W** | na | na | na | hypothetical protein; conserved across S. cerevisiae strains |
| **MNI1** | na | na | na | No Description Found |
| **MFA1** | na | na | na | Mating pheromone a-factor; made by a cells; interacts with alpha cells to induce cell cycle arrest and other responses leading to mating; biogenesis involves C-terminal modification, N-terminal proteolysis, and export; also encoded by MFA2 |
| **STE20** | na | na | na | Cdc42p-activated signal transducing kinase; involved in pheromone response, pseudohyphal/invasive growth, vacuole inheritance, down-regulation of sterol uptake; GBB motif binds Ste4p; member of the PAK (p21-activated kinase) family |
| **CLC1** | na | na | na | Clathrin light chain; subunit of the major coat protein involved in intracellular protein transport and endocytosis; regulates endocytic progression; thought to regulate clathrin function; the clathrin triskelion is a trimeric molecule composed of three heavy chains that radiate from a vertex and three light chains which bind noncovalently near the vertex of the triskelion |
| **MBB1** | na | na | na | hypothetical protein; conserved among S. cerevisiae strains, not conserved in closely related Saccharomyces species; protein detected in large-scale protein-protein interaction studies; YJL199C is not an essential gene |
| **HTL1** | na | na | na | Component of the RSC chromatin remodeling complex; RSC functions in transcriptional regulation and elongation, chromosome stability, and establishing sister chromatid cohesion; involved in telomere maintenance |
| **ERG4** | na | na | na | C-24(28) sterol reductase; catalyzes the final step in ergosterol biosynthesis; mutants are viable, but lack ergosterol |
| **DIG1** | na | na | na | MAP kinase-responsive inhibitor of the Ste12p transcription factor; involved in the regulation of mating-specific genes and the invasive growth pathway; related regulators Dig1p and Dig2p bind to Ste12p; DIG1 has a paralog, DIG2, that arose from the whole genome duplication |
| **TUM1** | na | na | na | Rhodanese domain sulfur transferase; accepts persulfite from Nfs1p and transfers it to Uba4p in the pathway for 2-thiolation of the wobble uridine base of tRNAs; also stimulates sulfur transfer by Nfs1p; may be mitochondrially localized |
| **PEP7** | na | na | na | Adaptor protein involved in vesicle-mediated vacuolar protein sorting; multivalent adaptor protein; facilitates vesicle-mediated vacuolar protein sorting by ensuring high-fidelity vesicle docking and fusion, which are essential for targeting of vesicles to the endosome; required for vacuole inheritance |
| **QDR2** | na | na | na | Plasma membrane transporter of the major facilitator superfamily; member of the 12-spanner drug:H(+) antiporter DHA1 family; exports copper; has broad substrate specificity and can transport many mono- and divalent cations; transports a variety of drugs and is required for resistance to quinidine, barban, cisplatin, and bleomycin; contributes to potassium homeostasis; expression is regulated by copper |
| **FUN12** | na | na | na | Translation initiation factor eIF5B; GTPase that promotes Met-tRNAiMet binding to ribosomes and ribosomal subunit joining; promotes GTP-dependent maturation of 18S rRNA by Nob1p; protein abundance increases in response to DNA replication stress; homolog of bacterial IF2 |
| **SIR4** | na | na | na | SIR protein involved in assembly of silent chromatin domains; silent information regulator (SIR) along with SIR2 and SIR3; involved in assembly of silent chromatin domains at telomeres and the silent mating-type loci; some alleles of SIR4 prolong lifespan; required for telomere hypercluster formation in quiescent yeast cells |
| **PAF1** | na | na | na | Component of the Paf1p complex involved in transcription elongation; binds to and modulates the activity of RNA polymerases I and II; required for expression of a subset of genes, including cell cycle-regulated genes; involved in SER3 repression by helping to maintain SRG1 transcription-dependent nucleosome occupancy; homolog of human PD2/hPAF1 |
| **CST26** | na | na | na | Acyltransferase; enzyme mainly responsible for the introduction of saturated very long chain fatty acids into neo-synthesized molecules of phosphatidylinositol; required for incorporation of stearic acid into phosphatidylinositol; affects chromosome stability when overexpressed; CST26 has a paralog, YDR018C, that arose from the whole genome duplication |
| **AXL1** | na | na | na | Haploid specific endoprotease of a-factor mating pheromone; performs one of two N-terminal cleavages during maturation of a-factor mating pheromone; required for axial budding pattern of haploid cells |
| **NCR1** | na | na | na | Vacuolar membrane protein; transits through the biosynthetic vacuolar protein sorting pathway, involved in sphingolipid metabolism; cells lacking Ncr1p exhibit high levels of long chain bases (LCB), similar to the accumulation of high amounts of lipids observed in patients with Neimann-Pick C, a disease caused by loss-of-function mutations in NPC1, the functional ortholog of Ncr1p |
| **SHS1** | na | na | na | Component of the septin ring that is required for cytokinesis; present at the ends of rod-like septin hetero-oligomers; C-terminal extension is important for recruitment of Bni5p to the mother-bud neck, which in turn is required for Myo1p recruitment and cytokinesis; undergoes sumoylation and phosphorylation during mitosis; protein abundance increases in response to DNA replication stress |
| **CYC8** | na | na | na | General transcriptional co-repressor; acts together with Tup1p; also acts as part of a transcriptional co-activator complex that recruits the SWI/SNF and SAGA complexes to promoters; can form the prion [OCT+] |
| **REV1** | na | na | na | Deoxycytidyl transferase; involved in repair of abasic sites and adducted guanines in damaged DNA by translesion synthesis (TLS); forms a complex with the subunits of DNA polymerase zeta, Rev3p and Rev7p; relocalizes from nucleus to cytoplasm upon DNA replication stress |
| **VPS38** | na | na | na | Part of a Vps34p phosphatidylinositol 3-kinase complex; functions in carboxypeptidase Y (CPY) sorting; binds Vps30p and Vps34p to promote production of phosphatidylinositol 3-phosphate (PtdIns3P) which stimulates kinase activity; required for overflow degradation of misfolded proteins when ERAD is saturated |
| **SPT8** | na | na | na | Subunit of the SAGA transcriptional regulatory complex; not present in SAGA-like complex SLIK/SALSA; required for SAGA-mediated inhibition at some promoters |
| **APL2** | na | na | na | Beta-adaptin subunit of the clathrin-associated protein (AP-1) complex; binds clathrin; involved in clathrin-dependent Golgi protein sorting; protein abundance increases in response to DNA replication stress |
| **RAD2** | na | na | na | Single-stranded DNA endonuclease; cleaves single-stranded DNA during nucleotide excision repair to excise damaged DNA; subunit of Nucleotide Excision Repair Factor 3 (NEF3); homolog of human XPG protein |
| **YNR068C** | na | na | na | hypothetical protein; exhibits homology to C-terminal end of Bul1p; expressed as a readthrough product of BSC5, the readthrough locus being termed BUL3; the BUL3 readthrough product is involved in ubiquitin-mediated sorting of plasma membrane proteins and interacts with WW domains of Rsp5p in vitro, but in a functionally different way than the non-readthrough form |
| **ECM31** | na | na | na | Ketopantoate hydroxymethyltransferase; required for pantothenic acid biosynthesis, converts 2-oxoisovalerate into 2-dehydropantoate |
| **STE2** | na | na | na | Receptor for alpha-factor pheromone; seven transmembrane-domain GPCR that interacts with both pheromone and a heterotrimeric G protein to initiate the signaling response that leads to mating between haploid a and alpha cells |
| **CLB1** | na | na | na | B-type cyclin involved in cell cycle progression; activates Cdc28p to promote the transition from G2 to M phase; accumulates during G2 and M, then targeted via a destruction box motif for ubiquitin-mediated degradation by the proteasome; CLB1 has a paralog, CLB2, that arose from the whole genome duplication |
| **KEX1** | na | na | na | Cell death protease essential for hypochlorite-induced apoptosis; involved in the processing of killer toxin and alpha factor precursor; cleaves Lys and Arg residues from the C-terminus of peptides and proteins |
| **UBX6** | na | na | na | UBX (ubiquitin regulatory X) domain-containing protein; interacts with Cdc48p, transcription is repressed when cells are grown in media containing inositol and choline; UBX6 has a paralog, UBX7, that arose from the whole genome duplication |
| **VPS33** | na | na | na | ATP-binding protein that is a subunit of the HOPS and CORVET complexes; essential for protein sorting, vesicle docking, and fusion at the vacuole; binds to SNARE domains |
| **APQ13** | na | na | na | No Description Found |
| **NPL6** | na | na | na | Component of the RSC chromatin remodeling complex; interacts with Rsc3p, Rsc30p, Ldb7p, and Htl1p to form a module important for a broad range of RSC functions |
| **BAR1** | na | na | na | Aspartyl protease; secreted into the periplasmic space of mating type a cell; helps cells find mating partners; cleaves and inactivates alpha factor allowing cells to recover from alpha-factor-induced cell cycle arrest |
| **ATG11** | na | na | na | Adapter protein for pexophagy and the Cvt targeting pathway; directs receptor-bound cargo to the phagophore assembly site (PAS) for packaging into vesicles; required for recruiting other proteins to the PAS; recruits Dnm1p to facilitate fission of mitochondria that are destined for removal by mitophagy |
| **BUD16** | na | na | na | Putative pyridoxal kinase; a key enzyme involved in pyridoxal 5'-phosphate synthesis, the active form of vitamin B6; required for genome integrity; involved in bud-site selection; similarity to yeast BUD17 and human pyridoxal kinase (PDXK) |
| **INH1** | na | na | na | Protein that inhibits ATP hydrolysis by the F1F0-ATP synthase; inhibitory function is enhanced by stabilizing proteins Stf1p and Stf2p; has a calmodulin-binding motif and binds calmodulin in vitro; INH1 has a paralog, STF1, that arose from the whole genome duplication |
| **YKL069W** | na | na | na | Methionine-R-sulfoxide reductase; reduces the R enantiomer of free Met-SO, in contrast to Ycl033Cp which reduces Met-R-SO in a peptide linkage; has a role in protection against oxidative stress; relative distribution to the nucleus increases upon DNA replication stress |
| **ISA2** | na | na | na | Protein required for maturation of mitochondrial [4Fe-4S] proteins; functions in a complex with Isa1p and possibly Iba57p; localizes to the mitochondrial intermembrane space, overexpression of ISA2 suppresses grx5 mutations |
| **COX9** | na | na | na | Subunit VIIa of cytochrome c oxidase (Complex IV); Complex IV is the terminal member of the mitochondrial inner membrane electron transport chain |
| **UGA1** | na | na | na | Gamma-aminobutyrate (GABA) transaminase; also known as 4-aminobutyrate aminotransferase; involved in the 4-aminobutyrate and glutamate degradation pathways; required for normal oxidative stress tolerance and nitrogen utilization; protein abundance increases in response to DNA replication stress |
| **MIP6** | na | na | na | Putative RNA-binding protein; interacts with Mex67p, which is a component of the nuclear pore involved in nuclear mRNA export; MIP6 has a paralog, PES4, that arose from the whole genome duplication |
| **YER158C** | na | na | na | hypothetical protein; potentially phosphorylated by Cdc28p; YER158C has a paralog, AFR1, that arose from the whole genome duplication |
